# Single-cell and bulk transcriptome-based identification of myeloid-derived suppressor cell-associated immune evasion signatures and a prognostic model for gastric cancer

**DOI:** 10.1186/s12885-026-16584-0

**Published:** 2026-07-29

**Authors:** Xinghua Zhang, Can Sun, Xi Zhang

**Affiliations:** 1 Department of Gastroenterology, Beijing Friendship Hospital, Capital Medical University, Beijing, China; 2 State Key Laboratory of Digestive Health, Beijing, China; 3 National Clinical Research Center for Digestive Disease, Beijing, China; 4 Beijing Key Laboratory of Early Gastrointestinal Cancer Medicine and Medical Devices, Beijing, China

**Keywords:** Gastric cancer, Single-cell transcriptomics, Myeloid immunosuppression, Prognostic model

## Abstract

**Background:**

Gastric cancer (GC) immunotherapy has limited efficacy. We developed a myeloid immunosuppressive niche score (GC-MIS) to quantify immune evasion and assess its association with the tumor microenvironment and patient prognosis.

**Methods:**

Two single-cell and three bulk transcriptomic cohorts were analyzed. A 12-gene MDSC-like signature (MIS) and an 8-gene tumor-intrinsic (TU) module were identified, and GC-MIS was defined as the standardized composite score of the two. Bulk cohort analyses included multivariable regression, Cox modeling, and prognostic evaluation (AUC, C-index, calibration, and decision curves). CellChat characterized epithelial–myeloid interactions, and in vitro GC cell lines exposed to inflammatory and hypoxic stimuli with pathway-specific inhibitors were assessed for PD-L1, STAT3 phosphorylation, and chemokine responses.

**Results:**

MIS and TU modules were stable and moderately correlated at the single-cell level. In bulk cohorts, GC-MIS was positively associated with T-cell exhaustion and myeloid abundance, and inversely associated with antigen presentation (adjusted R² = 0.33–0.41). High GC-MIS independently predicted worse survival (pooled HR = 1.32, *P* < 0.001). A GC-MIS–based model combined with clinical variables showed consistent but moderate prognostic performance across cohorts (5-year AUC ≈ 0.66). Pathway analyses implicated JAK–STAT, HIF-1, and PD-1/PD-L1 signaling, and GC-MIS correlated with CXCL8, IL6, and CCL2 communication axes. In vitro, inflammatory and hypoxic stimuli induced PD-L1 and STAT3 activation, which were significantly attenuated by targeted inhibitors (*P* < 0.05).

**Conclusions:**

GC-MIS is a reproducible transcriptional score associated with features of the tumor immune microenvironment and prognosis in gastric cancer. It reflects tumor–myeloid interaction programs and may serve as a prognostic biomarker. The associated inflammatory and hypoxia-linked signaling pathways warrant further investigation in functional and immunotherapy-treated cohorts.

**Supplementary Information:**

The online version contains supplementary material available at https://doi.org/10.1186/s12885-026-16584-0.

## Introduction

Gastric cancer (GC) remains one of the most common malignant tumors of the digestive tract worldwide, and many patients are diagnosed at an advanced stage. Improvements in long-term survival rely on combined strategies including surgery, chemotherapy, and targeted therapy [1, 2]. Immune checkpoint inhibitors benefit a subset of patients, but overall response rates are limited and are closely linked to the immunosuppressive state of the tumor microenvironment [3]. Myeloid cells are abundant and highly plastic in the GC microenvironment, shaping an immunosuppressive niche through inflammatory chemotaxis, metabolism, and immune checkpoint molecules, which drive T-cell exhaustion and reduce antigen presentation [4, 5]. Single-cell transcriptomics allows these states to be resolved at cellular resolution, offering insights for mechanistic studies and clinical stratification [6].

Existing studies often rely on a few markers or estimated cell infiltration, overlooking the complexity of the continuous spectrum of MDSC states. These approaches are easily confounded by tumor purity, cellular composition, and batch effects [7, 8]. Myeloid immunosuppressive features identified at the single-cell level remain largely descriptive, lack reproducible quantification in bulk cohorts, and cannot be linked clearly to T-cell exhaustion or impaired antigen presentation [9, 10]. Mechanistic validation typically requires primary myeloid cells or co-culture systems and is constrained by technical conditions and implementation cost, resulting in insufficient bridging evidence between tumor-derived driver signals and the myeloid immunosuppressive niche [11], which impairs the translation of research findings into clinical stratification markers and potential therapeutic targets. Several transcriptome-based signatures reflecting myeloid inflammation, MDSC activity, immune exclusion, or tumor–immune interactions have previously been reported in gastric cancer and other solid tumors [7]. However, most existing approaches focus primarily on immune-cell-derived markers, bulk infiltration estimates, or single biological processes. In contrast, GC-MIS was designed to integrate a suppressive myeloid program identified from reproducible single-cell-defined MDSC-like states with a tumor-derived upstream induction program. By combining these two components within a unified framework and translating them into a bulk-applicable score, GC-MIS aims to capture the biological interaction between tumor-associated signaling and suppressive myeloid states, rather than merely reflecting immune-cell abundance.

To address these gaps, we used single-cell discovery with external reproduction to distill a myeloid suppressive module and constructed a tumor upstream induction module from malignant epithelial pseudo-bulk to form the GC-MIS scoring system. We then assessed its independent associations with immune infiltration, exhaustion, and antigen presentation across multiple bulk cohorts and built a parsimonious prognostic model. Finally, through ligand–receptor communication analysis combined with induction–inhibition experiments in GC cell lines, we provided in vitro evidence of tumor-cell signaling plasticity in response to inflammatory and hypoxic stimuli. This supports the hypothesis that tumor cells may contribute to myeloid niche formation, but direct evidence of myeloid cell induction or functional polarization requires future co-culture studies.

## Materials and methods

### Study design

This study was conducted in the following sequence: single-cell discovery → external single-cell reproduction → bulk construction and validation → minimal in vitro support. First, using GC single-cell data from GSE206785 [12], we identified myeloid immunosuppression–related clusters and distilled two-layer gene modules that comprise the Myeloid Immunosuppressive Niche Score (GC-MIS). Subsequently, in independent single-cell data from GSE183904 [13], we reproduced the experimental workflow and applied the same criteria. Next, using TCGA-STAD [14], we constructed and trained a parsimonious prognostic model based on GC-MIS and performed external validation and robustness assessment in GSE62254 [15] and GSE84437 [16]. Finally, in GC cell lines, we provided in vitro corroboration of tumor-driven upstream signals using an induction–inhibition strategy. Data download and analysis were performed from March 1 to June 30, 2025, and in vitro experiments were completed from July 1 to August 31, 2025. The overall study workflow is summarized in Supplementary Figure S1, illustrating the stepwise approach from single-cell discovery to validation and in vitro functional experiments.

### Sources of public transcriptome data

We included only publicly available gastric adenocarcinoma cohorts with accessible expression profiles and clinical information, with all data downloaded from the official GEO or GDC portals. The study design comprised two layers of single-cell data (discovery and external reproduction) and three layers of bulk data (training and two external validations). At the single-cell level, GSE206785 served as the discovery cohort and GSE183904 as the external reproduction cohort. We downloaded their released cell expression matrices and accompanying metadata, harmonized gene symbols, and retained only cells derived from tumor tissue, removing technical replicate samples. At the bulk level, TCGA-STAD was used as the training cohort, with HTSeq-Counts and clinical follow-up data obtained via GDC. GSE62254 and GSE84437 served as independent external validation cohorts, from which expression matrices and follow-up information were downloaded. We collapsed probes to the gene-symbol level according to platform annotations (summarizing duplicate probes by median) and harmonized gene symbols before proceeding to downstream analyses.

Inclusion criteria were: (1) tissue explicitly identified as gastric adenocarcinoma tumor; (2) expression profiles that could be matched to patient-level clinical information, including at minimum survival time and outcome, along with usable basic clinical variables; and (3) sufficient coverage of key genes required to compute GC-MIS within the cohort to meet analytical requirements. Exclusion criteria were: (1) duplicate samples or records in which patient uniqueness could not be determined; (2) non-tumor tissue or inability to match to patient-level information; (3) missing follow-up time or unknown outcome; and (4) missing key genes in the expression matrix resulting in GC-MIS computation coverage of less than 60%.

### Single-cell preprocessing and identification of myeloid immunosuppressive clusters

Single-cell data from GSE206785 (discovery) and GSE183904 (external reproduction) were processed using the same analysis pipeline. Quality control, doublet removal, normalization, batch-effect correction, dimensionality reduction, clustering, and visualization were performed using Seurat, Harmony, and related tools. Detailed preprocessing and clustering parameters are provided in Supplementary Method 1. To reduce biases from unequal cell numbers across samples, we applied a cell-number resampling procedure prior to data integration. Samples exceeding 1.5× the median cell count were randomly downsampled to this threshold using a fixed random seed to ensure reproducibility. To assess whether this procedure altered biological composition, we compared major lineage proportions (epithelial, myeloid, T cells, B cells, fibroblasts, and endothelial cells) before and after downsampling. No substantial shifts were observed (Chi-square tests, all *P* > 0.05), indicating that resampling reduced technical imbalance without affecting underlying biological signals.

Cell-type annotation adopted a combined strategy of “marker genes + reference mapping”. Preliminary annotation was based on canonical markers: LILRB1, LST1, ITGAM, ITGAX, and CSF3R for identifying myeloid cells; EPCAM, KRT8, and KRT18 for epithelial cells; COL1A1 and COL1A2 for fibroblasts; and PECAM1 and VWF for endothelial cells. Subsequently, SingleR was used for reference mapping, with Blueprint-Encode and Monaco Immune Data used as dual reference libraries for immune and stromal cells, respectively, and a difference ≥ 0.1 between the top-1 and top-2 correlation coefficients was set as the consistency threshold [17].

Determination of the myeloid immunosuppressive cluster (MIS-cluster, also referred to as MDSC-like/inflammatory myeloid) was based on “dual module scoring” supplemented by constraints on the proportion of cells positive for key genes. We constructed an inhibitory program module (MIS-inhibitory: S100A8, S100A9, CXCL8, CXCL2, IL1B, MMP9, OLR1, LILRB2, TREM1, FCGR3B, CSF3R, S100A12, CYBB, NCF2, PADI4) and an antigen presentation module (AP-module: HLA-DRA, HLA-DRB1, HLA-DPA1, HLA-DPB1, HLA-DQA1, HLA-DQB1, CIITA, CD74, TAP1, TAPBP), and used AddModuleScore to compute cluster-level mean scores. Myeloid clusters meeting all of the following criteria were defined as MIS-clusters: (1) the MIS-inhibitory module mean was at or above the 75th percentile among all myeloid clusters; (2) the AP-module mean was at or below the 25th percentile; and (3) the proportions of S100A8-positive and S100A9-positive cells within the cluster were each ≥ 50%. These prespecified cutoffs were chosen to capture relative extremes of high suppressive programming and low antigen-presentation activity at the cluster level, while the ≥ 50% S100A8/S100A9 positivity requirement ensured majority marker positivity rather than sparse or low-level expression. Consistency of MIS-cluster identification across two independent single-cell cohorts and stability of MIS-module scoring patterns across datasets provided empirical support for the robustness of the definition. For identified MIS-clusters, we recorded their reproducibility and cellular proportions across both single-cell cohorts and generated UMAP spatial distributions and feature-gene expression plots.

On this basis, differential analysis was performed between MIS-clusters and the remaining myeloid cells (Wilcoxon; Benjamini-Hochberg adjusted q < 0.01, |log2FC|≥0.5), and the intersection across the two single-cell cohorts was taken to obtain a stable set of differentially expressed genes. The stable differentially expressed genes were then subjected to Spearman correlation co-expression clustering, using hierarchical clustering with a cut height of 0.5 to determine co-expression module boundaries, which served as the candidate gene pool for subsequent MIS-module construction.

### Construction and validation of GC-MIS

#### Construction of the myeloid suppressive module (MIS-module)

Within the candidate pool, 100 rounds of bootstrap resampling were performed (each time randomly sampling 80% of cells). For each resampled dataset, the differential significance and mean expression ranking of candidate genes were calculated. Genes with a stable selection frequency ≥ 0.80 were retained, and the top 12 genes ranked by mean log2FC were taken to constitute the final MIS-module. This module was used to quantify the strength of the myeloid suppressive niche.

#### Construction of the tumor induction module (TU-module)

After identifying malignant epithelial cells using inferCNV, gene expression was aggregated at the patient level to generate epithelial pseudo-bulk profiles. The MIS-module score was independently calculated for each patient. To identify tumor-intrinsic transcriptional programs associated with the suppressive myeloid state, spearman correlation analysis was performed between epithelial gene expression and the MIS-module score across patients. This step was used as an exploratory association analysis to prioritize tumor-derived signals linked to myeloid suppressive activity, rather than to construct an independent predictive signature. Genes annotated to GO:0005576 (extracellular region) or GO:0005886 (plasma membrane) were retained to enrich for secreted or membrane-associated molecules potentially involved in tumor–immune communication. LASSO regression was then applied to reduce redundancy and select a parsimonious set of representative genes, resulting in an 8-gene TU-module. Importantly, this selection process was performed independently of any clinical outcome data. The resulting TU-module represents a tumor-intrinsic transcriptional program, rather than one derived from, the MIS-module. Prespecified biological pathways of interest included IL6/JAK-STAT3, HIF-1α/hypoxia, COX-2/PGE2, chemokine, and immune checkpoint signaling axes.

#### GC-MIS calculation and cross-cohort reproduction

At the single-cell level, AddModuleScore was used to calculate cell-level scores for the two modules, and the patient-level median was taken as the patient GC-MIS component score. At the bulk level, GSVA’s ssGSEA was used to calculate sample-level scores for the two modules. GC-MIS was defined as z-score(MIS-module score) + z-score(TU-module score) [18]. The above workflow was independently reproduced in the pseudo-bulk of GSE206785 and the single-cell data of GSE183904, and the directional consistency of distributions and correlations between the two cohorts was examined.

### Bulk transcriptome data preprocessing and tumor microenvironment characterization

To avoid bias introduced by cross-platform merging, preprocessing, scoring, and statistical analyses were completed independently within each bulk cohort, and parameter migration was performed only at the model application stage. TCGA-STAD used raw counts, converted to TPM by gene length, followed by log2(TPM + 1) transformation; GSE62254 and GSE84437 used limma for background correction and quantile normalization, followed by log2 transformation. All genes were unified to HGNC symbols and duplicate symbols were removed.

Tumor microenvironment characterization employed two complementary approaches. The first used MCP-counter to calculate single-sample abundances of monocytes, myeloid cells, T cells, B cells, fibroblasts, and endothelial cells [19]. The second used CIBERSORTx (LM22) to estimate fine immune cell proportions at the single-sample level [20]. Both results were standardized to Z scores and used for correlation and sensitivity analyses. Features of antigen presentation, T-cell exhaustion, and related pathways were scored using GSVA. The T-cell exhaustion gene set comprised PDCD1, CTLA4, HAVCR2, LAG3, TIGIT, CXCL13, TOX, PRDM1, and EOMES; the antigen presentation gene set comprised B2M, HLA-A, HLA-B, HLA-C, TAP1, TAP2, TAPBP, PSMB8, and PSMB9; interferon and hypoxia signaling used the IL6_JAK_STAT3_SIGNALING and HYPOXIA gene sets from MSigDB Hallmark. Tumor purity was estimated within each cohort using ESTIMATE, and clinical variables were harmonized for age, sex, and pathological stage.

### Association analysis of GC-MIS with tumor microenvironment and clinical features

Correlation and regression analyses were performed separately within TCGA-STAD, GSE62254, and GSE84437. First, Spearman correlations between GC-MIS and MCP-counter immune cell abundances, CIBERSORTx cell proportions, and GSVA pathway scores were computed and corrected according to the multiple-testing control strategy in 2.10. Subsequently, multivariable linear regression models were constructed with GC-MIS as the dependent variable and tumor purity, stage, and neutrophil abundance as independent variables to test the independent association between GC-MIS and suppressive tumor microenvironment phenotypes. To verify robustness, stratified analyses were performed by stage, MSI status, and Lauren classification (in cohorts providing this variable), and directional consistency was reported.

### Construction and validation of a GC-MIS–based prognostic model

In the TCGA-STAD training set, a Cox proportional hazards model was used to evaluate the association between GC-MIS and survival, with overall survival as the primary endpoint and progression-free survival as the secondary endpoint. The prognostic model was prespecified to include GC-MIS, age, and pathological stage because of their biological and clinical relevance. Automated feature-selection procedures were not used. The number of events and event-per-variable ratios were evaluated to minimize overfitting risk. Model covariates were GC-MIS (continuous z-score), pathological stage (I–IV, coded as an ordered variable from 1 to 4), and age (continuous). Martingale residuals were used to test the linearity assumption, and, when necessary, 3-knot restricted cubic splines were applied to GC-MIS and age. Schoenfeld residuals were used to test the proportional hazards assumption. A research-type risk score was constructed based on the linear predictor, and the median in the training set was used as the dichotomization threshold; this threshold was directly applied in the two external validation cohorts for survival stratification. Model discrimination was assessed by Harrell’s C-index and time-dependent ROC (1-year, 3-year, 5-year), and calibration was assessed by calibration curves with 1,000 bootstrap resamples. Decision curve analysis was used to display potential clinical net benefit. Hazard ratios and 95% confidence intervals were reported, and the directional consistency of effect sizes from fixed-effects pooling across cohorts was assessed. Moreover, to assess the robustness of the prognostic association, prespecified subgroup analyses were performed according to pathological stage (I–II vs. III–IV), MSI status (MSI vs. MSS), and Lauren classification where available. Hazard ratios and 95% confidence intervals were estimated within each subgroup. Effect modification was evaluated by including GC-MIS × subgroup interaction terms in Cox proportional hazards models, and interaction P values were reported. GC-MIS was analyzed primarily as a continuous variable in all survival analyses. In Cox proportional hazards models, GC-MIS was standardized as a z-score and evaluated per 1-standard-deviation increase to preserve full information and avoid the loss of statistical power associated with categorization. Multivariable Cox models were adjusted for age and pathological stage in each cohort.

### Molecular pathway enrichment and ligand–receptor interaction analysis

In TCGA-STAD, samples were divided into high and low groups by the median of GC-MIS, and limma was used to compare differential expression with thresholds of q < 0.05 and |log2FC|≥0.5. Based on differentially expressed genes, clusterProfiler was used for GO Biological Process and KEGG pathway enrichment analyses, and the false discovery rate was controlled using the multiple-testing control strategy in 2.10. At the single-cell level, CellChat was used to infer cell-cell communication, restricting the sender to malignant epithelial cells and the receiver to myeloid cells, with a focus on testing the signal strength and contribution of the IL6–IL6R, CXCL8–CXCR1/2, CCL2–CCR2, VEGFA–KDR, LGALS9–HAVCR2, and CD274–PDCD1 pathways, and independently reproducing these analyses in the two single-cell cohorts. Cell–cell communication analysis was performed using CellChat separately in each single-cell cohort (GSE206785 and GSE183904). To ensure robustness, ligand–receptor interactions were retained only if they met the following criteria: (1) detectable expression (≥ 10% of cells in sender or receiver populations), (2) CellChat default permutation-based significance (*P* < 0.05), and (3) consistency of interaction ranking across both cohorts. Interactions were further prioritized based on biological plausibility focusing on inflammation, chemotaxis, hypoxia, and immune checkpoint pathways. To assess inter-patient heterogeneity, communication strength was summarized at the patient level and compared using coefficient of variation (CV) and correlation with GC-MIS scores across samples. All communication strengths were presented as standardized scores and correlated with GC-MIS levels.

### Gastric cancer cell lines and in vitro induction–inhibition experiments

In vitro validation was performed in three GC cell lines: AGS, MKN45, and HGC-27. All cell lines underwent STR authentication and were confirmed mycoplasma-negative. Detailed cell culture conditions and cell-line information are provided in Supplementary Methods S2.

Stimulation protocols included IFN-γ, IL-6, Oncostatin M, TNF-α, IL-1β, and CoCl₂ induction, as well as pharmacologic inhibition using ruxolitinib, Stattic, and celecoxib. Detailed treatment conditions and experimental procedures are provided in Supplementary Methods S3.

Readouts included qRT-PCR, Western blotting, and ELISA. Detailed assay protocols, reagent information, and cycling conditions are provided in Supplementary Methods S4. Data from any inhibitor treatment that resulted in a > 20% reduction in cell viability were excluded from the analysis. All experiments were performed in three independent biological replicates per condition and cell line, with each replicate representing a separate culture experiment performed at different passages. No technical replicates were included.

### Statistical analysis

All analyses were performed in R 4.3.2 and Python 3.11. Key package versions were Seurat 4.4.0, Harmony 0.1.1, SingleR 2.4.1, GSVA 1.50.3, limma 3.58.1, sva 3.48.0, survival 3.5-7, rms 6.7-1, timeROC 0.4, CellChat 1.6.1, MCPcounter 1.2.0, and CIBERSORTx. Two-group comparisons of continuous variables used two-sided Wilcoxon rank-sum tests, and multiple-group comparisons used the Kruskal–Wallis test with Benjamini–Hochberg correction. Correlation analyses used the Spearman method. Covariates for multivariable linear regression and Cox regression were kept consistent within cohorts. Missing values were not imputed, and complete-case analysis was used. For the bulk cohorts, excluded cases and variables with missing data were summarized for each cohort. Missingness was assessed descriptively with respect to stage and outcome when metadata were available. Because missingness primarily resulted from unavailable follow-up or incomplete linkage prior to model fitting, no formal imputation or modeling of the mechanisms underlying missingness was performed. The target false discovery rate for multiple-testing control was 0.05. Survival analyses presented stratified differences using Kaplan–Meier curves and log-rank tests, with hazard ratios and 95% confidence intervals reported. Discrimination was assessed using Harrell’s C-index and AUC from timeROC, and calibration was assessed using 1,000 bootstrap resamples. Overall survival (OS) was defined as the time from diagnosis (TCGA-STAD) or from the date of sample collection/enrollment (GEO cohorts) to death from any cause. Patients who were alive at the last follow-up were censored at that time point.

## Results

### GC-MIS originates from reproducible myeloid immunosuppressive clusters

**Fig. 1:**
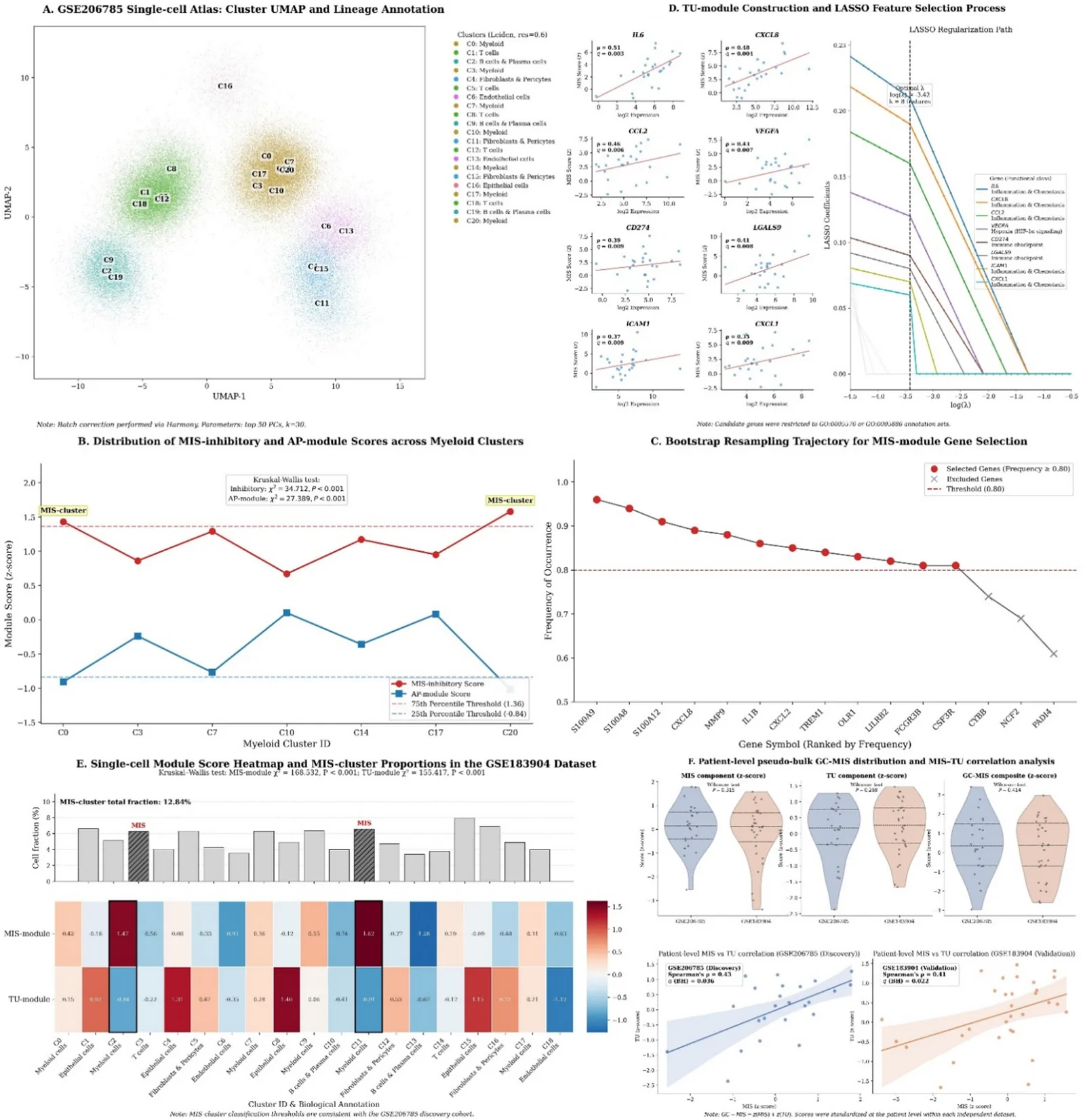
Single-cell discovery and external validation workflow of GC-MIS in gastric cancer. **A** UMAP of GSE206785 single-cell transcriptome after Harmony integration (21 Leiden clusters; resolution = 0.6, PC = 50, k = 30). Colors indicate major cell lineages: epithelial, myeloid, T cells, B/plasma cells, fibroblasts/pericytes, and endothelial cells. **B** MIS-inhibitory and antigen presentation (AP) module scores across myeloid clusters in GSE206785. Statistical differences were tested using Kruskal–Wallis test (*P* < 0.001). **C** Bootstrap resampling analysis (100 iterations, 80% cells per iteration) for MIS-module gene selection. Genes selected ≥ 80% of the time were retained. **D** Tumor-derived induction (TU) module construction from malignant epithelial pseudo-bulk expression (GSE206785). Genes were selected based on Spearman correlation with MIS-module (BH-adjusted q < 0.01) and refined using LASSO (optimal λ = −3.42). **E** External validation in GSE183904 showing MIS-module and TU-module scores across clusters. Myeloid clusters meeting criteria for high MIS-inhibitory and low AP scores were defined as MIS-clusters (12.84%). Kruskal–Wallis test *P* < 0.001. **F **Patient-level pseudo-bulk GC-MIS distribution and correlation between MIS and TU modules in GSE206785 and GSE183904 after z-score normalization. No significant cohort differences (Wilcoxon *P* > 0.05). Spearman correlations: GSE206785 ρ = 0.43, q = 0.036; GSE183904 ρ = 0.41, q = 0.022

A total of 5 public cohorts were included. Two were GC single-cell transcriptome cohorts used for GC-MIS discovery and external reproduction, and 3 were bulk transcriptome cohorts (1 TCGA RNA-seq and 2 microarrays), all of which provided usable clinical follow-up with OS endpoints in the samples included for analysis. The ACRG cohort additionally provided RFS information (Table 1). In GSE206785, after Harmony correction, Leiden clustering (resolution = 0.6; PC = 50; k = 30) yielded 21 clusters. UMAP showed that clusters could be assigned to major lineages including epithelial, myeloid, T cells, B cells (including plasma cells), fibroblasts (including pericytes), and endothelial cells and were relatively separated, with myeloid and T cells having the highest proportions (Fig. 1A).

**Table 1:** Overview of platforms, sample sizes, and clinical endpoints for each dataset

Indicator	GSE206785	GSE183904	TCGA-STAD	GSE62254 (ACRG)	GSE84437
Platform type	Single-cell transcriptome (10x Genomics; Illumina HiSeq 4000 sequencing)	Single-cell transcriptome (Illumina NovaSeq 6000 sequencing; droplet-based)	bulk RNA-seq (Illumina HiSeq)	Microarray (Affymetrix HG-U133 Plus 2.0; GPL570)	Microarray (Illumina HumanHT-12 v3; GPL6947)
Sample type composition	Tumor + adjacent tissue (single-cell)	Primary tumor ± adjacent tissue; some samples are peritoneal lesions (single-cell)	Tumor (bulk)	Tumor (bulk)	Tumor (bulk)
Sample size (n)	24 (patients); 48 (samples)	31 (patients); 40 (samples)	350 (included in OS analysis)	300	433 (included in OS analysis; total samples 483)
Cases excluded from primary OS analysis	NA	NA	25 of 375 tumors	0 of 300	50 of 483 samples
Follow-up endpoints	NA	NA	OS (DFS/PFS-related fields available from TCGA CDR)	OS; RFS	OS
Availability of clinical variables	Age: not public; Sex: not public; Stage: not public; Molecular subtype: not provided	Stage: available; Histologic subtype: available; Age: not public; Sex: not public	Age: available; Sex: available; Stage: available; Molecular subtype: not uniformly provided	Age: available; Sex: available; Stage: available; Molecular subtype: ACRG four-subtype available	Age: available; Sex: available; Stage: available; Molecular subtype: not provided
Variables with missingness affecting primary analysis	Not applicable (no survival modeling)	Not applicable (no survival modeling)	OS time/status, age, and stage	No material missingness in OS, age, or stage among included cases	OS time/status linkage/follow-up
Missingness pattern relative to stage/outcome	Not applicable	Not applicable	Descriptively reviewed; no clear stage-related enrichment was observed	Not applicable	Mainly follow-up/status-related; no clear stage-related enrichment among records with available stage
Expression matrix type	Normalized single-cell expression matrix (processed UMI counts) (NCBI)	Processed cell × gene count table (normalized matrix)	Raw counts (GDC HTSeq-Counts)	Normalized matrix (RMA)	Normalized matrix (quantile normalization/log transformation)

Among myeloid cells in GSE206785, MIS-inhibitory and AP-module scores differed significantly across clusters (Kruskal–Wallis tests, both *P* < 0.001); only C0 and C20 simultaneously showed elevated MIS-inhibitory scores and reduced AP-module scores and were therefore identified as myeloid immunosuppressive clusters (Fig. 1B). After 100 rounds of bootstrap resampling of candidate genes (each time sampling 80% of cells), 12 genes had selection frequencies ≥ 0.80 and were stably included in the MIS-module (S100A8, S100A9, S100A12, CXCL8, MMP9, IL1B, CXCL2, TREM1, OLR1, LILRB2, FCGR3B, CSF3R), whereas genes with lower selection frequencies such as CYBB, NCF2, and PADI4 were excluded (Fig. 1C). In GSE206785 and GSE183904, 12 genes were stably incorporated into the MIS-module, and compared with other myeloid cells, these genes were consistently upregulated in the MIS-cluster (mean log2FC range 0.69–1.94); bootstrap selection frequencies were all ≥ 0.81, with effect directions entirely consistent, predominantly involving inflammation–oxidative stress and immunosuppression-related molecules such as S100A8/A9/A12, CXCL8, IL1B, and MMP9 (Table 2). At the malignant epithelial pseudo-bulk level (candidate genes restricted to GO:0005576/GO:0005886), IL6, CXCL8, CCL2, VEGFA, CD274, LGALS9, ICAM1, and CXCL1 were moderately positively correlated with the MIS-module mean (ρ = 0.35–0.51, BH-adjusted q < 0.01), and their coefficients remained nonzero at the selected λ (logλ=−3.42), and were finally determined as the TU-module (Fig. 1D). In the external single-cell cohort GSE183904, MIS-module and TU-module scores differed significantly across clusters (Kruskal–Wallis tests, both *P* < 0.001). Among them, myeloid clusters C2 and C11 showed the highest MIS-module scores and were again determined to be MIS-clusters according to the predefined rules (proportion 12.84%) (Fig. 1E). After z-standardization within each cohort, differences in patient-level distributions of MIS, TU, and GC-MIS were all nonsignificant between the two cohorts (Wilcoxon tests all *P* > 0.05), suggesting good comparability of the scores in the two independent single-cell pseudo-bulk datasets; within each cohort, MIS and TU were moderately positively correlated (GSE206785: ρ = 0.43, q = 0.036; GSE183904: ρ = 0.41, q = 0.022) (Fig. 1F).

**Table 2:** Final 12-gene list of the MIS-module and summary of effect directions across datasets

Gene	Mean log2FC (GSE206785)	Mean log2FC (GSE183904)	Selection frequency	Functional annotation category
S100A9	1.94	1.73	0.96	oxidative stress
S100A8	1.87	1.65	0.94	oxidative stress
S100A12	1.52	1.31	0.91	oxidative stress
CXCL8	1.78	1.56	0.89	inflammation/chemotaxis
MMP9	1.34	1.12	0.88	inflammation/chemotaxis
IL1B	1.67	1.49	0.86	inflammation/chemotaxis
CXCL2	1.29	1.08	0.85	inflammation/chemotaxis
TREM1	0.93	0.71	0.84	inflammation/chemotaxis
OLR1	1.12	0.97	0.83	immunosuppression
LILRB2	1.21	1.05	0.82	immunosuppression
FCGR3B	1.08	0.86	0.81	inflammation/chemotaxis
CSF3R	0.88	0.69	0.81	inflammation/chemotaxis

All genes met the prespecified differential thresholds (q < 0.01, |log2FC|≥0.5)

### GC-MIS characterizes a suppressive tumor microenvironment

**Fig. 2:**
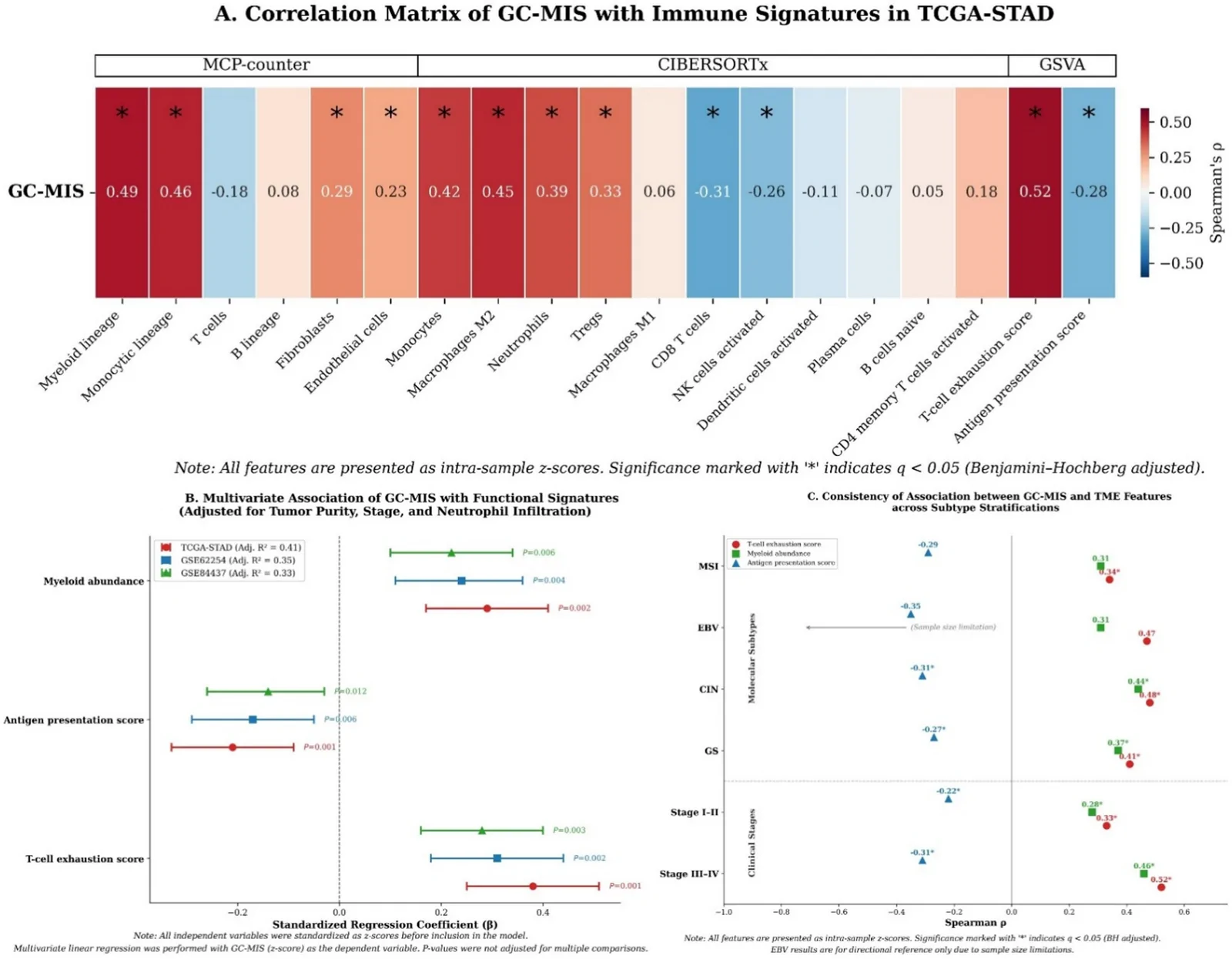
Associations of GC-MIS with tumor microenvironment functional signatures and robustness across cohorts and subtypes. (**A**) Spearman correlation matrix between GC-MIS and tumor microenvironment (TME) signatures in the TCGA-STAD cohort. Signatures include immune cell abundance estimates (MCP-counter), functional immune states, and pathway scores. Color intensity represents correlation coefficient (ρ); red indicates positive and blue indicates negative correlations. Statistical significance was assessed using false discovery rate (FDR)-adjusted q values (< 0.05). (**B**) Multivariable linear regression analysis of GC-MIS with TME functional signatures across TCGA-STAD, GSE62254, and GSE84437 cohorts. Models were adjusted for tumor purity, pathological stage, and neutrophil abundance. Effect sizes are presented as standardized regression coefficients (β), with 95% confidence intervals. Model explanatory power ranged from 0.33 to 0.41 across cohorts (adjusted R²). (**C**) Stratified analysis showing consistency of GC-MIS associations with key TME features across molecular subtypes (MSI, CIN, GS, EBV) and pathological stages (I–II vs. III–IV). Associations were assessed using Spearman correlation (ρ), and only statistically significant relationships (FDR q < 0.05) are displayed. Directional consistency was observed across all strata, with limited statistical power in the EBV subtype due to small sample size

In TCGA-STAD, GC-MIS showed significant positive correlations with Myeloid lineage, Monocytic lineage, Fibroblasts, Endothelial cells, Monocytes, Macrophages M2, Neutrophils, Tregs, and the T-cell exhaustion score (all q < 0.05), while showing negative correlations with CD8 T cells, NK cells activated, and the Antigen presentation score (all q < 0.05) (Fig. 2A). Across the three population cohorts, GC-MIS was consistently positively associated in multivariable linear regression with the T-cell exhaustion score (β = 0.28–0.38) and Myeloid abundance (β = 0.22–0.29), and negatively associated with the Antigen presentation score (β=−0.21 to − 0.14) (all *P* < 0.05); after adjusting for tumor purity, Stage, and Neutrophils, the model adjusted R² was 0.33–0.41 (Fig. 2B). Within TCGA-STAD stratified by molecular subtype and Stage, GC-MIS remained positively correlated with the T-cell exhaustion score and Myeloid abundance in MSI, CIN, GS, and in Stage I–II and III–IV (ρ = 0.33–0.52), while being negatively correlated with the Antigen presentation score across strata (ρ=−0.22 to − 0.31). The EBV subtype showed only the same directional trend due to limited sample size (Fig. 2C).

### GC-MIS predicts poor prognosis and is externally validated

**Fig. 3:**
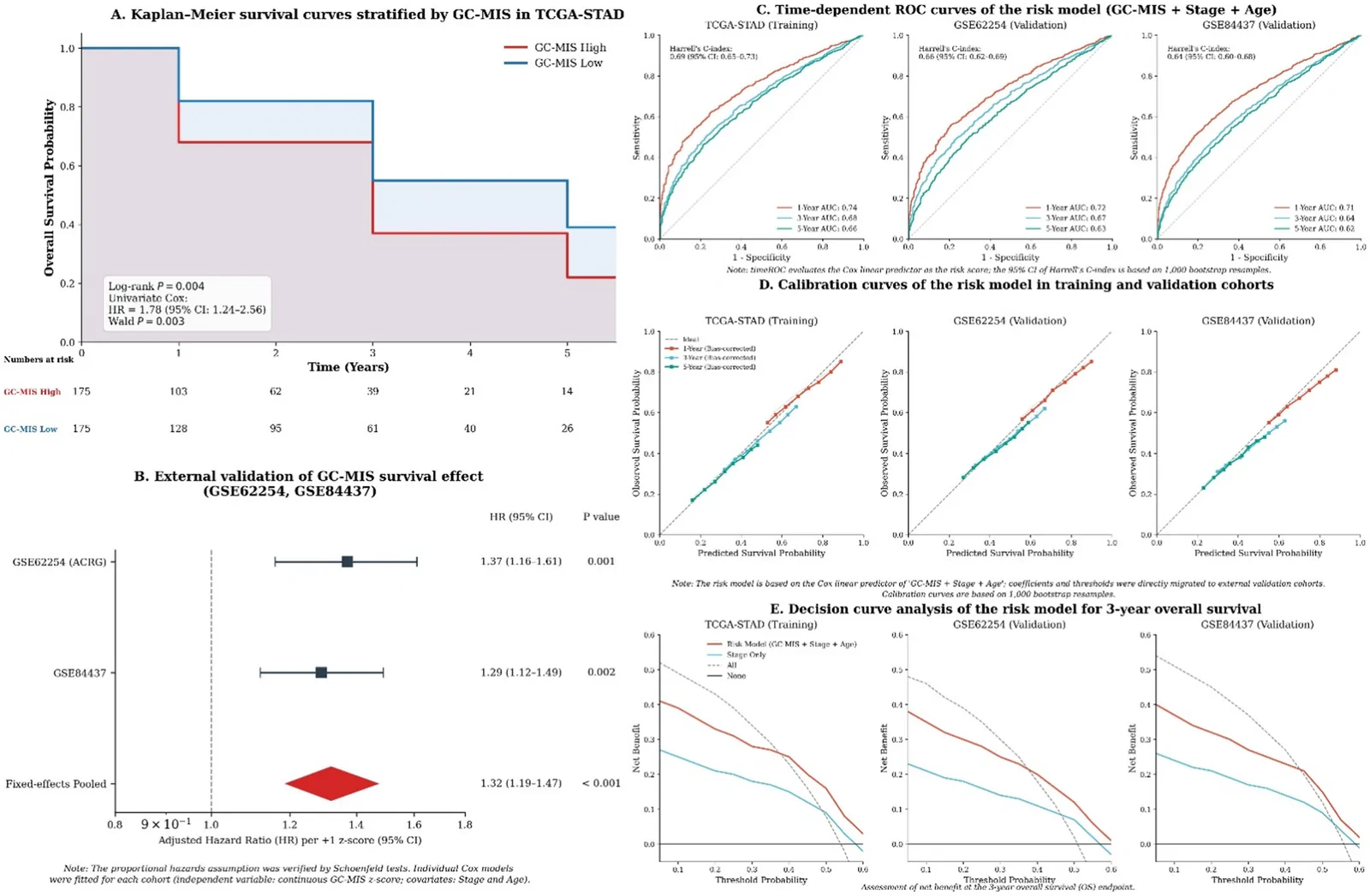
Prognostic performance and clinical utility of the GC‑MIS–based risk model. **A** Kaplan–Meier survival curves in TCGA-STAD (*n* = 350): Patients were stratified into high- and low-GC-MIS groups based on the median z-score. Overall survival (OS) differences were evaluated using the log-rank test (*P* = 0.004). **B** External validation of GC-MIS survival effect (GSE62254, *n* = 300; GSE84437, *n* = 433): Continuous GC-MIS z-scores were analyzed in multivariable Cox models adjusted for Age and Stage. Hazard ratios (HR) and 95% confidence intervals (CI) are indicated. Fixed-effect pooled HR across cohorts is shown. **C** Time-dependent ROC curves of the GC-MIS + Stage + Age risk model: 1-, 3-, and 5-year area under the curve (AUC) values are reported for TCGA-STAD, GSE62254, and GSE84437, demonstrating consistent discriminative performance across cohorts. **D** Calibration curves for predicted survival probabilities: Observed versus predicted 1-, 3-, and 5-year OS probabilities in TCGA-STAD (training) and GSE62254 and GSE84437 (validation). Calibration slopes and intercepts are shown, indicating close agreement between predictions and outcomes. **E** Decision curve analysis for 3-year OS: Net benefit of the GC-MIS + Stage + Age model compared with “Stage only,” “treat all,” and “treat none” strategies. The model demonstrates superior net benefit in mid-to-high threshold probability ranges (TCGA ≥ 0.40, GSE62254 ≥ 0.40, GSE84437 ≥ 0.45)

In the TCGA-STAD training set, the high GC-MIS group had lower OS than the low GC-MIS group (log-rank *P* = 0.004); univariable Cox analysis showed that high GC-MIS was associated with worse OS (HR = 1.78, 95% CI 1.24–2.56) (Fig. 3A). These dichotomized analyses were used for visual presentation, whereas the primary prognostic inference was based on continuous Cox modeling. The number of OS events was 146/350 in TCGA-STAD, 127/300 in GSE62254, and 191/433 in GSE84437, corresponding to events-per-variable ratios of 48.7, 42.3, and 63.7 for the three-covariate model, respectively. The final prognostic model included three prespecified predictors (GC-MIS, age, and pathological stage). In the TCGA-STAD training cohort, 146 overall survival events were available among 350 patients, corresponding to an events-per-variable ratio of 48.7, indicating an adequate sample size for model development (Supplementary Table S1). In the external validation cohorts (GSE62254 and GSE84437), after adjusting for Age and Stage, GC-MIS (per 1 z-score increase) was positively associated with OS risk (HR = 1.37 and 1.29, both *P* < 0.01), and the fixed-effect pooled HR was 1.32 (*P* < 0.001) (Fig. 3B). To assess whether differences in stage distribution across cohorts affected model performance, we repeated the validation analysis restricted to Stage II–III patients (the most represented stages across all three datasets). After restriction, C-indices remained stable: TCGA-STAD (*n* = 262) C-index = 0.68; GSE62254 (*n* = 237) C-index = 0.65; GSE84437 (*n* = 355) C-index = 0.63, compared to full-cohort estimates of 0.69, 0.66, and 0.64, respectively (Supplementary Table S2). This indicates that model performance is not substantially driven by cross-cohort stage heterogeneity.

Subgroup analyses demonstrated that the association between GC-MIS and overall survival was generally consistent across clinically relevant patient subsets (Supplementary Table S3). In patients with stage I–II disease (*n* = 437; 129 events), each 1-standard-deviation increase in GC-MIS was associated with a 24% higher risk of death (HR = 1.24, 95% CI 1.03–1.50, *P* = 0.025), while a similar association was observed in stage III–IV disease (*n* = 646; 335 events; HR = 1.31, 95% CI 1.12–1.53, *P* = 0.001). Comparable effect sizes were also observed across MSI status and Lauren classification subgroups, with hazard ratios ranging from 1.16 to 1.43 per 1-standard-deviation increase in GC-MIS. Formal interaction testing revealed no significant effect modification by pathological stage (P for interaction = 0.659), MSI status (P for interaction = 0.506), or Lauren classification (P for interaction = 0.448–0.844), supporting the robustness of GC-MIS prognostic value across different clinicopathological subsets.

A risk model constructed from GC-MIS, Stage, and Age yielded 1-, 3-, and 5-year AUCs of 0.74 (95% CI 0.68–0.79), 0.68 (95% CI 0.63–0.74), and 0.66 (95% CI 0.60–0.72) in TCGA-STAD; 0.72 (95% CI 0.66–0.78), 0.67 (95% CI 0.60–0.73), and 0.63 (95% CI 0.57–0.69) in GSE62254; and 0.71 (95% CI 0.66–0.77), 0.64 (95% CI 0.59–0.70), and 0.62 (95% CI 0.57–0.68) in GSE84437. The C-index was 0.69 (95% CI 0.65–0.73), 0.66 (95% CI 0.62–0.69), and 0.64 (95% CI 0.60–0.68) in TCGA-STAD, GSE62254, and GSE84437, respectively, indicating consistent discriminative performance in both the training and external cohorts (Fig. 3C). Detailed discrimination metrics, including C-index and time-dependent AUCs with 95% confidence intervals across models, are provided in Supplementary Table S4. The training cohort, therefore, provided an adequate event count relative to the complexity of the prespecified model. Bootstrap internal validation demonstrated minimal optimism, with a shrinkage factor of 0.96 and an optimism-corrected C-index of 0.68, suggesting a low risk of model overfitting.

In TCGA-STAD, the model’s predicted survival probabilities at 1, 3, and 5 years were generally close to the observed values, with only mild overestimation at the high-risk end; after transferring the same model coefficients to GSE62254 and GSE84437, calibration at each time point remained close to the ideal reference line, with 3-year calibration slope/intercept values of 0.97/−0.02, 0.94/0.04, and 0.91/−0.04, respectively (Fig. 3D). Calibration performance, including calibration slope, intercept, and expected-to-observed ratios at 1-, 3-, and 5-year time points, is provided in Supplementary Table S5. In decision curve analysis for 3-year OS, the GC-MIS+Stage + Age model performed better in the mid-to-high threshold probability range (approximately ≥ 0.40 in TCGA-STAD and GSE62254, and approximately ≥ 0.45 in GSE84437), with net benefit exceeding the “Stage only” as well as “treat all” and “treat none” strategies (Fig. 3E).

### GC-MIS is associated with inferred tumor–myeloid signaling pathways

**Fig. 4:**
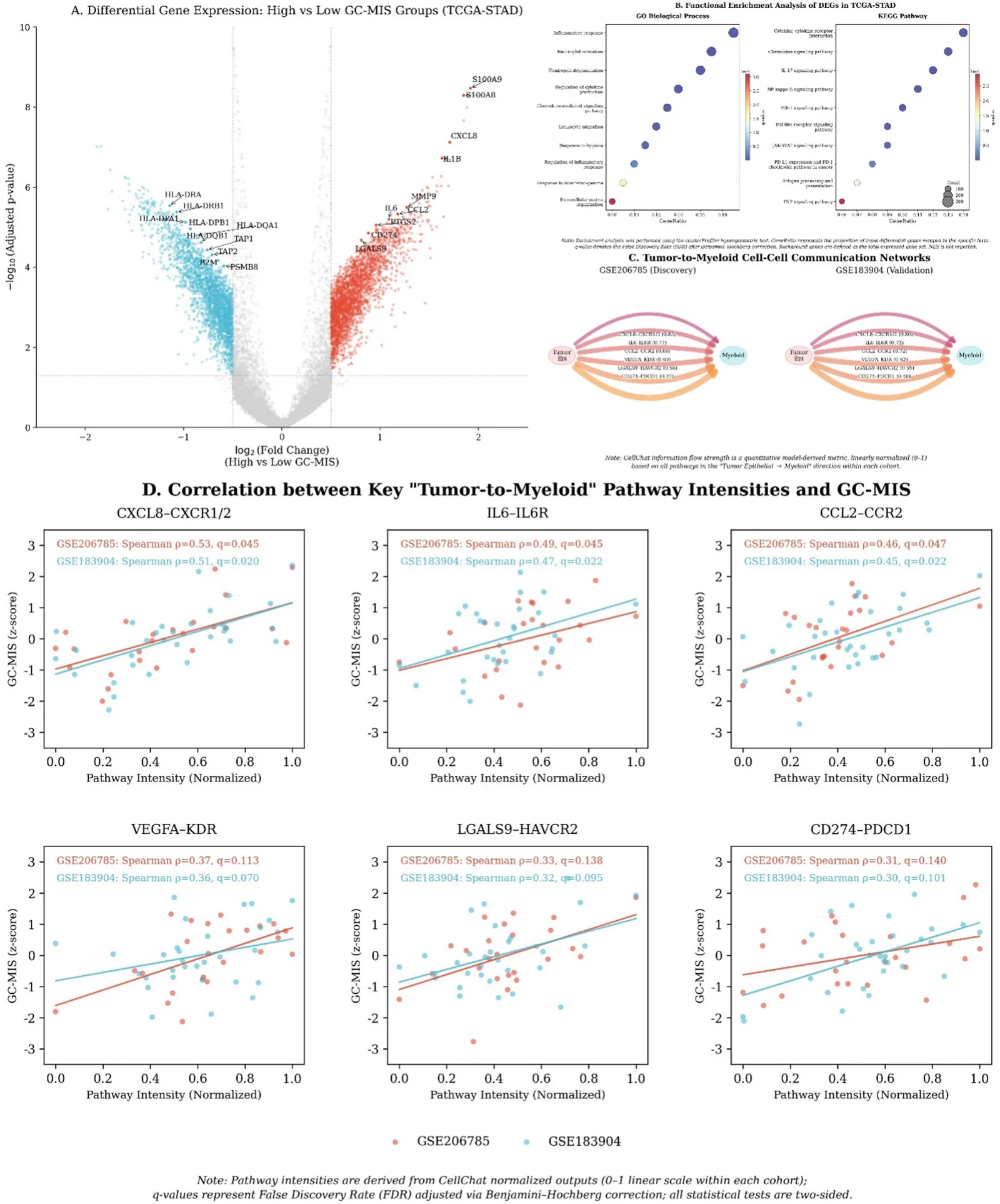
Molecular features and tumor-to-myeloid communication underlying GC-MIS. **A**. Differential gene expression analysis between high and low GC-MIS groups in TCGA-STAD (cutoff: median split). Volcano plot shows significantly upregulated (red) and downregulated (blue) genes defined by |log2FC| ≥ 0.50 and FDR-adjusted q < 0.05; key immune-related genes are annotated. **B**. GO/KEGG enrichment analysis of differentially expressed genes in TCGA-STAD. Dot size represents gene ratio, and color indicates FDR-adjusted enrichment significance (q value). Pathways are grouped into upregulated (left) and downregulated (right) functional categories. **C**. Inferred tumor-epithelial–to–myeloid cell communication networks in GSE206785 and GSE183904 single-cell cohorts. Edge thickness represents mean interaction strength calculated by ligand–receptor expression probability; node size reflects total signaling activity of each cell group; edge color denotes signaling source (tumor epithelium) to target myeloid subsets. Only interactions with statistically significant ligand–receptor expression (permutation-adjusted *P* < 0.05) are shown. **D**. Correlation between key tumor-to-myeloid signaling pathway intensities and GC-MIS across GSE206785 and GSE183904. Correlations were computed using Spearman’s rank test; points represent cohort-level estimates. Lines indicate fitted regression trends. Significance levels are adjusted using Benjamini–Hochberg correction (q < 0.05 considered significant)

To evaluate robustness across samples, ligand–receptor interaction strengths were examined at the patient level in both single-cell cohorts. The majority of epithelial–myeloid signaling pathways demonstrated low-to-moderate inter-patient variability, with consistent ranking patterns across individuals, particularly for CXCL8–CXCR1/2 and IL6–IL6R axes. In contrast, immune checkpoint–related interactions (CD274–PDCD1 and LGALS9–HAVCR2) showed greater heterogeneity and lower overall interaction strength. Importantly, key inflammatory and chemotactic pathways were consistently associated with GC-MIS across cohorts, supporting their robustness as core epithelial–myeloid signaling axes (Supplementary Table S6). In TCGA-STAD, after grouping by the median of GC-MIS, a total of 1,032 genes were upregulated and 847 genes were downregulated in the high group relative to the low group (|log2FC|≥0.50, q < 0.05). Upregulated genes were predominantly inflammation- and immunosuppression-related molecules, including S100A8/A9, CXCL8, IL1B, CCL2, PTGS2, CD274, and LGALS9, whereas antigen presentation-related genes, including HLA-DRA/DRB1, HLA-DPA1/DPB1, and TAP1/2, were overall downregulated (Fig. 4A). GO/KEGG enrichment based on differentially expressed genes between high and low GC-MIS groups showed that upregulated genes were mainly concentrated in inflammatory response, neutrophil activation, cytokine/chemokine signaling, IL-17, NF-kappa B, HIF-1, JAK-STAT, and PD-1/PD-L1 pathway, while antigen processing and presentation was significantly enriched in the downregulated gene set (Fig. 4B). In the two single-cell datasets, tumor-epithelial-to-myeloid inferred communication was strongest on CXCL8–CXCR1/2 and IL6–IL6R, followed by CCL2–CCR2 and VEGFA–KDR, with LGALS9–HAVCR2 and CD274–PDCD1 relatively weaker; the ranking of these six pathways was consistent across the two cohorts (Fig. 4C). In the two single-cell datasets (GSE206785, GSE183904), GC-MIS was moderately positively correlated with the intensities of the CXCL8–CXCR1/2, IL6–IL6R, and CCL2–CCR2 pathways (ρ = 0.45–0.53, BH-adjusted q < 0.05), whereas VEGFA–KDR, LGALS9–HAVCR2, and CD274–PDCD1 showed the same direction but did not reach statistical significance (Fig. 4D). We note that these communication inferences are transcriptome-based and require experimental validation.

### Experimental support for tumor-associated inflammatory signaling

**Fig. 5:**
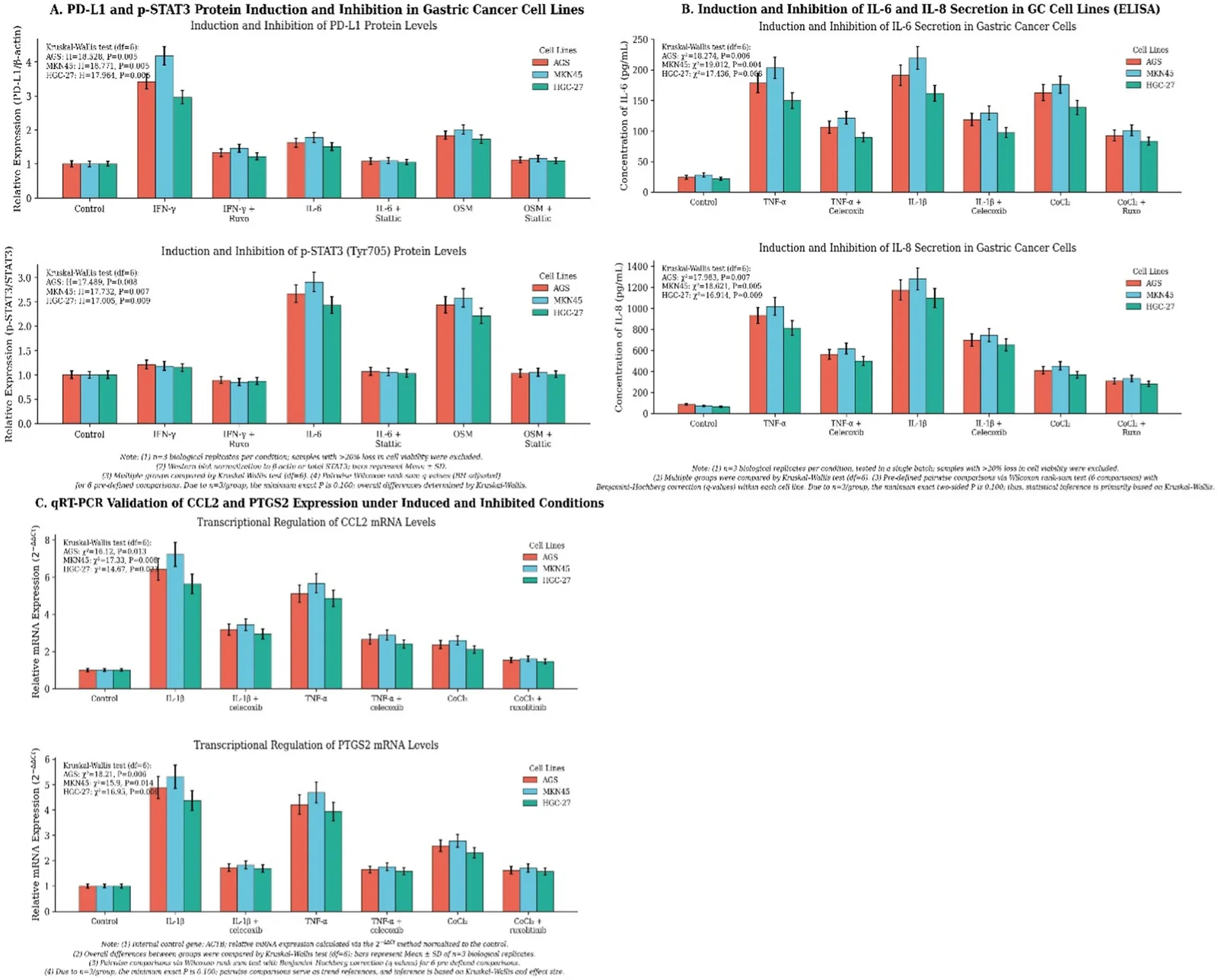
In vitro validation of inflammatory and immunosuppressive signaling programs in gastric cancer (GC) cell lines. **A** GC cell lines (AGS, MKN45, HGC-27) were stimulated with IFN-γ, IL-6, or OSM, with or without JAK/STAT pathway inhibitors (ruxolitinib or Stattic). IFN-γ induced increased PD-L1 protein expression, while IL-6 and OSM induced p-STAT3 (Tyr705) activation. Inhibitor treatment partially reversed these effects toward baseline. Statistical significance across treatment groups was assessed using Kruskal–Wallis tests (*P* < 0.01). Western blot band intensity quantification is shown as mean ± SD from three independent experiments; bar colors represent control (gray), cytokine stimulation (red), and inhibitor-treated conditions (blue). Representative immunoblots are shown in Supplementary Fig. 2, and full-length uncropped immunoblots are provided in the supplementary PDF. **B** GC cell lines treated with TNF-α, IL-1β, or CoCl₂ showed increased secretion of IL-6 and IL-8, measured by ELISA. Celecoxib or ruxolitinib significantly reduced cytokine-induced secretion levels. Differences among groups were evaluated using Kruskal–Wallis tests (*P* < 0.01). Bars represent mean ± SD of three biological replicates; colors correspond to control (gray), stimulation (red), and inhibitor-treated (blue) conditions. **C** qRT-PCR analysis of CCL2 and PTGS2 mRNA expression under inflammatory (TNF-α, IL-1β) and hypoxia-mimicking (CoCl₂) conditions, with or without celecoxib or ruxolitinib pretreatment. Both stimuli significantly upregulated gene expression, which was attenuated by inhibitor treatment (Kruskal–Wallis test, *P* < 0.05). Data are presented as mean ± SD of three independent experiments; color coding is consistent with panels A and B (gray: control, red: stimulation, blue: inhibition)

In three GC cell lines (AGS, MKN45, and HGC-27), Western blot results showed that IFN-γ stimulation increased PD-L1 protein expression, whereas IL-6 and OSM stimulation enhanced STAT3 Tyr705 phosphorylation (Fig. 5A; Supplementary Fig. 2). Addition of ruxolitinib or stattic attenuated these cytokine-induced changes, with protein expression levels shifting toward baseline. Overall differences among treatment groups were significant (Kruskal–Wallis, *P* < 0.01). These data demonstrate that GC cell lines respond to cytokine stimulation by altering checkpoint and STAT3 signaling. In three GC cell lines (AGS, MKN45, HGC-27), TNF-α and IL-1β treatments increased IL-6 and IL-8 secretion, and CoCl₂ also increased both; after addition of celecoxib or ruxolitinib, IL-6/IL-8 levels decreased (Kruskal–Wallis, *P* < 0.01) (Fig. 5B). This indicates that inflammatory and hypoxic stimuli can modulate chemokine secretion from tumor cells. In three GC cell lines (AGS, MKN45, HGC-27), IL-1β and TNF-α treatments upregulated CCL2 and PTGS2 mRNA levels, which declined after addition of celecoxib; hypoxia mimicry (CoCl₂) also induced increases in both, and expression further declined toward baseline after addition of ruxolitinib. Overall differences among treatment groups were significant across all three cell lines (Kruskal–Wallis, *P* < 0.05) (Fig. 5C). Importantly, these experiments used tumor cell lines alone and did not include myeloid cells, co-culture, chemotaxis, or T-cell suppression assays. Therefore, while tumor cells exhibit signaling plasticity in response to stimuli relevant to the tumor microenvironment, these data do not directly demonstrate induction of suppressive myeloid phenotypes or MDSC function.

## Discussion

In two GC single-cell transcriptomes, a reproducible MDSC-like immunosuppressive cluster was identified within the myeloid lineage, characterized by enhanced inhibitory programs and reduced antigen presentation. From this population, a concise myeloid suppressive gene module (MIS-module) was derived using differential expression and resampling stability. Using malignant epithelial pseudo-bulk as the tumor-side signal source, an epithelial-derived induction module enriched for secreted or membrane-associated genes was further identified, enabling GC-MIS to form a reproducible dual-component framework with consistent patterns across single-cell and pseudo-bulk analyses [21]. The novelty of GC-MIS should be interpreted in the context of existing immune suppression signatures. Previous studies have described MDSC-related signatures, inflammatory scores, immune exclusion models, and tumor–immune interaction frameworks. Rather than replacing these approaches, GC-MIS extends them by integrating both a myeloid suppressive module and a tumor-derived induction module within a single-cell-guided, bulk-applicable framework. Consequently, GC-MIS is intended to represent a composite transcriptional phenotype associated with the tumor–myeloid suppressive niche rather than a direct measure of MDSC abundance or immune infiltration [22]. By restricting the induction module to secreted or membrane-associated genes, GC-MIS highlights a potentially communicable tumor–immune interface. The in vitro experiments were limited to tumor-cell systems and were designed to assess signaling plasticity rather than myeloid function; thus, they support tumor responsiveness to inflammatory and hypoxic stimuli but do not demonstrate myeloid polarization.

GC-MIS does not reflect the abundance of immunosuppressive myeloid cells but instead represents a composite transcriptional phenotype of the tumor–myeloid suppressive niche. The MIS-module captures a functionally polarized myeloid state characterized by high inflammatory and immunosuppressive gene expression (e.g., S100A8/A9, CXCL8, IL1B) together with reduced antigen presentation activity (HLA and TAP genes). The TU-module reflects tumor-intrinsic signaling programs (IL6, CXCL8, CCL2, CD274) associated with this state. Importantly, GC-MIS remained associated with T-cell exhaustion after adjustment for myeloid cell abundance, indicating that it captures functional immunosuppressive activity rather than cellular quantity. Therefore, GC-MIS should be interpreted as a functional niche score that describes tumor–myeloid interaction activity rather than as a proxy for cell abundance.

Previous GC studies have often inferred immunosuppression using single-layer myeloid or neutrophil-related features, which are easily affected by fluctuations in tumor purity and cellular composition and are difficult to use to explain tumor-side drivers [3, 23]; although single-cell studies can observe suppressive myeloid subpopulations, they often remain descriptive and lack a framework that can be stably quantified in bulk across multiple cohorts and linked to clinical endpoints [24].

In bulk cohorts, GC-MIS was positively associated with myeloid abundance and T-cell exhaustion and negatively associated with antigen presentation, CD8 T cells, and activated NK cells. These associations remained significant after adjusting for tumor purity, stage, and neutrophil infiltration, indicating that GC-MIS reflects functional immune suppression rather than compositional differences. The consistency across MSI, CIN, GS, and stage subgroups further supports its robustness as a cross-subtype immune evasion marker [25]. Clinically, GC-MIS stratified survival in the training cohort and remained independently associated with overall survival in external cohorts after adjustment for age and stage [26]. A model combining GC-MIS, Stage, and Age maintained transferability in discrimination and calibration assessments and achieved higher net benefit in decision curve analysis within the mid-to-high threshold probability range, enabling identification of populations with high immune-evasion burden and providing a quantitative entry point for subsequent stratified studies of combination interventions. However, the predictive value of GC-MIS for immunotherapy response was not evaluated in this study and remains speculative in the absence of immunotherapy-treated cohorts. Importantly, previous prognostic models for gastric cancer have largely relied on clinical variables or single immune infiltration scores, often lacking mechanistic integration and external validation [27]. In contrast, GC-MIS incorporates single-cell–derived suppressive programs with tumor-derived signaling axes, improving both biological interpretability and prognostic robustness.

High GC-MIS phenotypes were characterized by the coordinated activation of inflammatory, chemotactic, HIF-1–related hypoxic, IL6/JAK–STAT3, and immune checkpoint pathways, alongside downregulation of antigen-processing and presentation programs, indicating a coordinated immunosuppressive transcriptional state rather than isolated checkpoint activation. Single-cell communication analyses in both cohorts consistently identified tumor epithelium–to–myeloid signaling dominated by CXCL8–CXCR1/2, IL6–IL6R, and CCL2–CCR2 axes, which were concordantly associated with GC-MIS levels, supporting a coupling between tumor-derived secreted signals and the myeloid suppressive niche [28].

In vitro experiments further supported tumor-cell signaling plasticity, showing that GC cells upregulated PD-L1 and activated STAT3 signaling in response to IFN-γ, IL-6, or OSM, and increased IL-6, IL-8, CCL2, and PTGS2 expression under inflammatory or hypoxic stimulation, effects that ruxolitinib, Stattic, or celecoxib attenuated. Collectively, these findings indicate that GC cells dynamically activate STAT3-, COX-2–, and chemokine-associated programs under inflammatory stress, which may contribute to a permissive environment for myeloid recruitment and immunosuppression, although direct causality toward MDSC functional programming was not demonstrated in this study [29, 30].

Consistent with prior reports on IL6/JAK–STAT3 signaling, chemokine-mediated myeloid recruitment, hypoxia-driven angiogenesis, and immune checkpoint pathways [31, 32]. However, they often lack a continuous chain from evidence of single-cell communication to cross-cohort prognostic associations, and then to tumor-side induction–inhibition validation [33]. Using GC-MIS as a stratification indicator can provide a clearer, interpretable target-combination hypothesis for subsequent exploration of combination strategies, without implying or demonstrating predictive value for immunotherapy response, as no immunotherapy-treated gastric cancer cohort was included in this study. 

### Limitations

Several limitations should be acknowledged. First, all transcriptomic cohorts were retrospective public datasets, and prospective validation is required before clinical implementation. Second, no immunotherapy-treated gastric cancer cohort was available; therefore, the potential predictive value of GC-MIS for response to immune checkpoint blockade remains speculative and cannot be evaluated in this study. Third, CellChat analyses provide transcriptome-based inference of potential ligand–receptor interactions and do not demonstrate direct signaling activity. Fourth, and most importantly regarding mechanistic interpretation, the in vitro experimental support is limited to tumor-cell signaling plasticity. We used only tumor cell lines (AGS, MKN45, HGC-27) and did not include any myeloid cells, co-culture systems, chemotaxis assays, migration assays, T-cell suppression assays, or direct MDSC functional validation. The experiments demonstrate that GC cells respond to inflammatory cytokines (IFN-γ, IL-6, OSM, TNF-α, IL-1β) and hypoxia-mimicking conditions (CoCl₂) with changes in PD-L1 expression, p-STAT3 signaling, and secretion of IL-6, IL-8, and CCL2, and that these effects are partially reversible by JAK/STAT or COX-2 inhibitors. However, these findings do not demonstrate that tumor cells can induce suppressive phenotypes in myeloid cells, nor do they prove that the identified pathways drive MDSC function or T-cell exhaustion in vivo. The interpretation is therefore restricted to tumor-cell signaling plasticity. Future studies incorporating co-culture of tumor cells with primary monocytes or neutrophils, chemotaxis assays, and T-cell suppression readouts are required to establish causal links between tumor-side signals and myeloid suppressive function. Fifth, spatial validation through spatial transcriptomics or multiplex immunohistochemistry was not available and warrants future investigation. Sixth, GC-MIS was not benchmarked against established immune-related signatures (e.g., TIDE, cytolytic activity, IFNγ, MDSC, CAF, or immune exclusion signatures); therefore, its relative prognostic performance relative to these frameworks remains undetermined. Finally, a potential limitation of GC-MIS is that the TU-module was partially defined based on its association with the MIS-module, resulting in shared variance between the two components. Therefore, the TU-module should not be interpreted as an entirely independent signature but rather as a tumor-derived transcriptional program linked to myeloid suppressive activity. This design reflects the biological objective of capturing tumor–myeloid coupling rather than constructing fully orthogonal features. Further studies incorporating independent tumor-derived feature selection or functional validation will further strengthen this framework.

## Conclusions

GC-MIS integrates single-cell-derived suppressive myeloid programs with tumor-associated induction signals, providing a reproducible framework for quantifying a myeloid-associated immunosuppressive niche across bulk transcriptomic cohorts. High GC-MIS is accompanied by myeloid enrichment, enhanced T-cell exhaustion, and reduced antigen presentation, and remains independently associated after controlling for tumor purity and stage. The GC-MIS–based research risk model maintained discrimination and calibration in external cohorts and provided net benefit within the mid-to-high threshold probability range. Single-cell communication analyses and in vitro experiments provide supportive evidence that tumor-associated inflammatory and hypoxic signaling programs may contribute to the establishment of suppressive myeloid-associated microenvironmental states. GC-MIS can serve as a stratification indicator to explore combination strategies for immunotherapy with anti-inflammatory, anti-chemotaxis, anti-STAT3, or anti-hypoxia approaches.

## Supplementary Information

Supplementary Material 1.

Supplementary Material 2.

## Data Availability

All datasets analyzed during this study are publicly available: GSE206785, GSE183904, GSE62254, and GSE84437 from the GEO database, and TCGA-STAD from the TCGA database. No additional datasets were generated.

## Abbreviations

CV: Coefficient of Variation
GC: Gastric Cancer
GC-MIS: Gastric Cancer Myeloid Immunosuppressive Niche Score
MDSC: Myeloid-Derived Suppressor Cell
MIS: Myeloid Immunosuppressive Niche Signature
OS: Overall Survival
TCGA: The Cancer Genome Atlas
