# Supplementary material for: Single-cell and bulk transcriptome-based identification of myeloid-derived suppressor cell-associated immune evasion signatures and a prognostic model for gastric cancer
Source: BMC Cancer. 2026 Jul 29;26:1298. doi: 10.1186/s12885-026-16584-0 (PMC13700161; doi:10.1186/s12885-026-16584-0)
Supplement: Supplementary file 1 [file 12885_2026_16584_MOESM2_ESM.pdf]

Full-Length Western Blot Images

Supporting Figure 5A

PD-L1 and p-STAT3 induction and inhibition in gastric cancer cell lines

AGS | MKN45 | HGC-27

PD-L1 | beta-actin | p-STAT3 (Tyr705) | total STAT3

36 full-membrane images | 3 biological replicates | 7 treatment conditions

Contents and lane order

Cell line	Replicate	Status	Pages
AGS	Replicate 1	MANUSCRIPT	3-6
AGS	Replicate 2		7-10
AGS	Replicate 3		11-14
MKN45	Replicate 1	MANUSCRIPT	15-18
MKN45	Replicate 2		19-22
MKN45	Replicate 3		23-26
HGC-27	Replicate 1	MANUSCRIPT	27-30
HGC-27	Replicate 2		31-34
HGC-27	Replicate 3		35-38

Lane order (left to right)

1	Control
2	IFN-gamma
3	IFN-gamma + Ruxo
4	IL-6
5	IL-6 + Stattic
6	OSM
7	OSM + Stattic

AGS | Biological replicate 1 | PD-L1

This original blot corresponds to the cropped blot: AGS_Rep1_WesternBlot

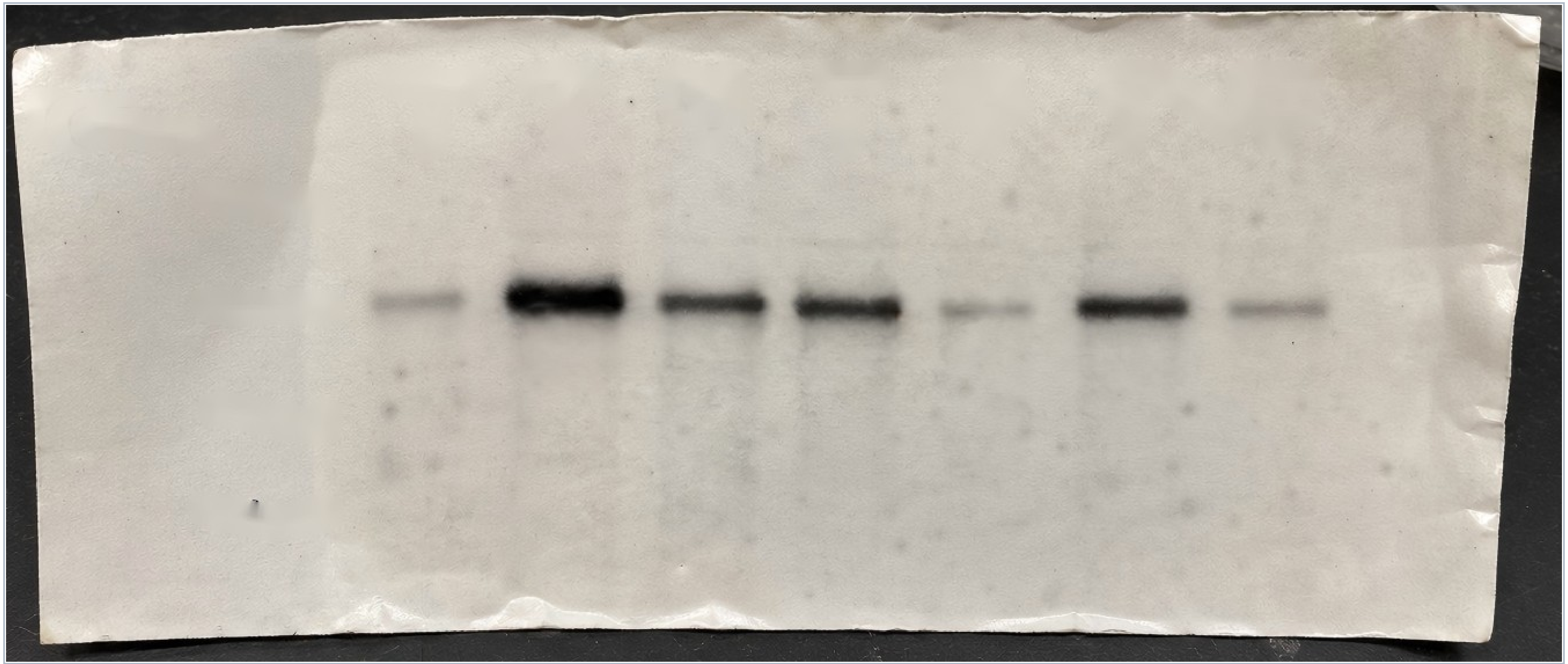

1	2	3	4	5	6	7
Control	IFN-gamma	IFN-gamma + Ruxo	IL-6	IL-6 + Stattic	OSM	OSM + Stattic

AGS | Biological replicate 1 | beta-actin

This original blot corresponds to the cropped blot: AGS_Rep1_WesternBlot

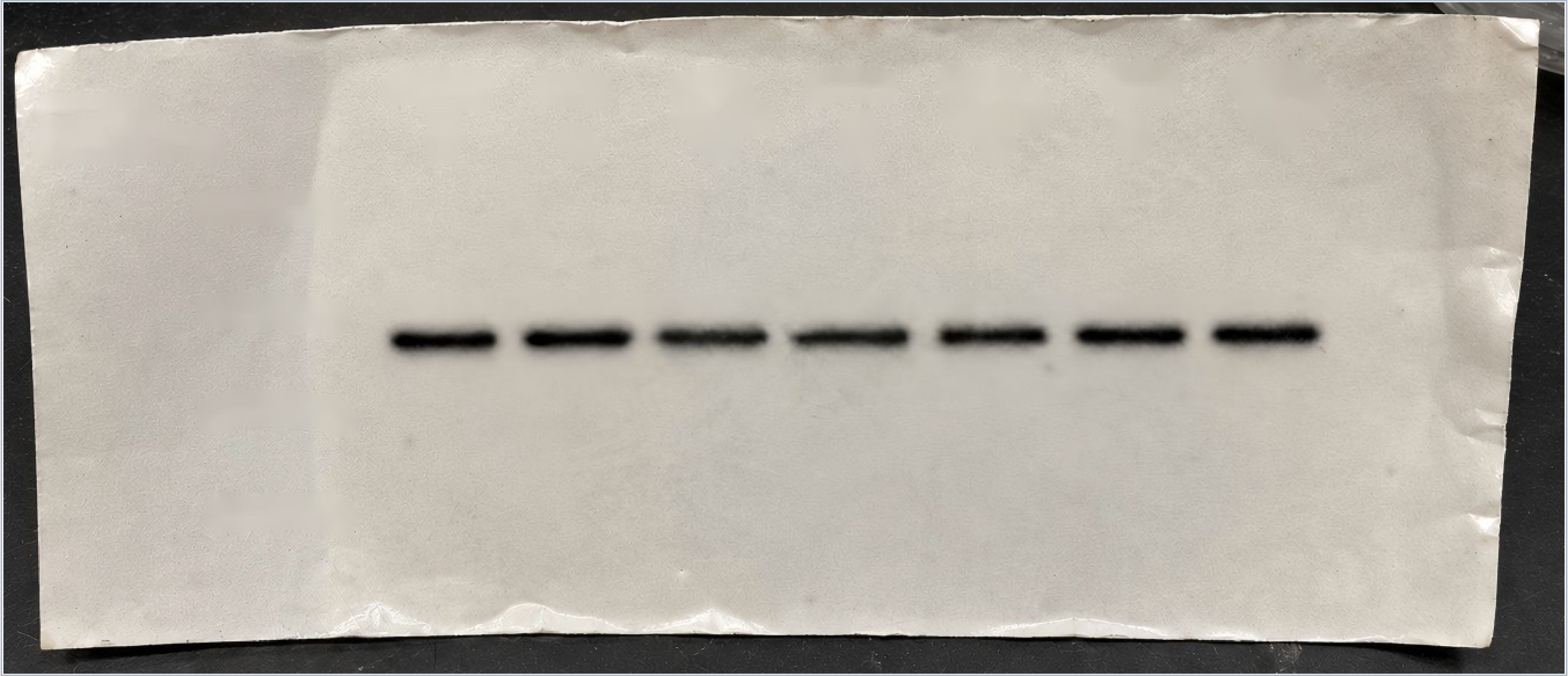

1	2	3	4	5	6	7
Control	IFN-gamma	IFN-gamma + Ruxo	IL-6	IL-6 + Stattic	OSM	OSM + Stattic

AGS | Biological replicate 1 | p-STAT3 (Tyr705)

This original blot corresponds to the cropped blot: AGS_Rep1_WesternBlot

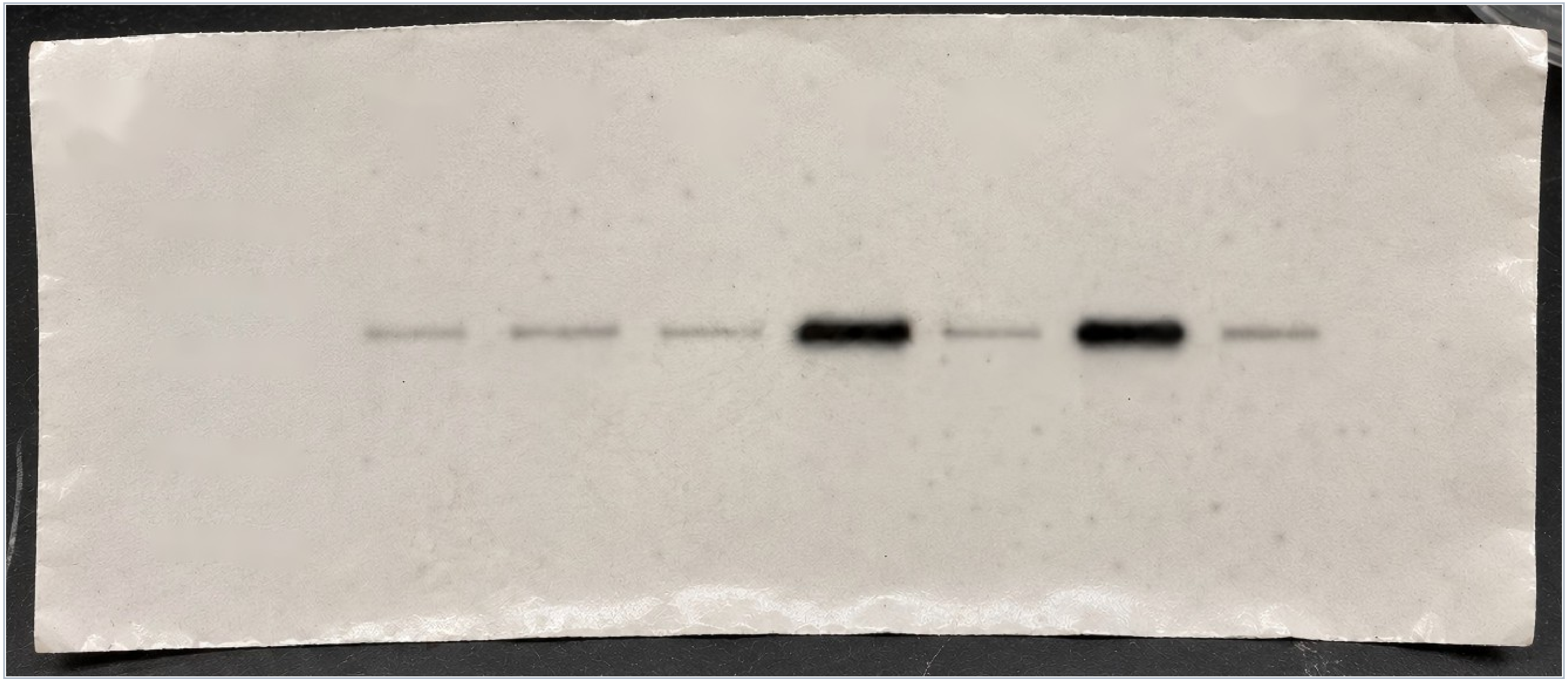

1	2	3	4	5	6	7
Control	IFN-gamma	IFN-gamma + Ruxo	IL-6	IL-6 + Stattic	OSM	OSM + Stattic

AGS | Biological replicate 1 | total STAT3

This original blot corresponds to the cropped blot: AGS_Rep1_WesternBlot

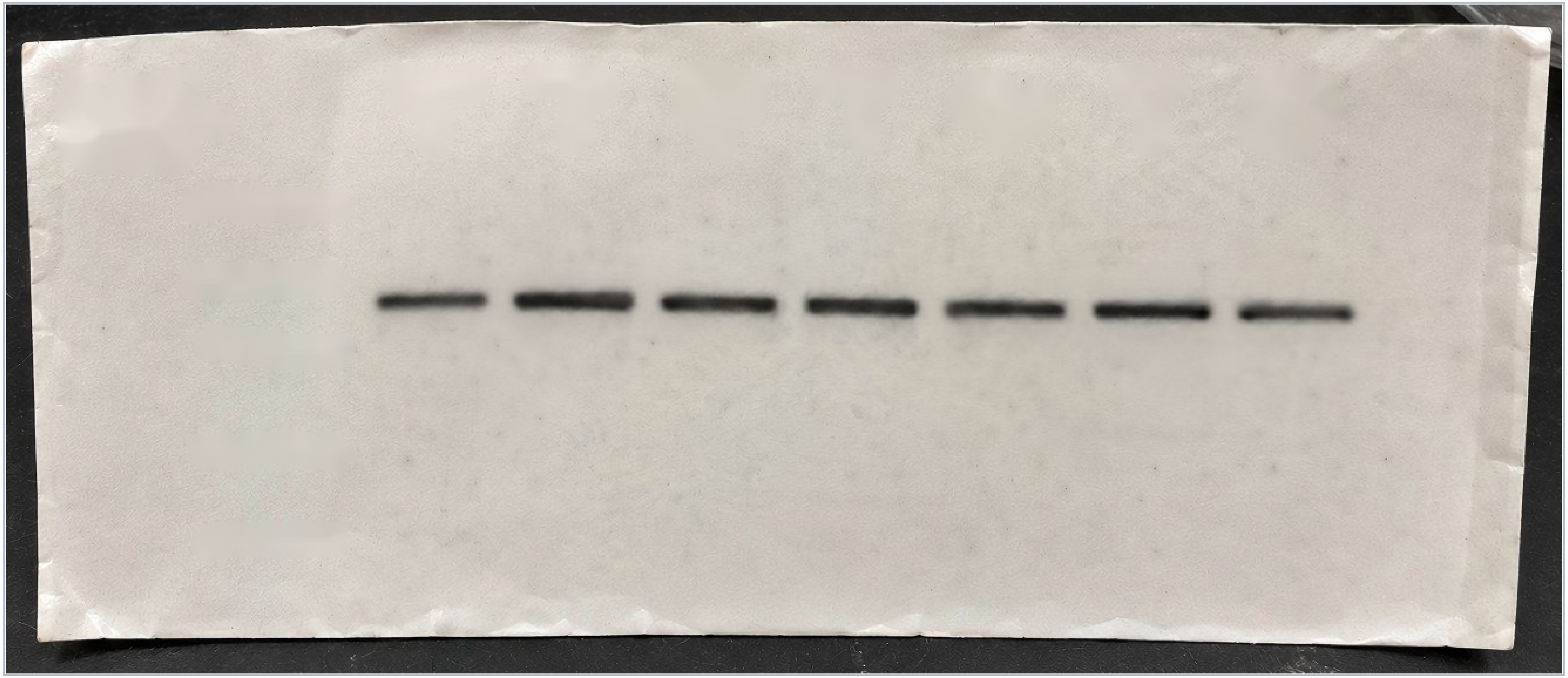

1	2	3	4	5	6	7
Control	IFN-gamma	IFN-gamma + Ruxo	IL-6	IL-6 + Stattic	OSM	OSM + Stattic

AGS | Biological replicate 2 | PD-L1

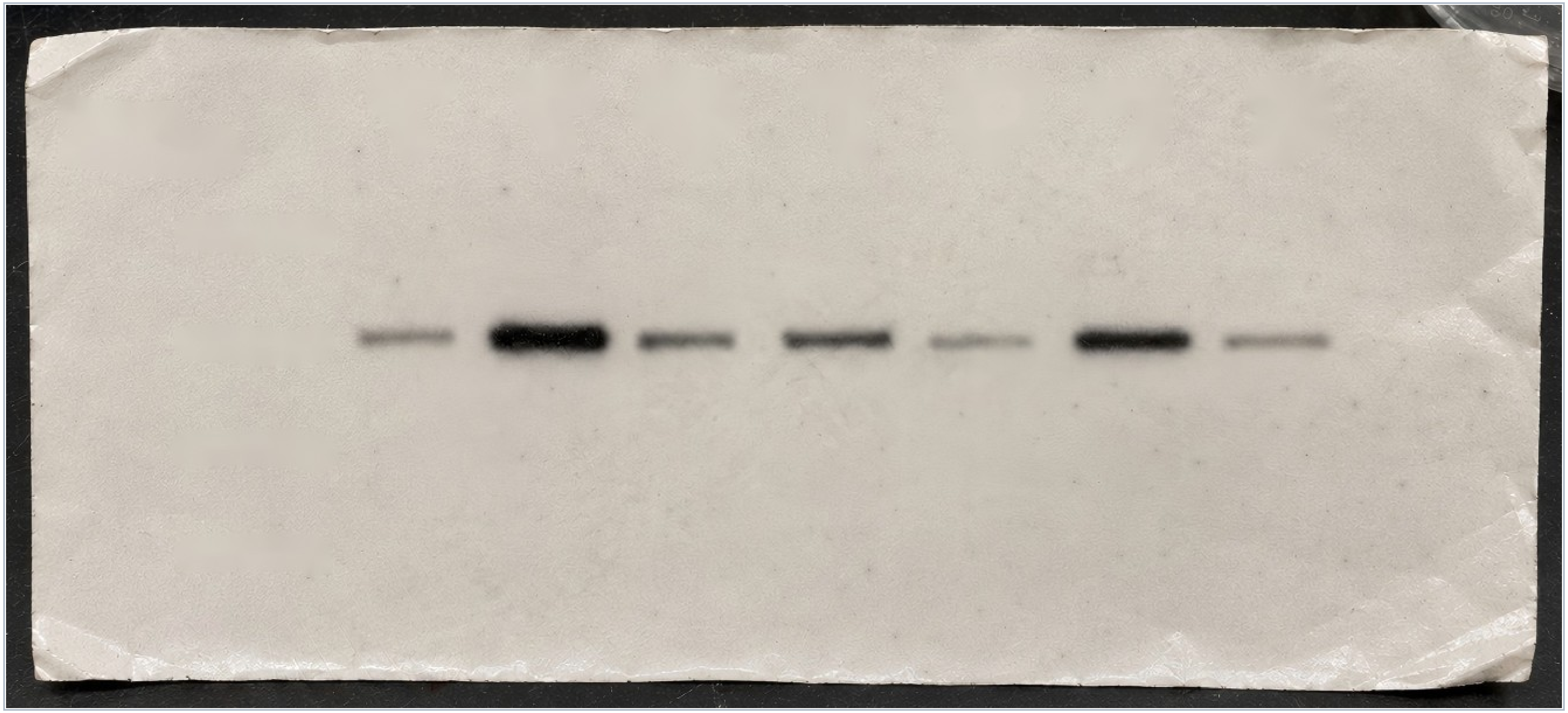

1	2	3	4	5	6	7
Control	IFN-gamma	IFN-gamma + Ruxo	IL-6	IL-6 + Stattic	OSM	OSM + Stattic

AGS | Biological replicate 2 | beta-actin

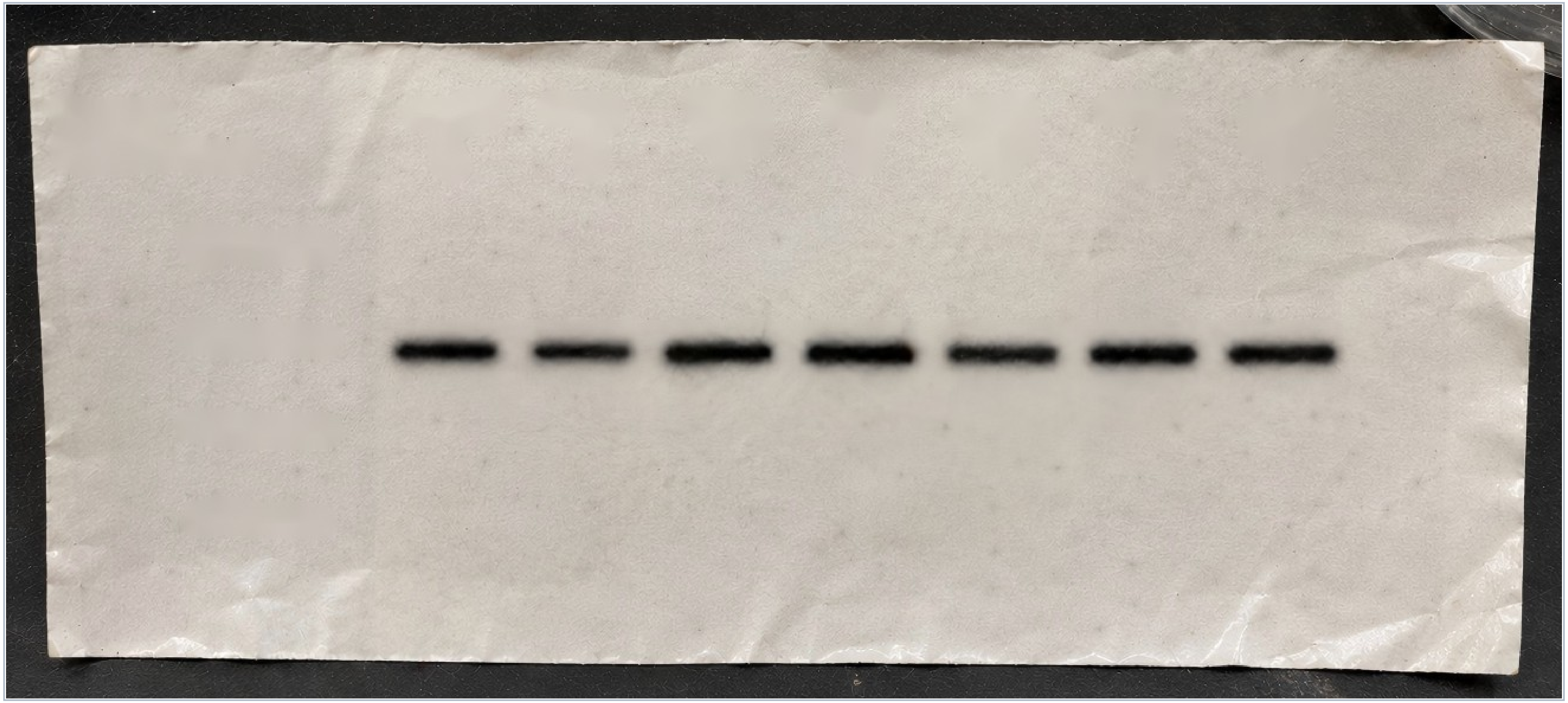

1	2	3	4	5	6	7
Control	IFN-gamma	IFN-gamma + Ruxo	IL-6	IL-6 + Stattic	OSM	OSM + Stattic

AGS | Biological replicate 2 | p-STAT3 (Tyr705)

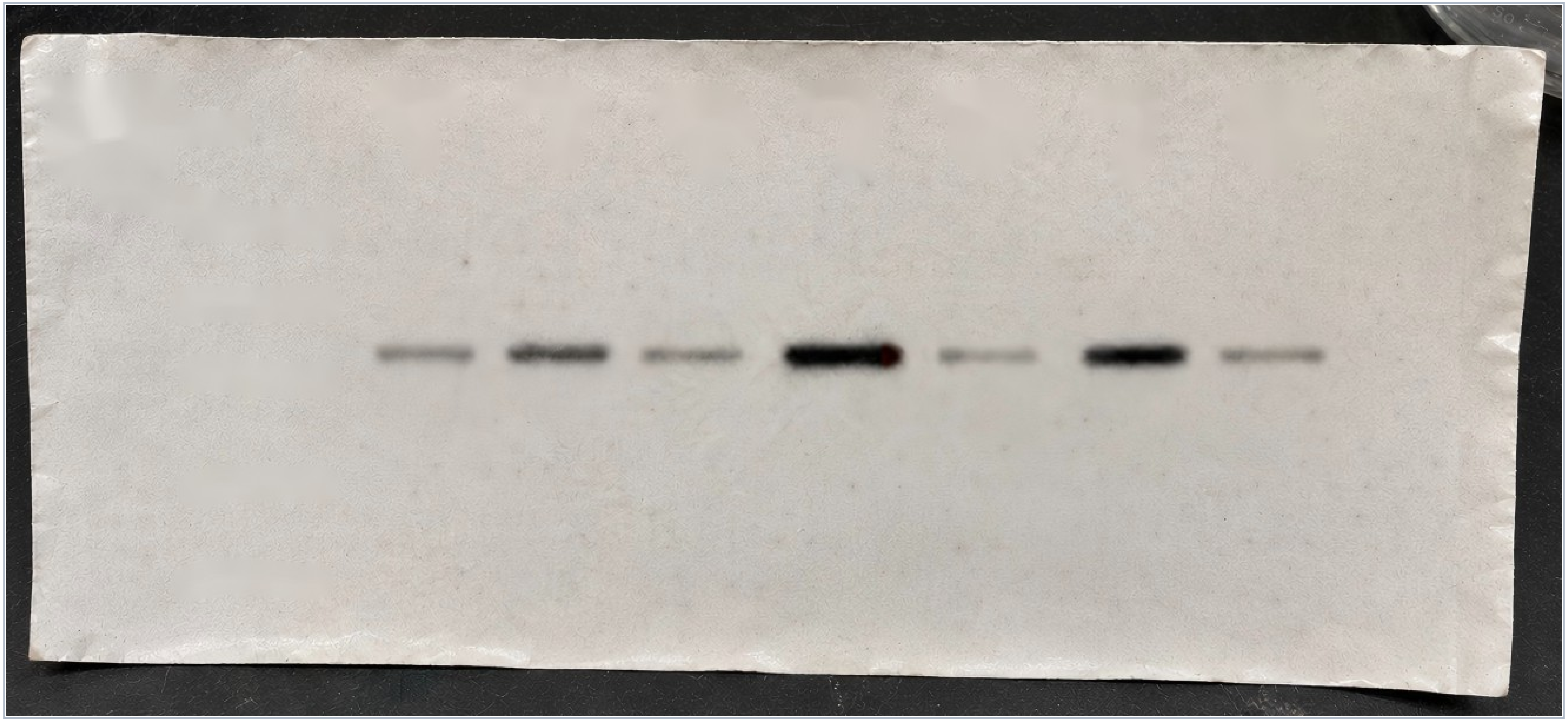

1	2	3	4	5	6	7
Control	IFN-gamma	IFN-gamma + Ruxo	IL-6	IL-6 + Stattic	OSM	OSM + Stattic

AGS | Biological replicate 2 | total STAT3

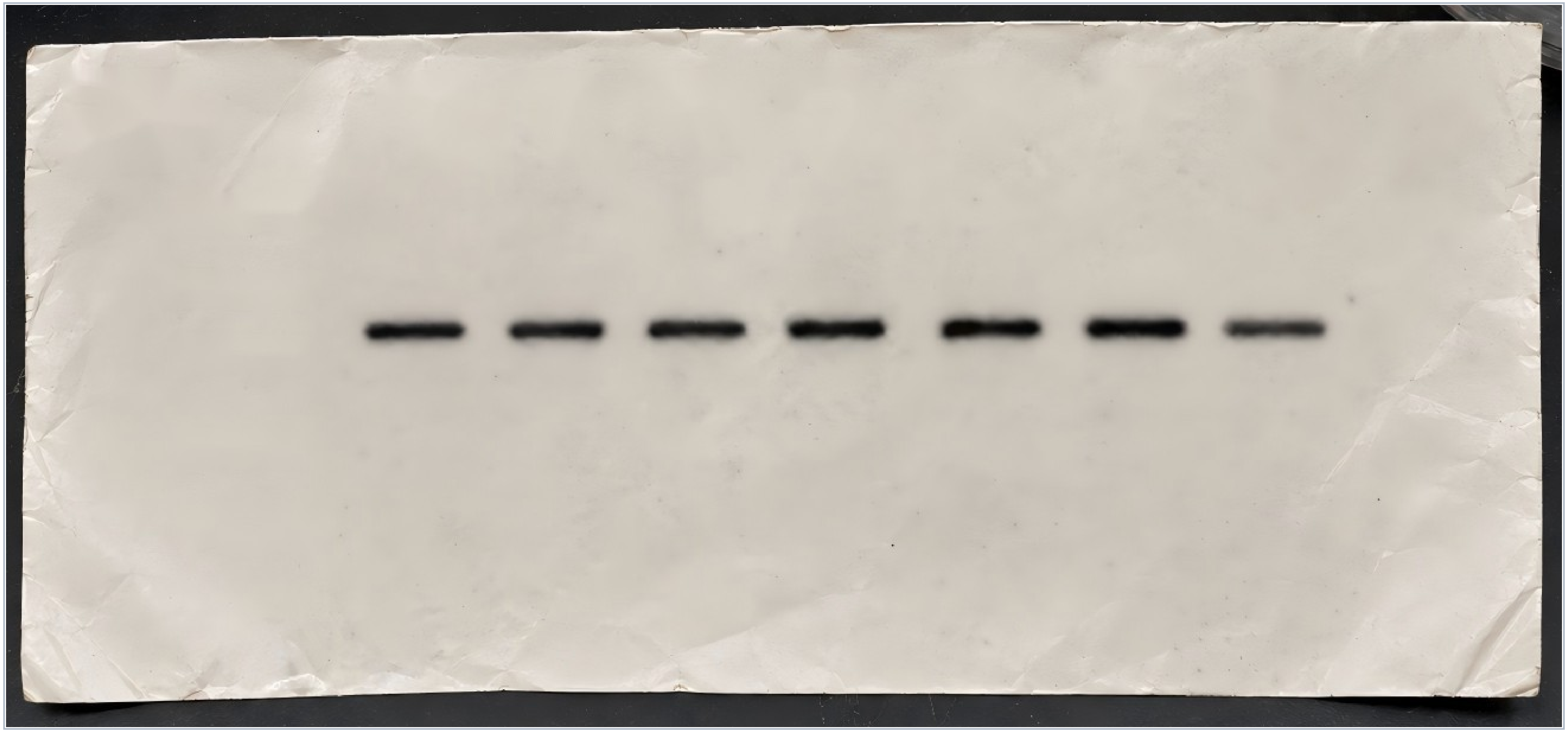

1	2	3	4	5	6	7
Control	IFN-gamma	IFN-gamma + Ruxo	IL-6	IL-6 + Stattic	OSM	OSM + Stattic

AGS | Biological replicate 3 | PD-L1

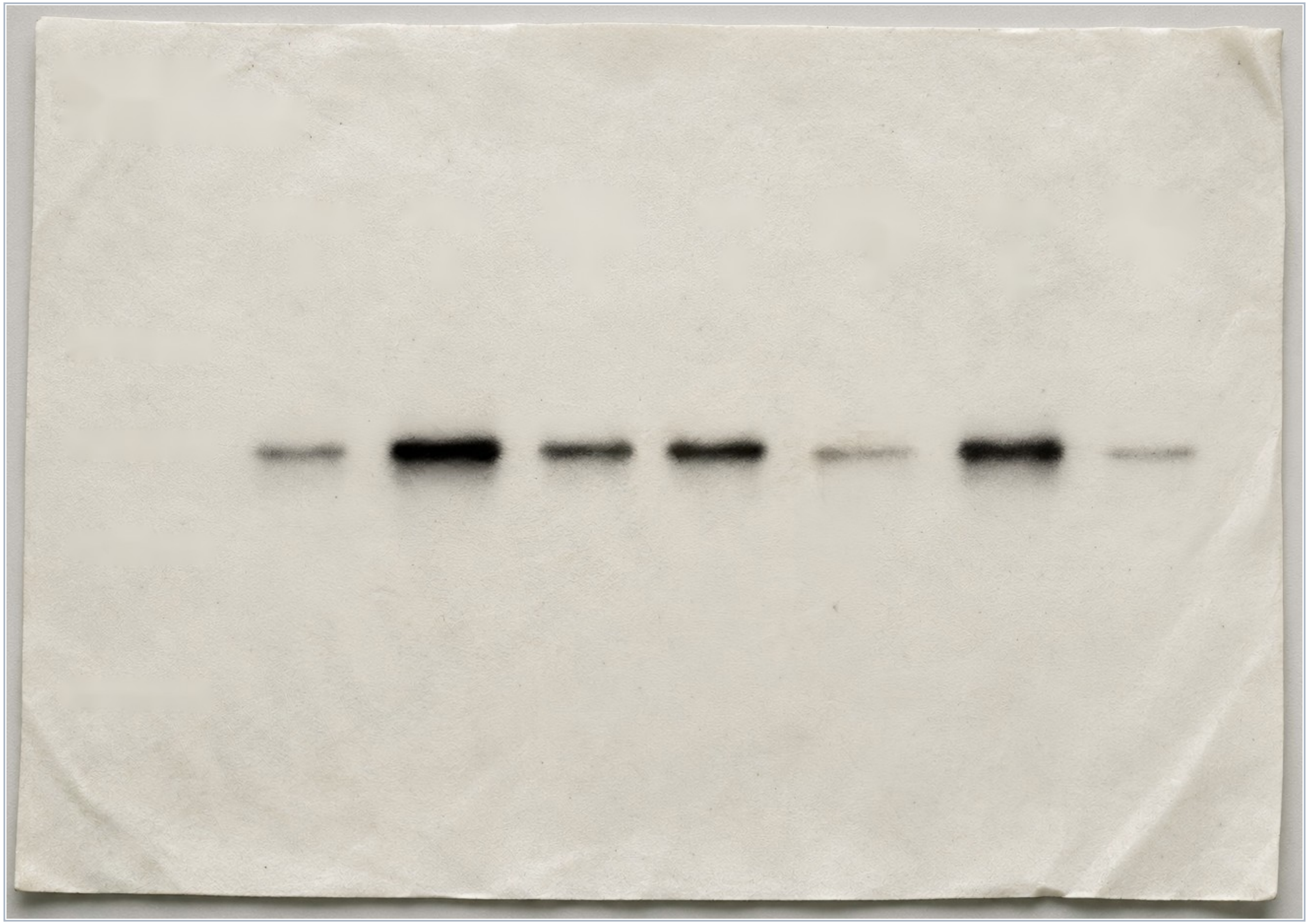

1	2	3	4	5	6	7
Control	IFN-gamma	IFN-gamma + Ruxo	IL-6	IL-6 + Stattic	OSM	OSM + Stattic

AGS | Biological replicate 3 | beta-actin

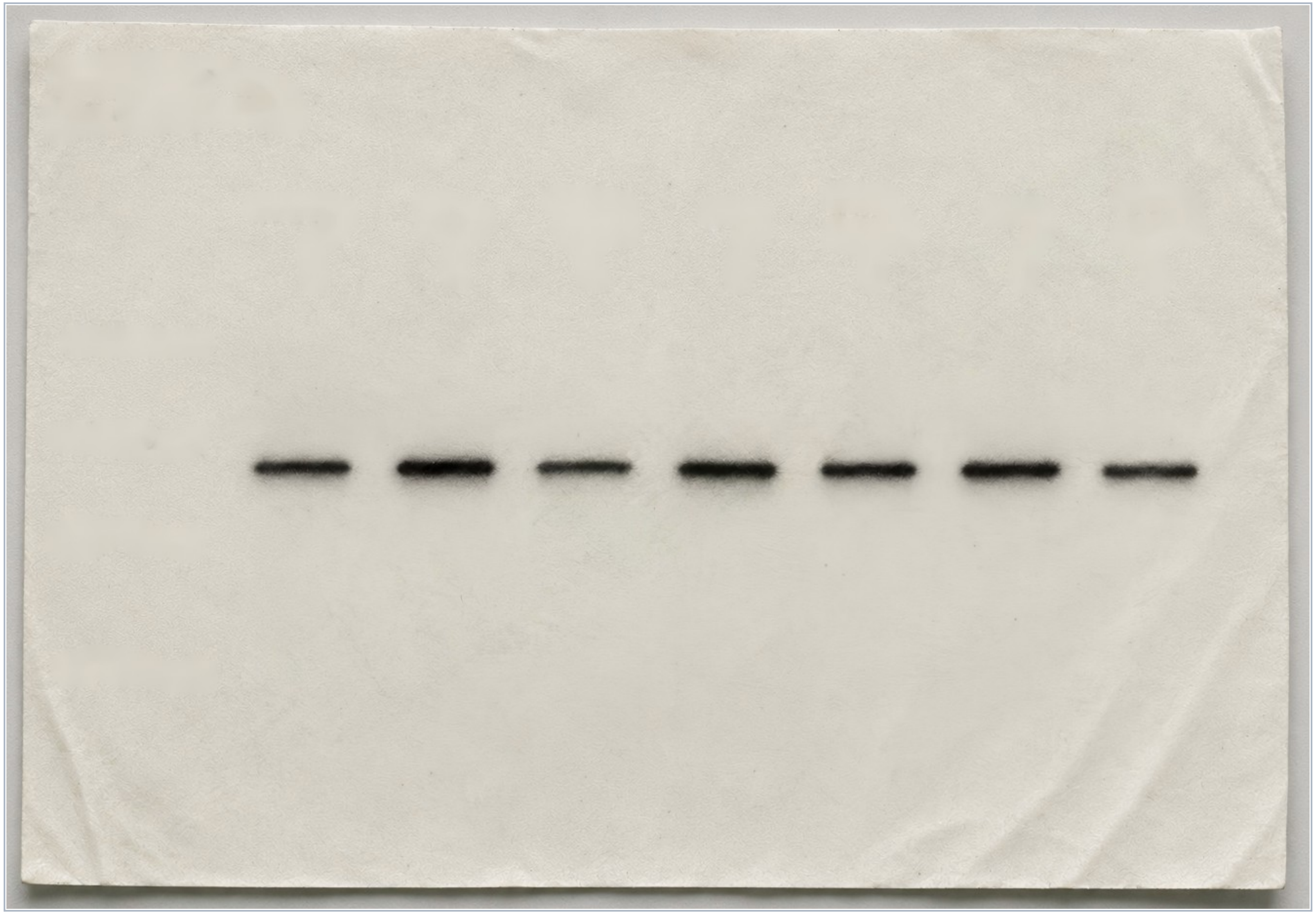

1	2	3	4	5	6	7
Control	IFN-gamma	IFN-gamma + Ruxo	IL-6	IL-6 + Stattic	OSM	OSM + Stattic

AGS | Biological replicate 3 | p-STAT3 (Tyr705)

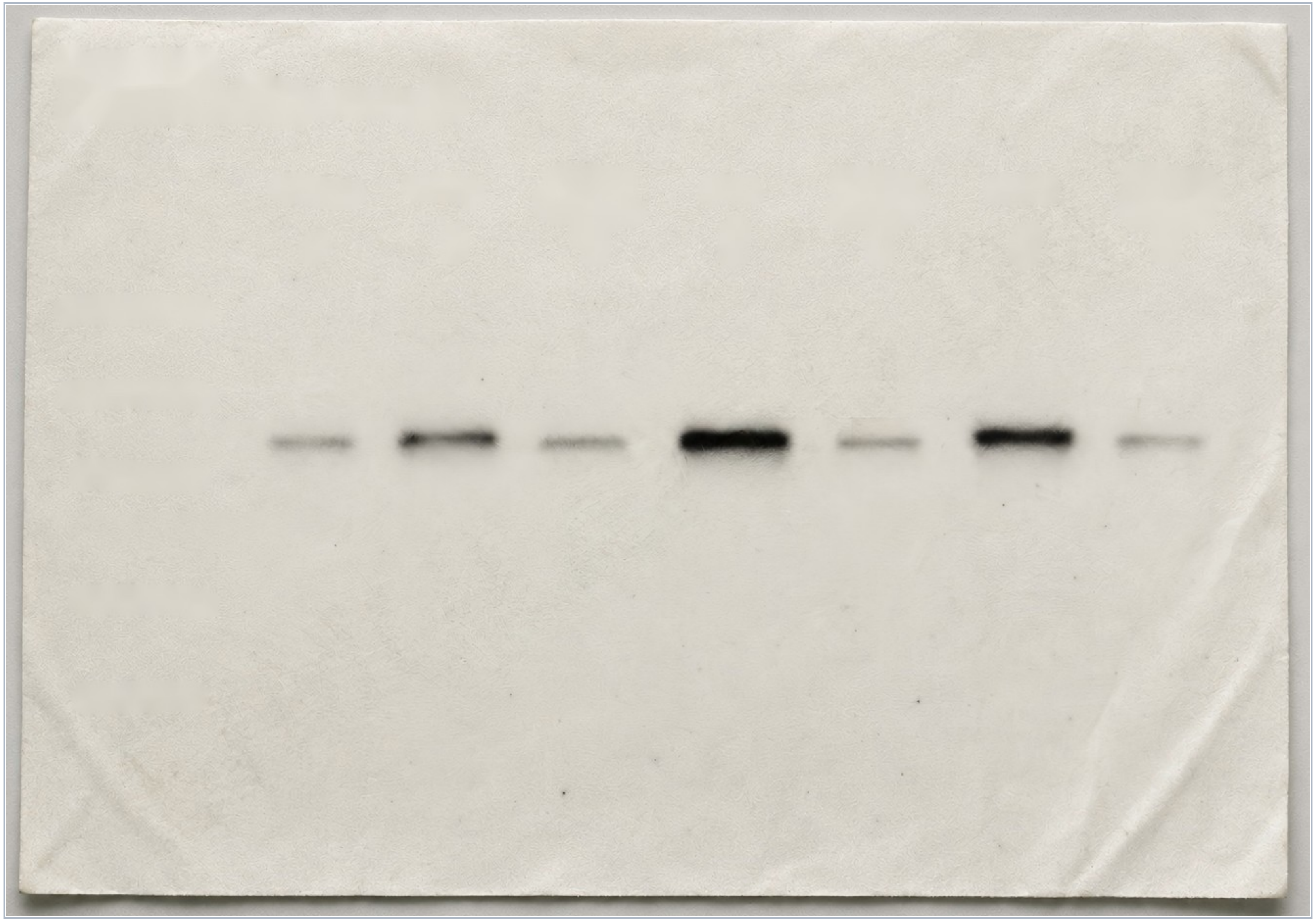

1	2	3	4	5	6	7
Control	IFN-gamma	IFN-gamma + Ruxo	IL-6	IL-6 + Stattic	OSM	OSM + Stattic

AGS | Biological replicate 3 | total STAT3

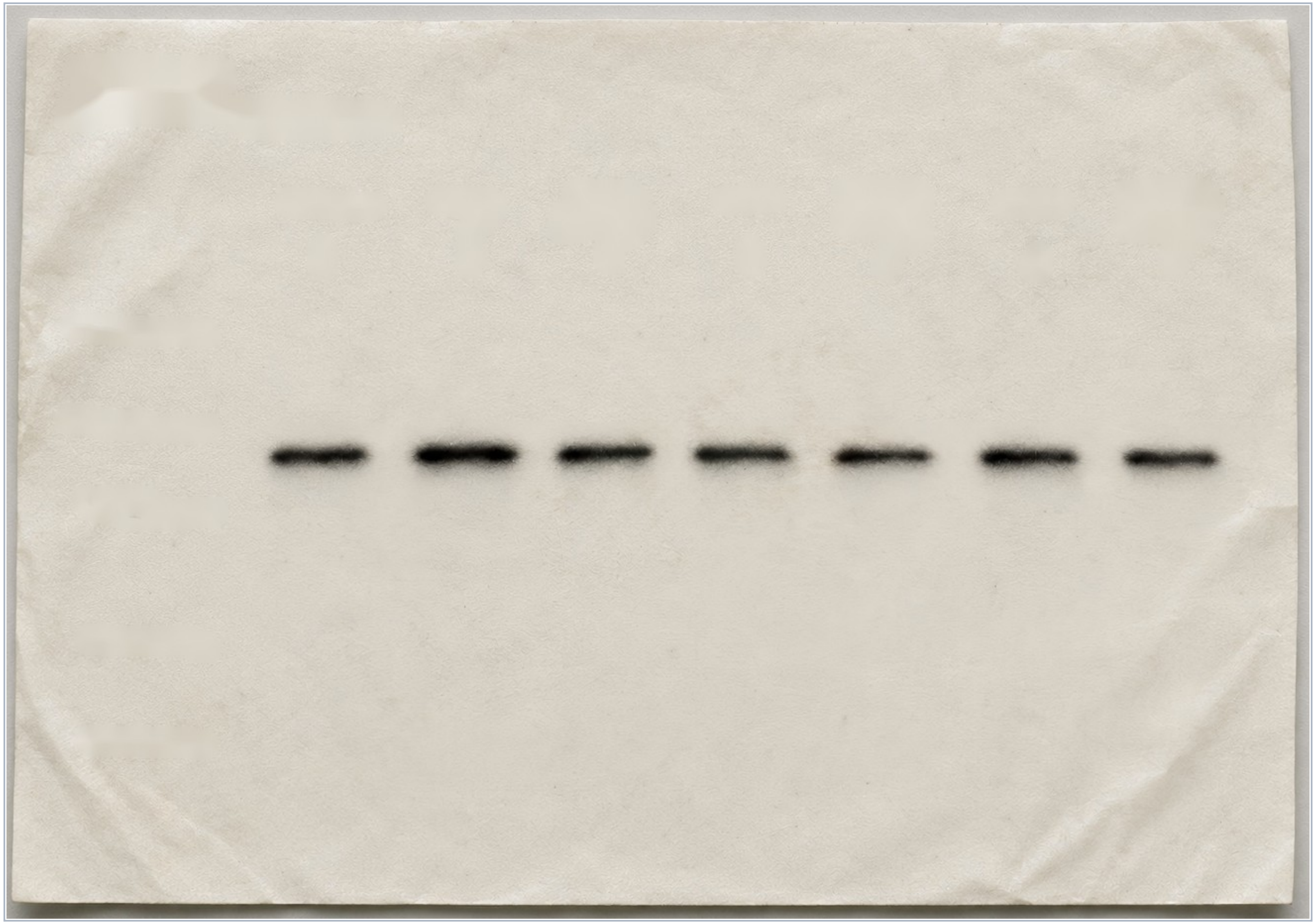

1	2	3	4	5	6	7
Control	IFN-gamma	IFN-gamma + Ruxo	IL-6	IL-6 + Stattic	OSM	OSM + Stattic

MKN45 | Biological replicate 1 | PD-L1

This original blot corresponds to the cropped blot: MKN45_Rep1_WesternBlot

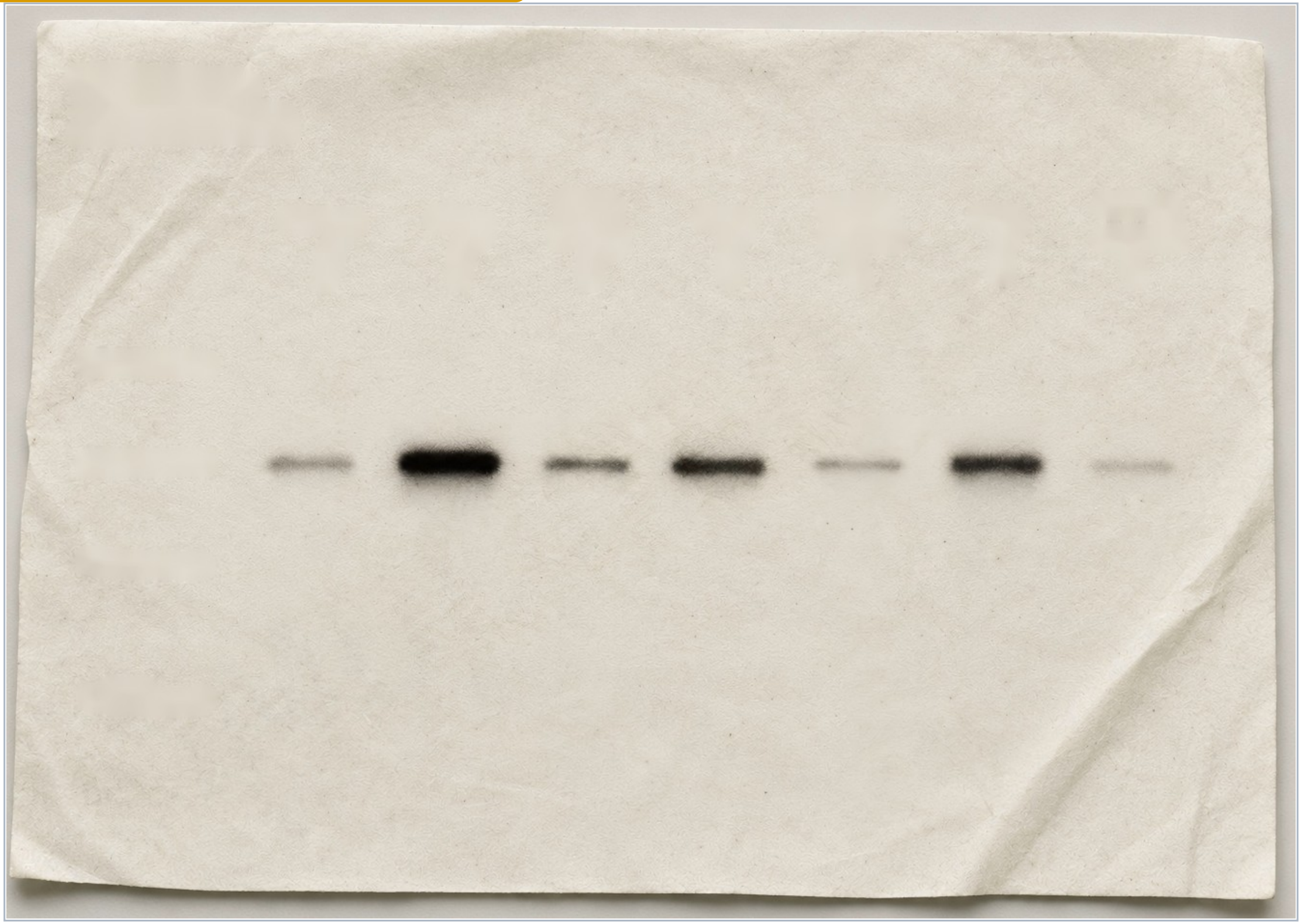

1	2	3	4	5	6	7
Control	IFN-gamma	IFN-gamma + Ruxo	IL-6	IL-6 + Stattic	OSM	OSM + Stattic

MKN45 | Biological replicate 1 | beta-actin

This original blot corresponds to the cropped blot: MKN45_Rep1_WesternBlot

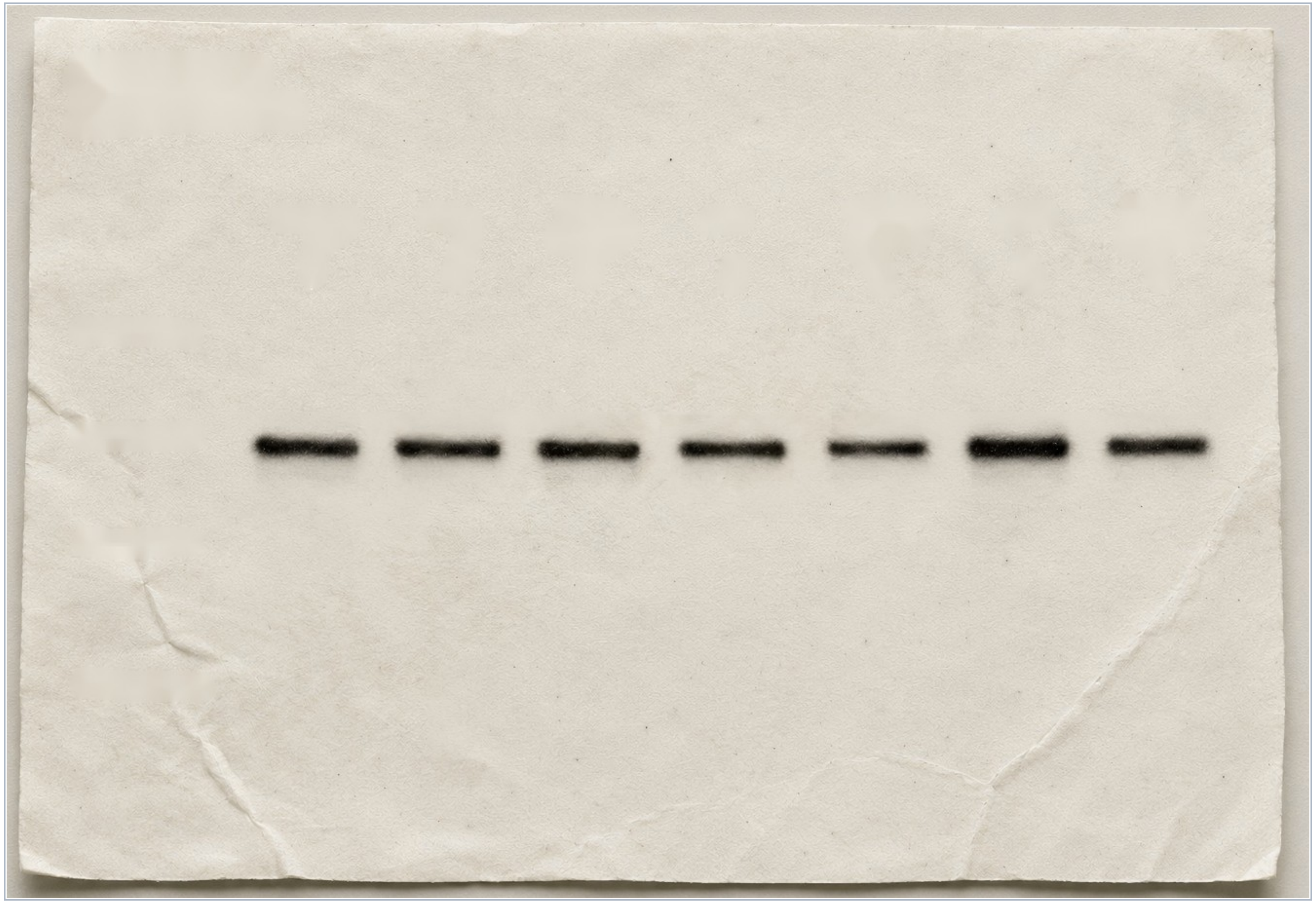

1	2	3	4	5	6	7
Control	IFN-gamma	IFN-gamma + Ruxo	IL-6	IL-6 + Stattic	OSM	OSM + Stattic

MKN45 | Biological replicate 1 | p-STAT3 (Tyr705)

This original blot corresponds to the cropped blot: MKN45_Rep1_WesternBlot

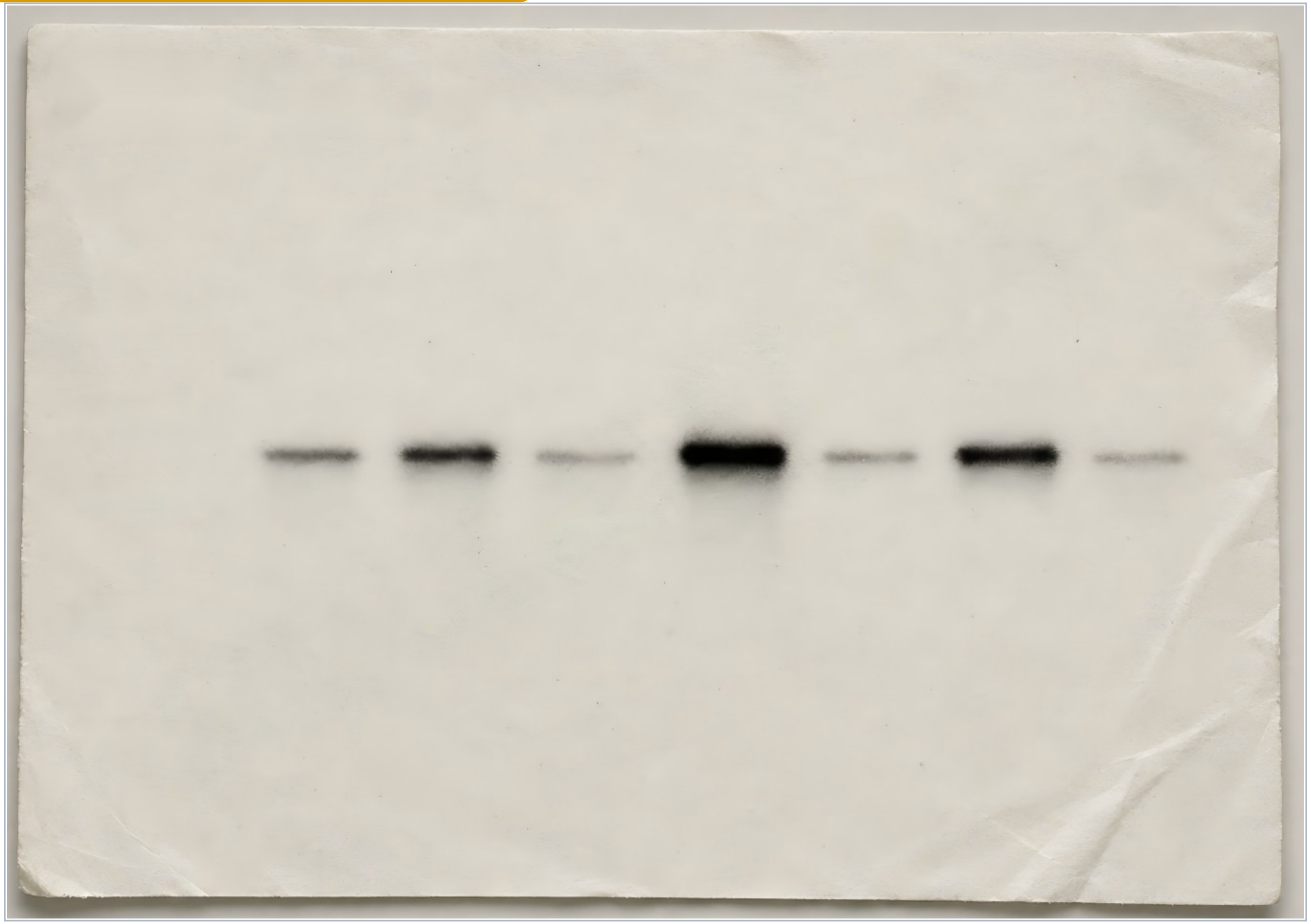

1	2	3	4	5	6	7
Control	IFN-gamma	IFN-gamma + Ruxo	IL-6	IL-6 + Stattic	OSM	OSM + Stattic

MKN45 | Biological replicate 1 | total STAT3

This original blot corresponds to the cropped blot: MKN45_Rep1_WesternBlot

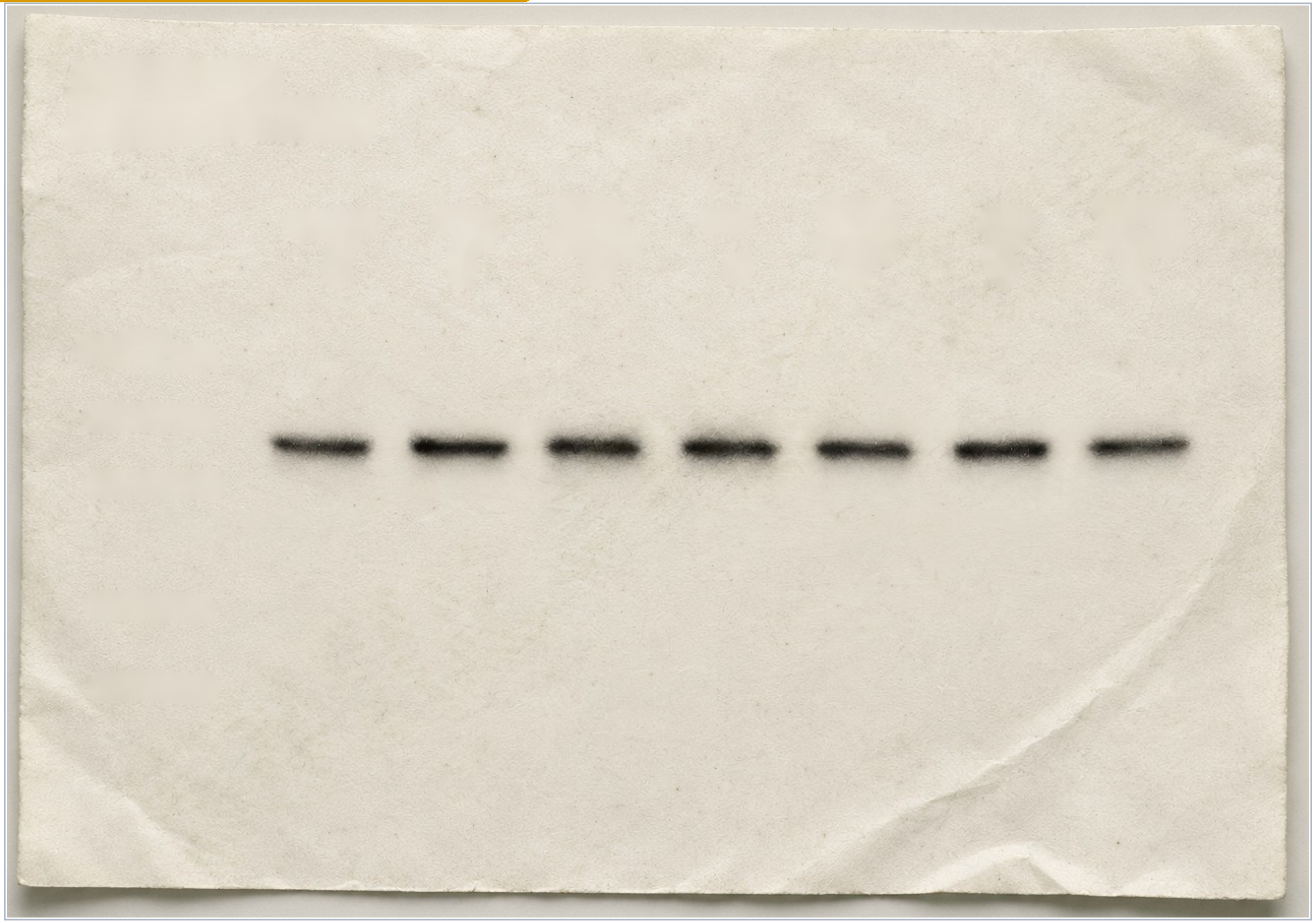

1	2	3	4	5	6	7
Control	IFN-gamma	IFN-gamma + Ruxo	IL-6	IL-6 + Stattic	OSM	OSM + Stattic

MKN45 | Biological replicate 2 | PD-L1

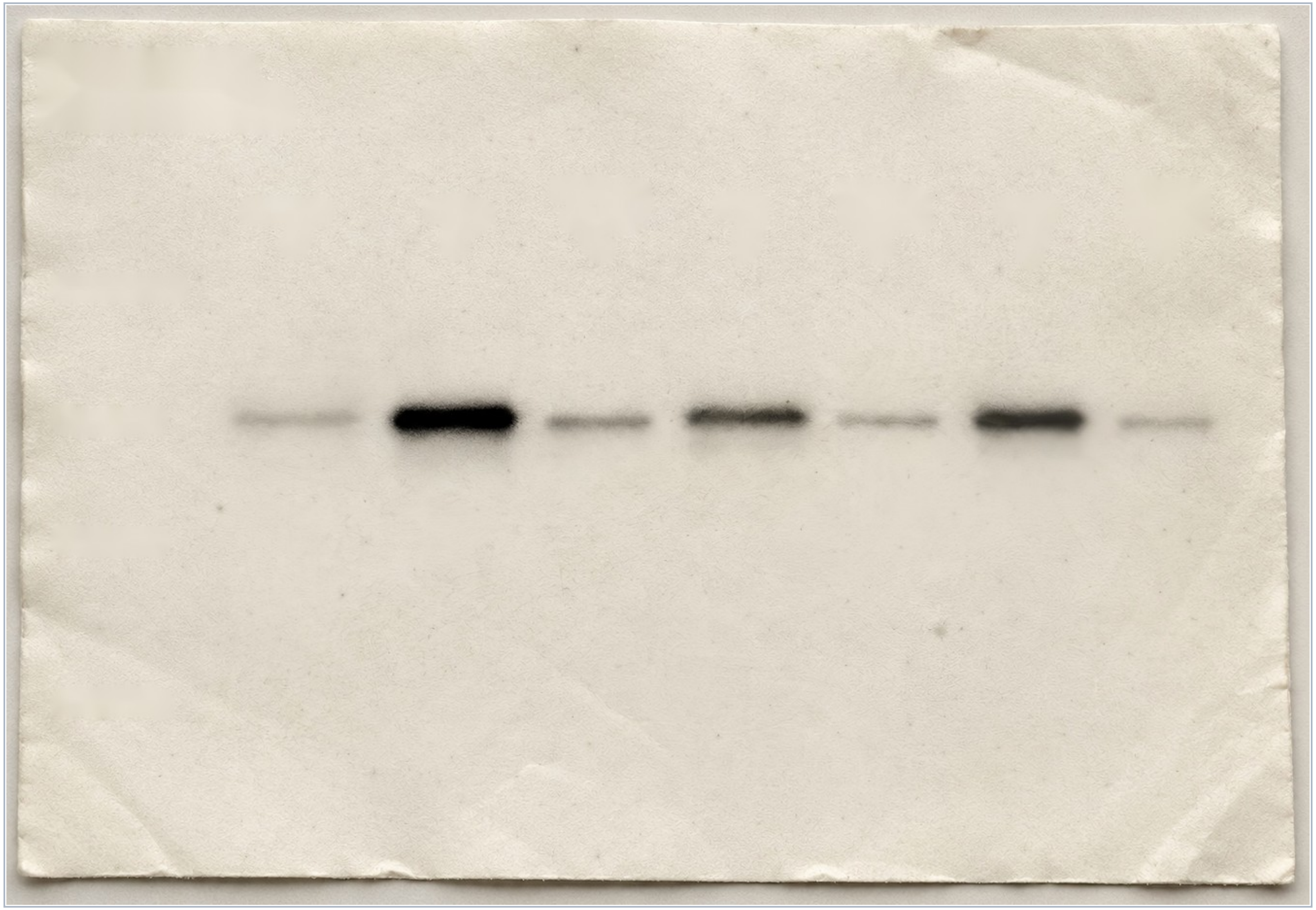

1	2	3	4	5	6	7
Control	IFN-gamma	IFN-gamma + Ruxo	IL-6	IL-6 + Stattic	OSM	OSM + Stattic

MKN45 | Biological replicate 2 | beta-actin

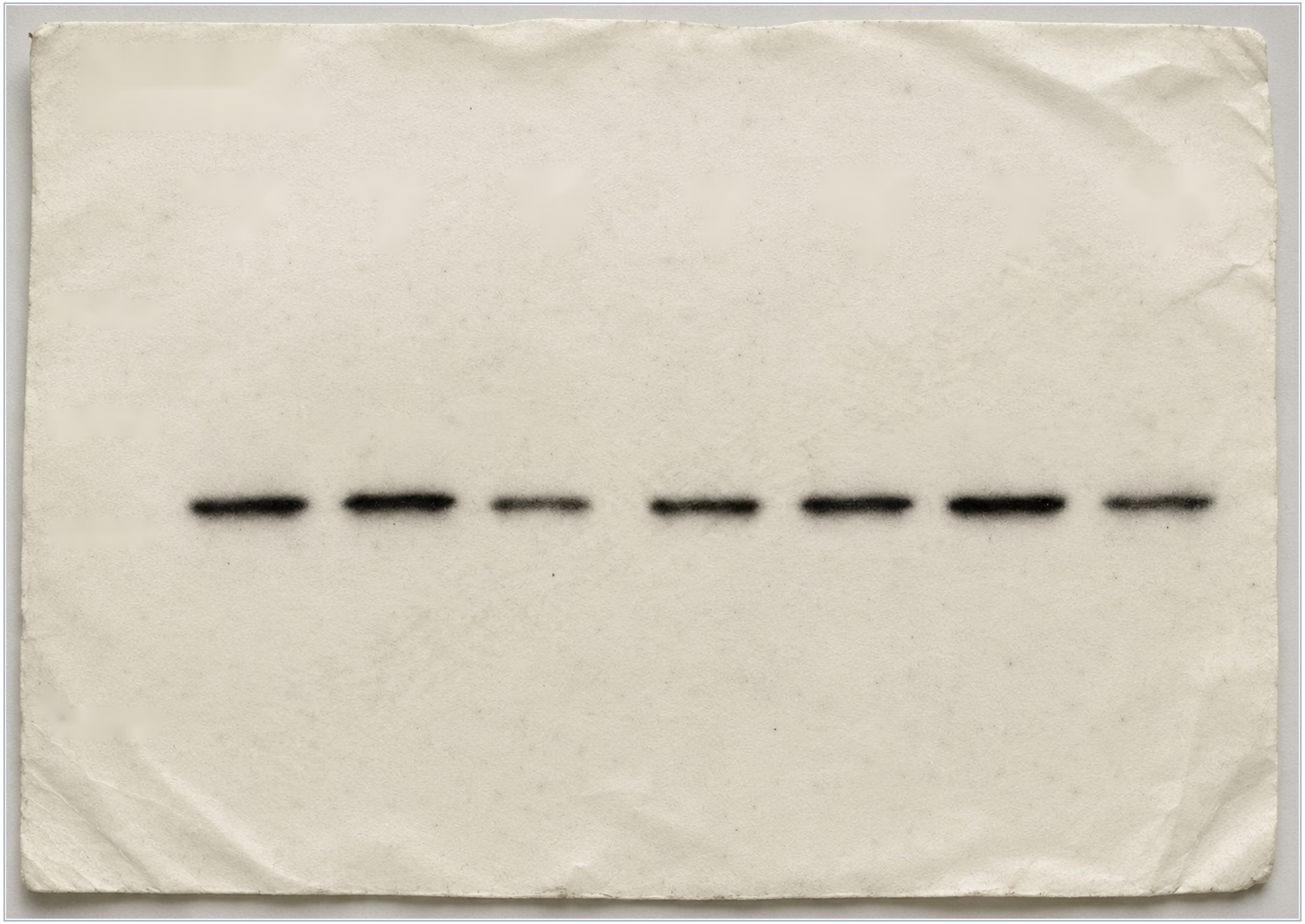

1	2	3	4	5	6	7
Control	IFN-gamma	IFN-gamma + Ruxo	IL-6	IL-6 + Stattic	OSM	OSM + Stattic

MKN45 | Biological replicate 2 | p-STAT3 (Tyr705)

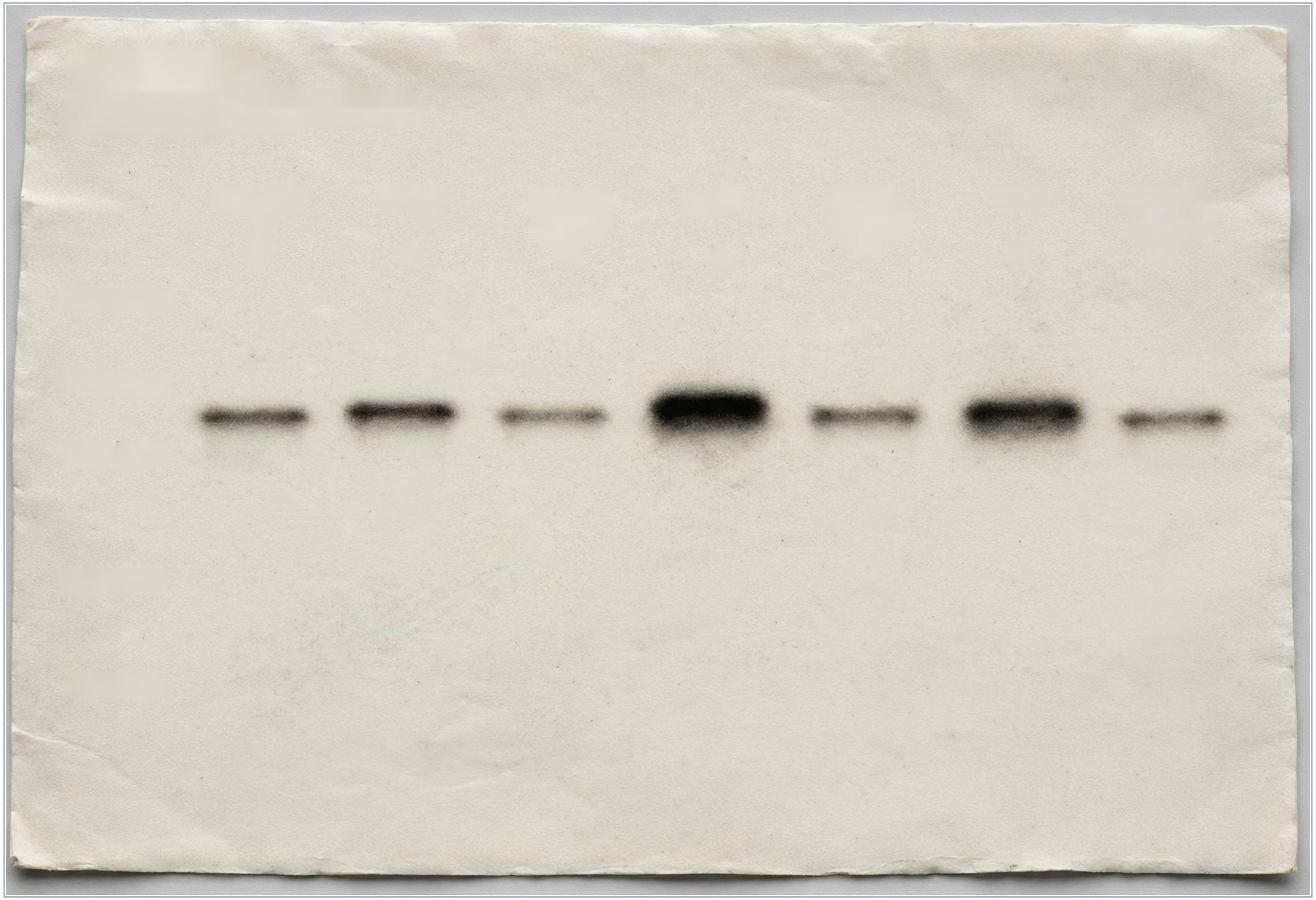

1	2	3	4	5	6	7
Control	IFN-gamma	IFN-gamma + Ruxo	IL-6	IL-6 + Stattic	OSM	OSM + Stattic

MKN45 | Biological replicate 2 | total STAT3

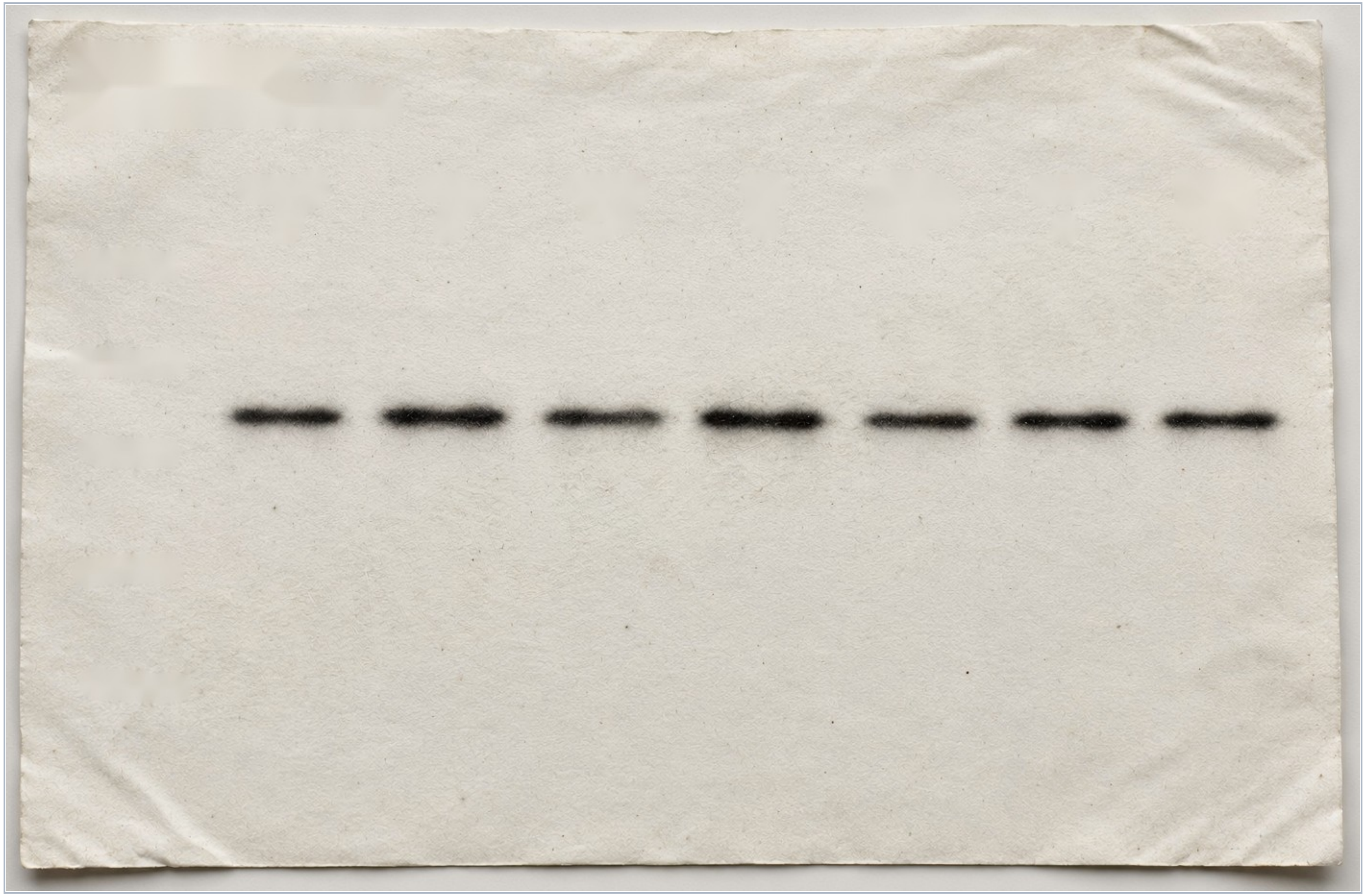

1	2	3	4	5	6	7
Control	IFN-gamma	IFN-gamma + Ruxo	IL-6	IL-6 + Stattic	OSM	OSM + Stattic

MKN45 | Biological replicate 3 | PD-L1

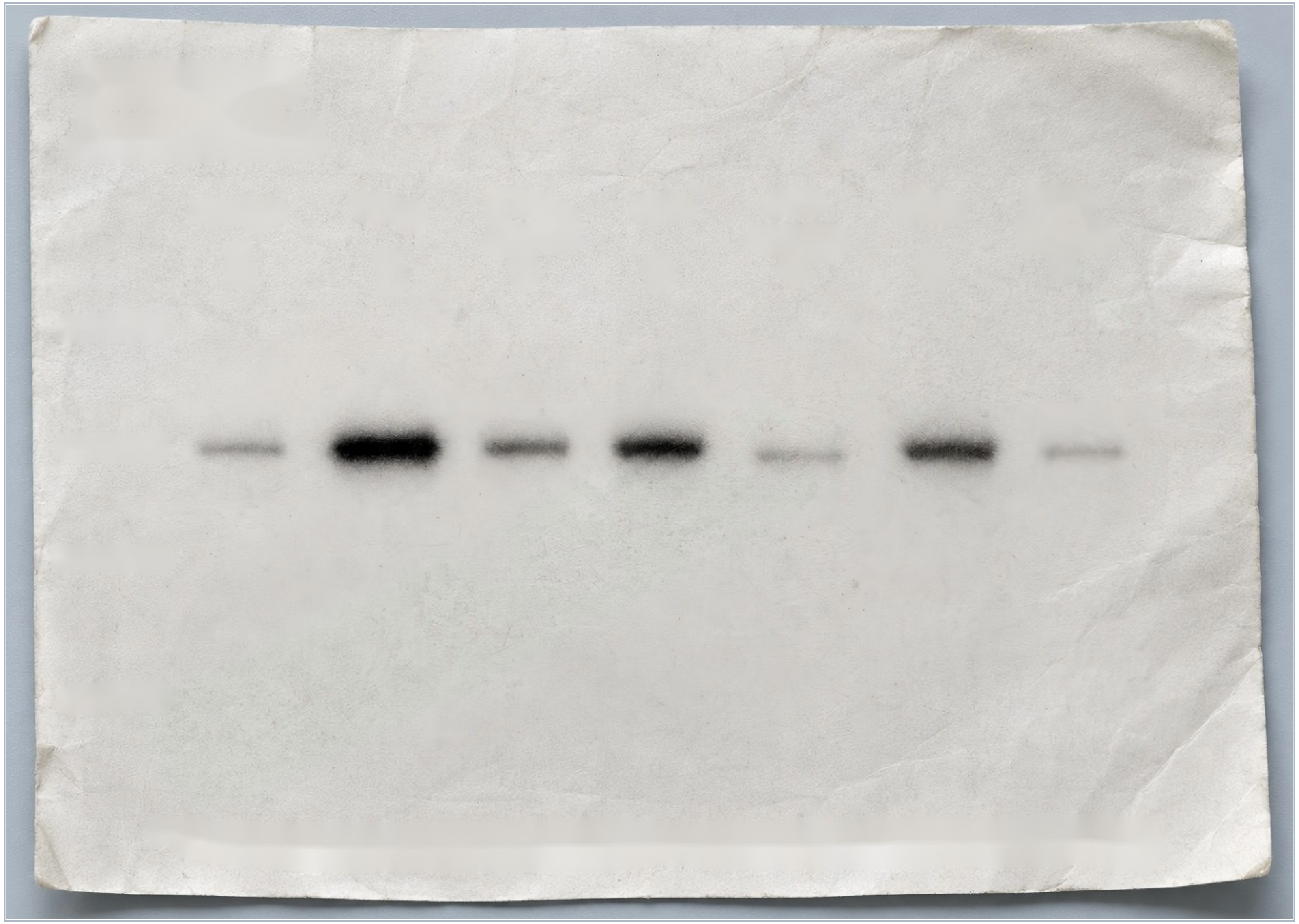

1	2	3	4	5	6	7
Control	IFN-gamma	IFN-gamma + Ruxo	IL-6	IL-6 + Stattic	OSM	OSM + Stattic

MKN45 | Biological replicate 3 | beta-actin

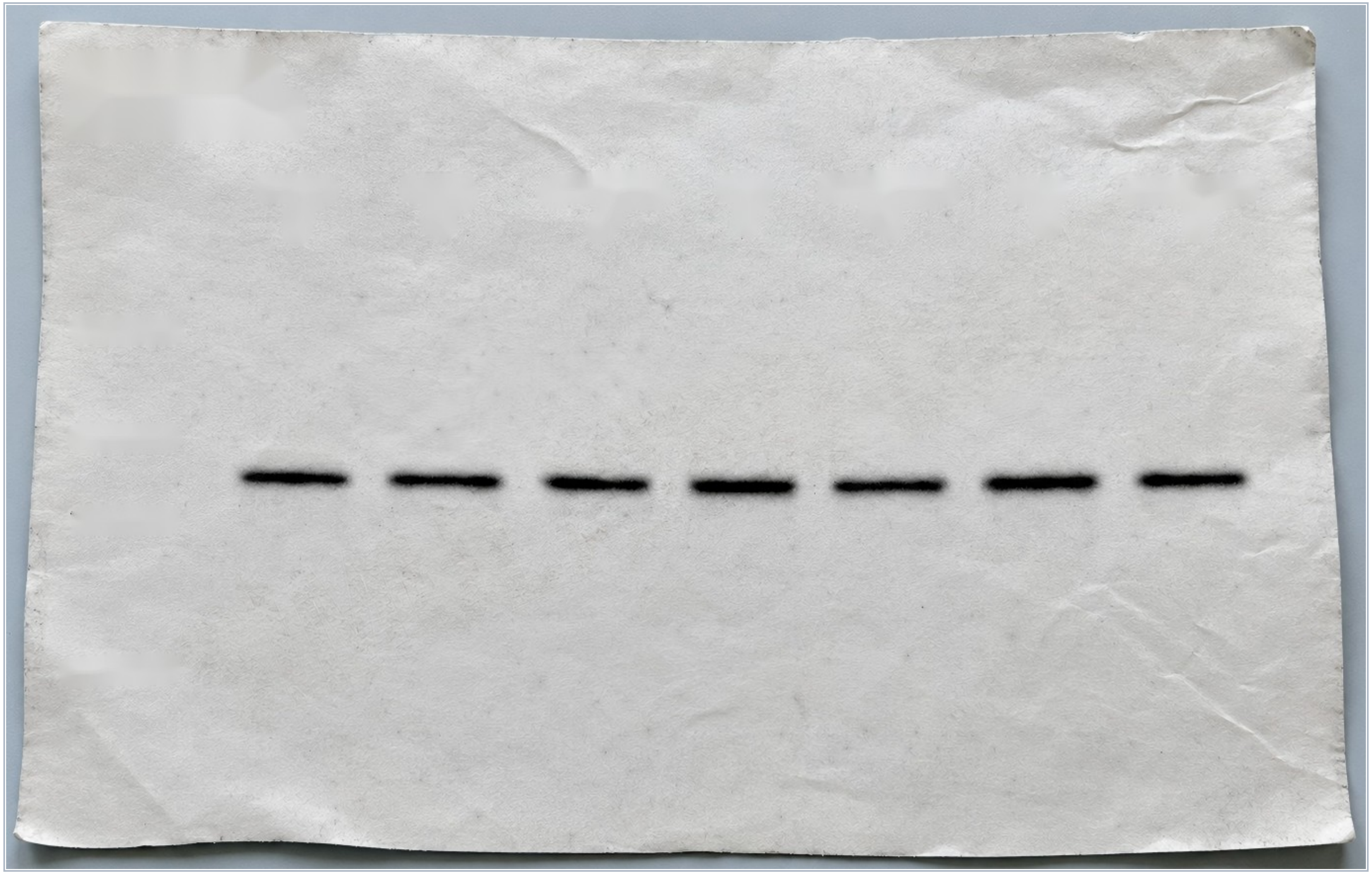

1	2	3	4	5	6	7
Control	IFN-gamma	IFN-gamma + Ruxo	IL-6	IL-6 + Stattic	OSM	OSM + Stattic

MKN45 | Biological replicate 3 | p-STAT3 (Tyr705)

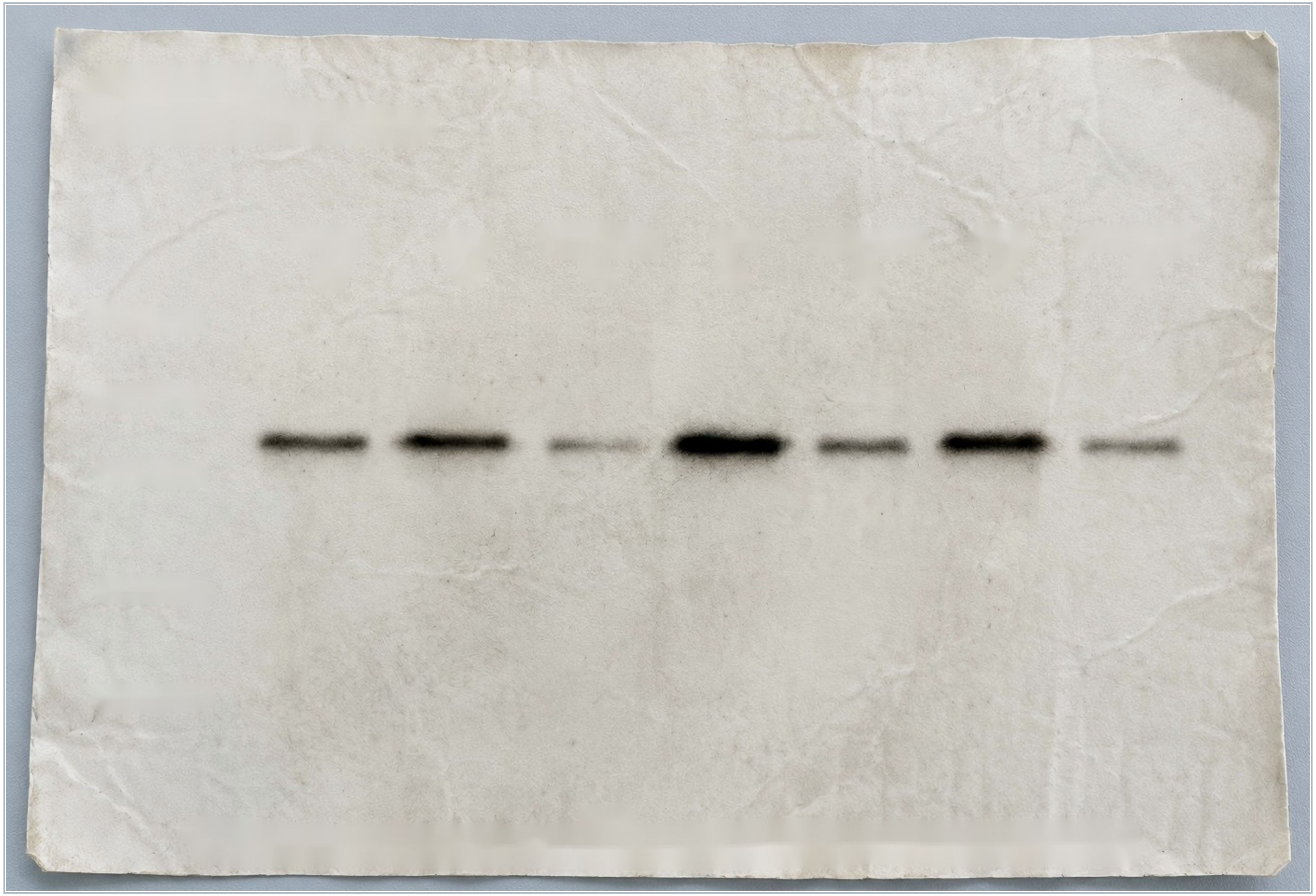

1	2	3	4	5	6	7
Control	IFN-gamma	IFN-gamma + Ruxo	IL-6	IL-6 + Stattic	OSM	OSM + Stattic

MKN45 | Biological replicate 3 | total STAT3

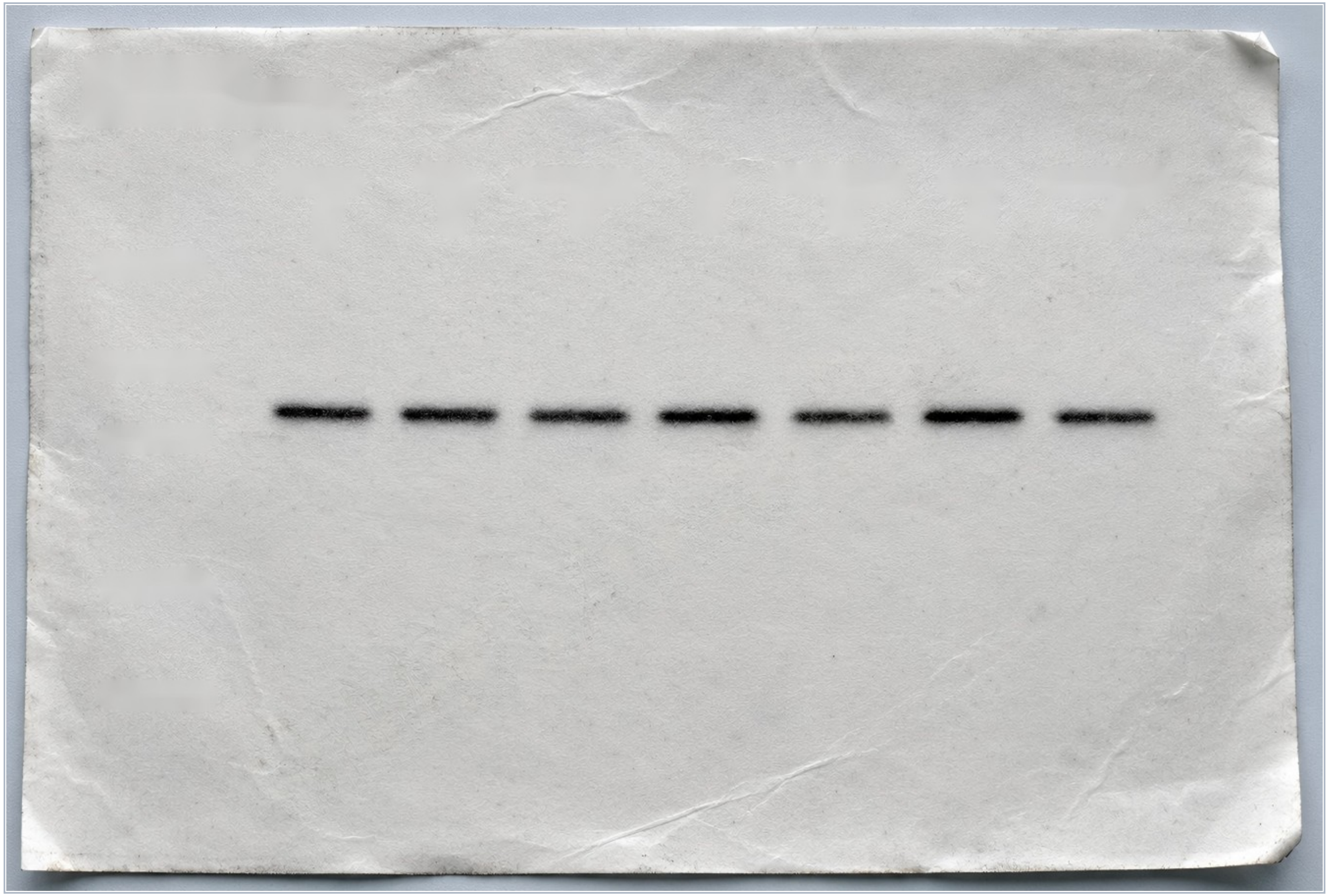

1	2	3	4	5	6	7
Control	IFN-gamma	IFN-gamma + Ruxo	IL-6	IL-6 + Stattic	OSM	OSM + Stattic

HGC-27 | Biological replicate 1 | PD-L1

This original blot corresponds to the cropped blot: HGC27_Rep1_WesternBlot

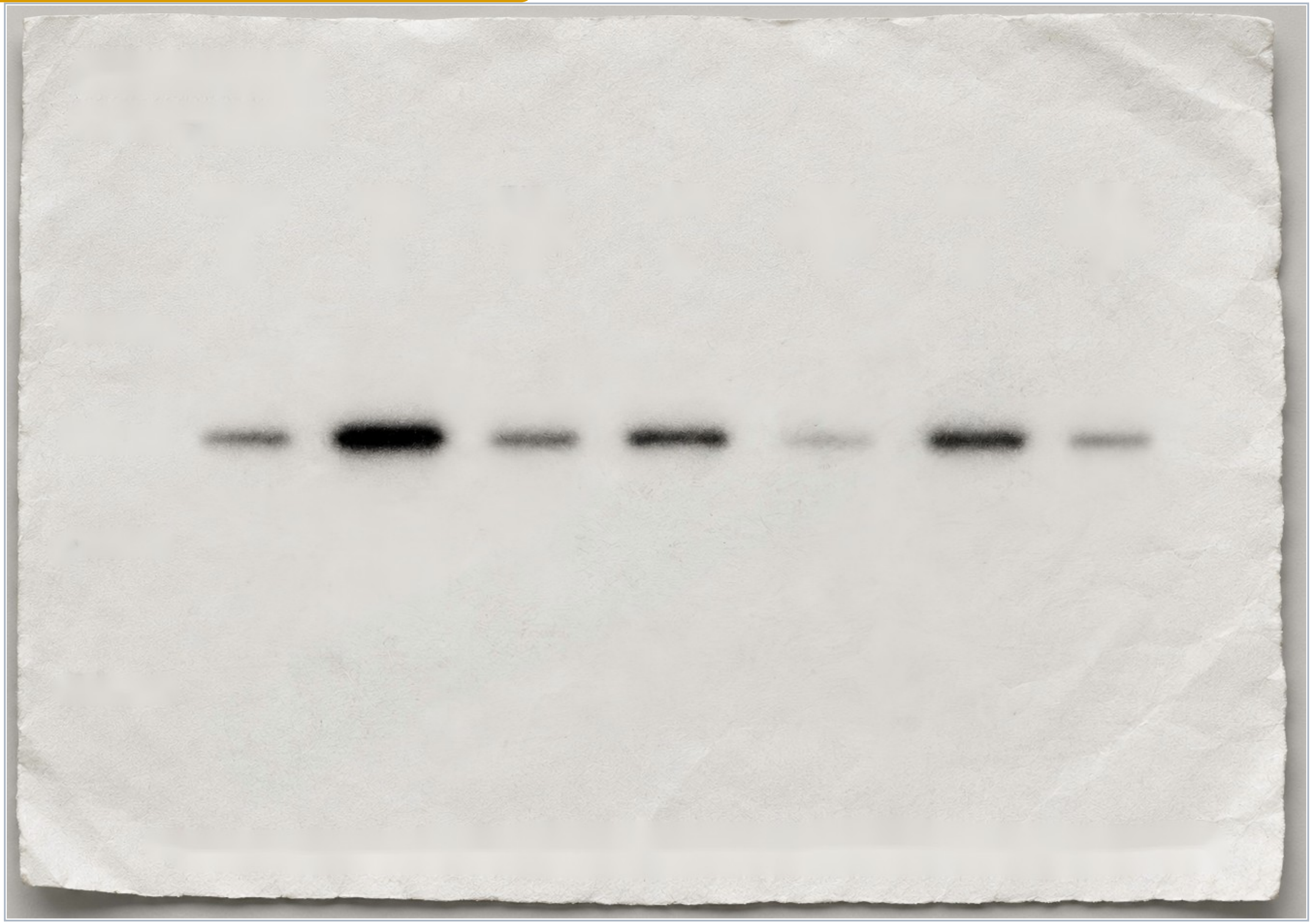

1	2	3	4	5	6	7
Control	IFN-gamma	IFN-gamma + Ruxo	IL-6	IL-6 + Stattic	OSM	OSM + Stattic

HGC-27 | Biological replicate 1 | beta-actin

This original blot corresponds to the cropped blot: HGC27_Rep1_WesternBlot

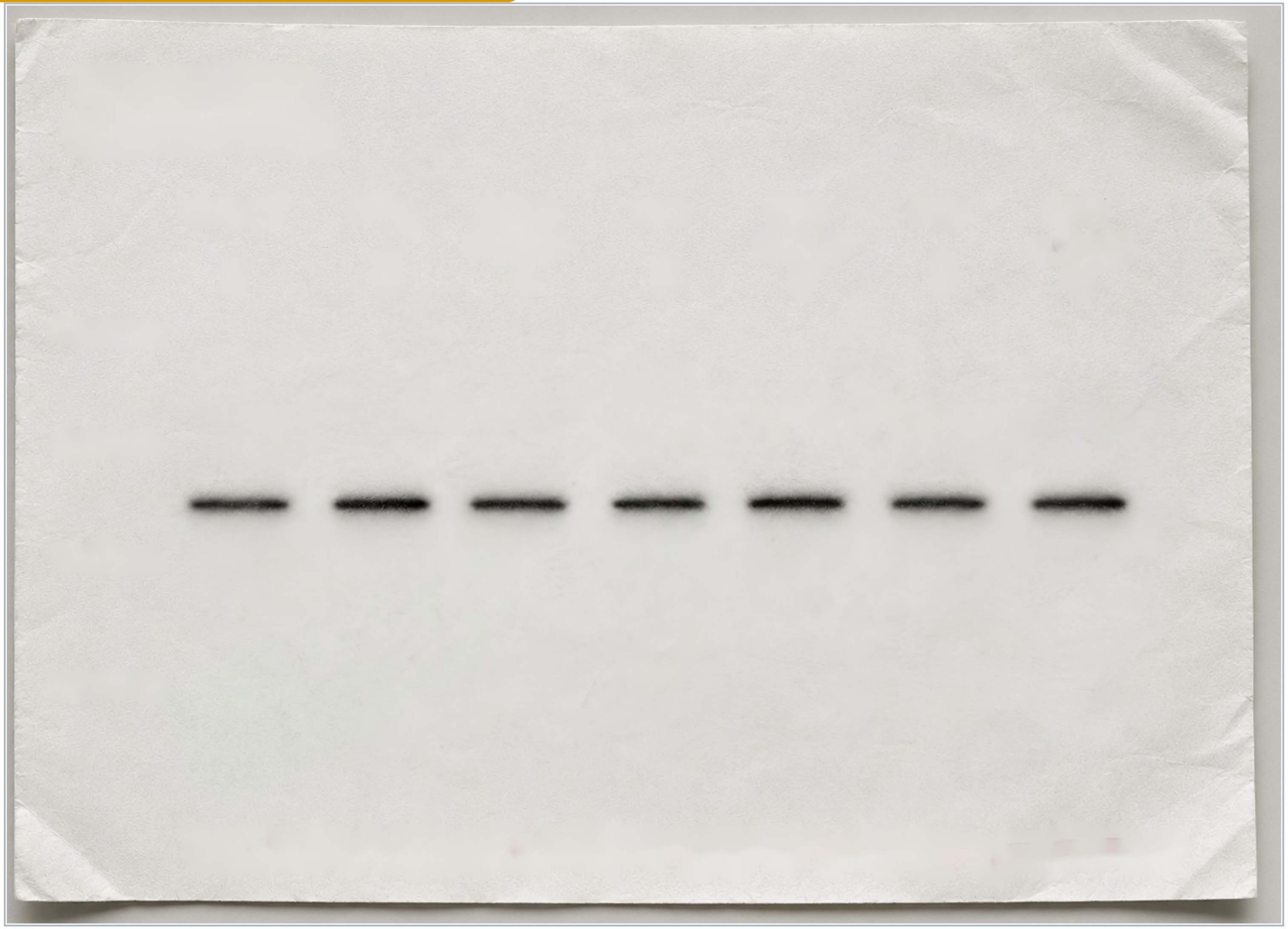

1	2	3	4	5	6	7
Control	IFN-gamma	IFN-gamma + Ruxo	IL-6	IL-6 + Stattic	OSM	OSM + Stattic

HGC-27 | Biological replicate 1 | p-STAT3 (Tyr705)

This original blot corresponds to the cropped blot: HGC27_Rep1_WesternBlot

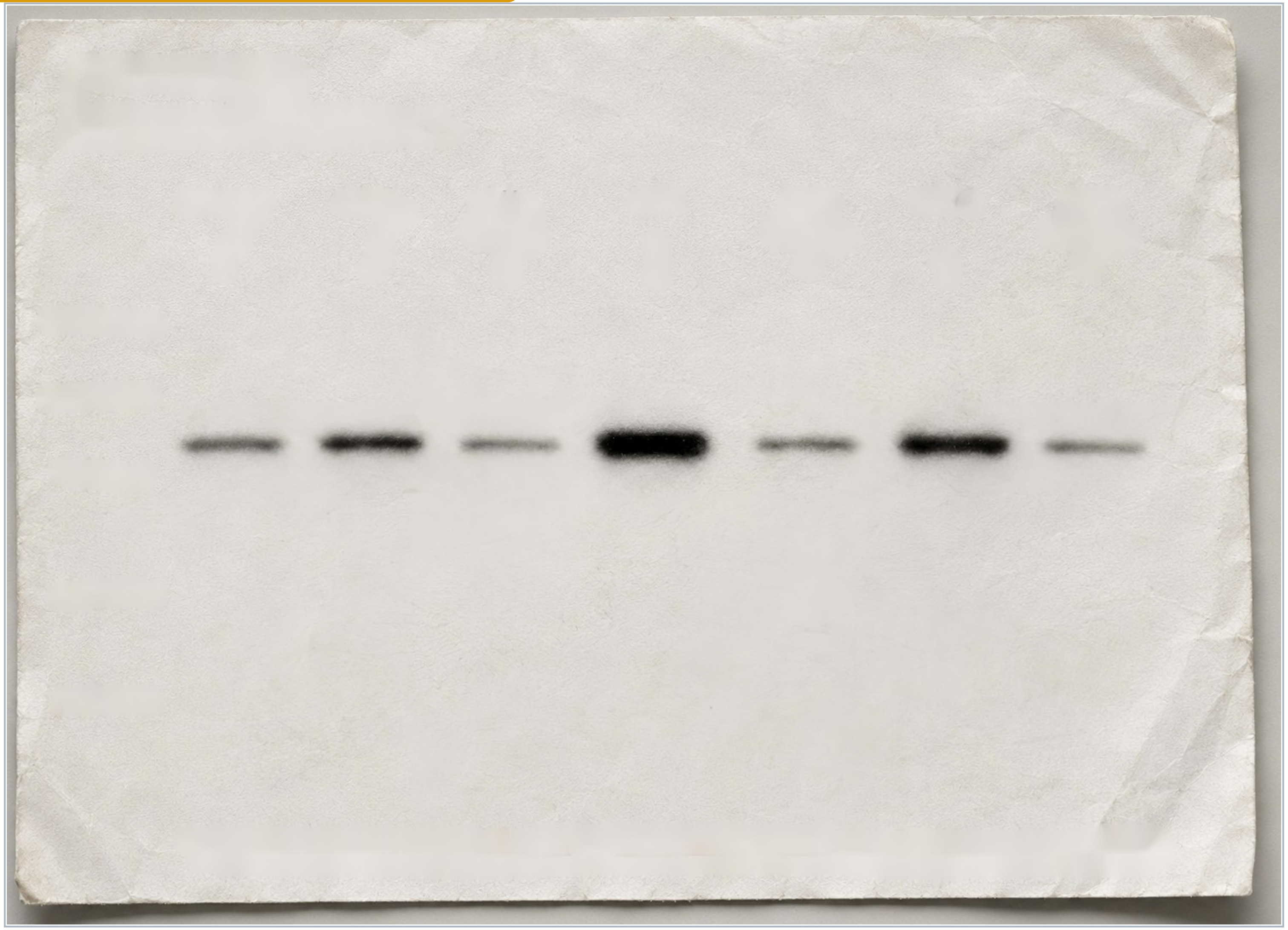

1	2	3	4	5	6	7
Control	IFN-gamma	IFN-gamma + Ruxo	IL-6	IL-6 + Stattic	OSM	OSM + Stattic

HGC-27 | Biological replicate 1 | total STAT3

This original blot corresponds to the cropped blot: HGC27_Rep1_WesternBlot

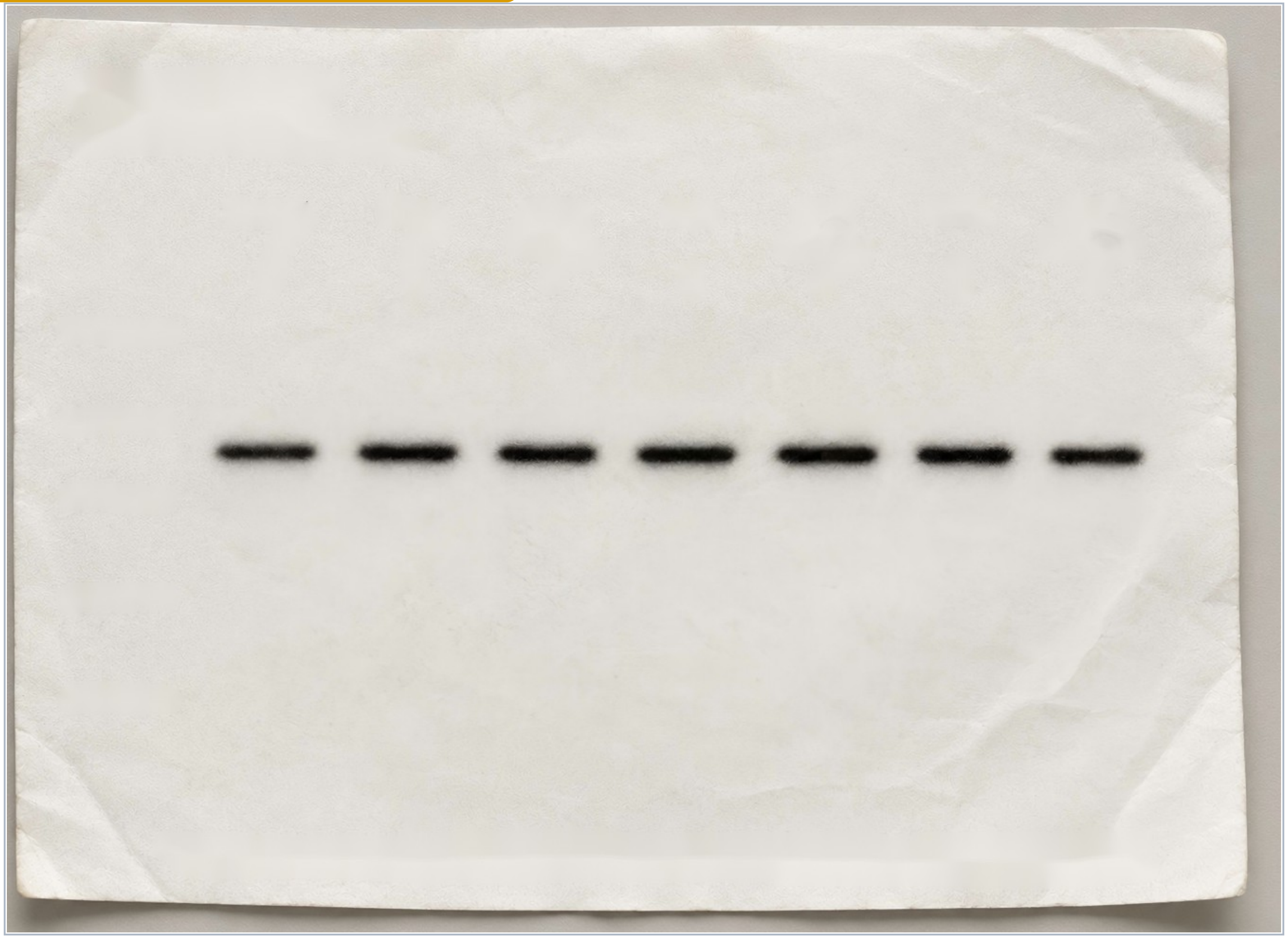

1	2	3	4	5	6	7
Control	IFN-gamma	IFN-gamma + Ruxo	IL-6	IL-6 + Stattic	OSM	OSM + Stattic

HGC-27 | Biological replicate 2 | PD-L1

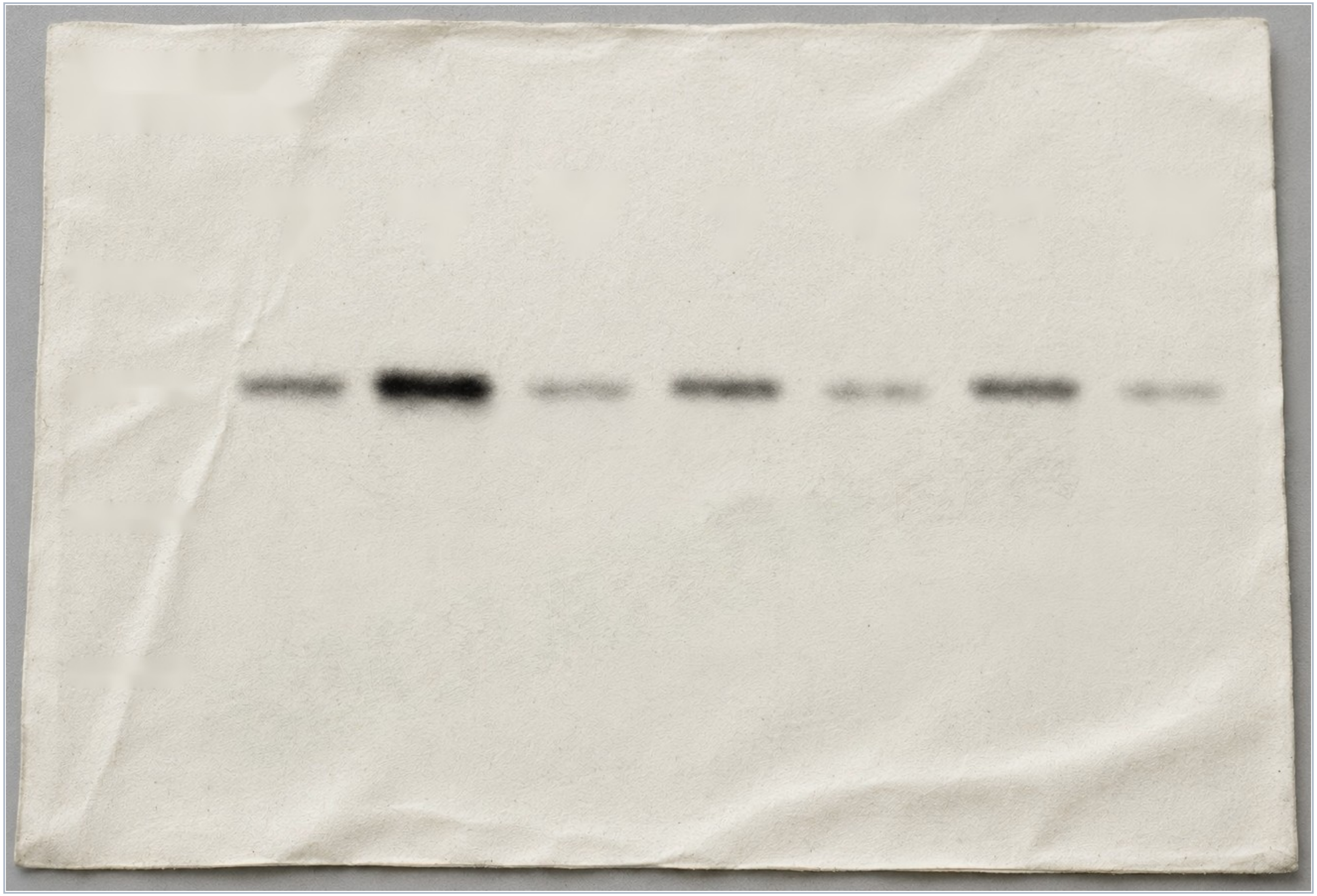

1	2	3	4	5	6	7
Control	IFN-gamma	IFN-gamma + Ruxo	IL-6	IL-6 + Stattic	OSM	OSM + Stattic

HGC-27 | Biological replicate 2 | beta-actin

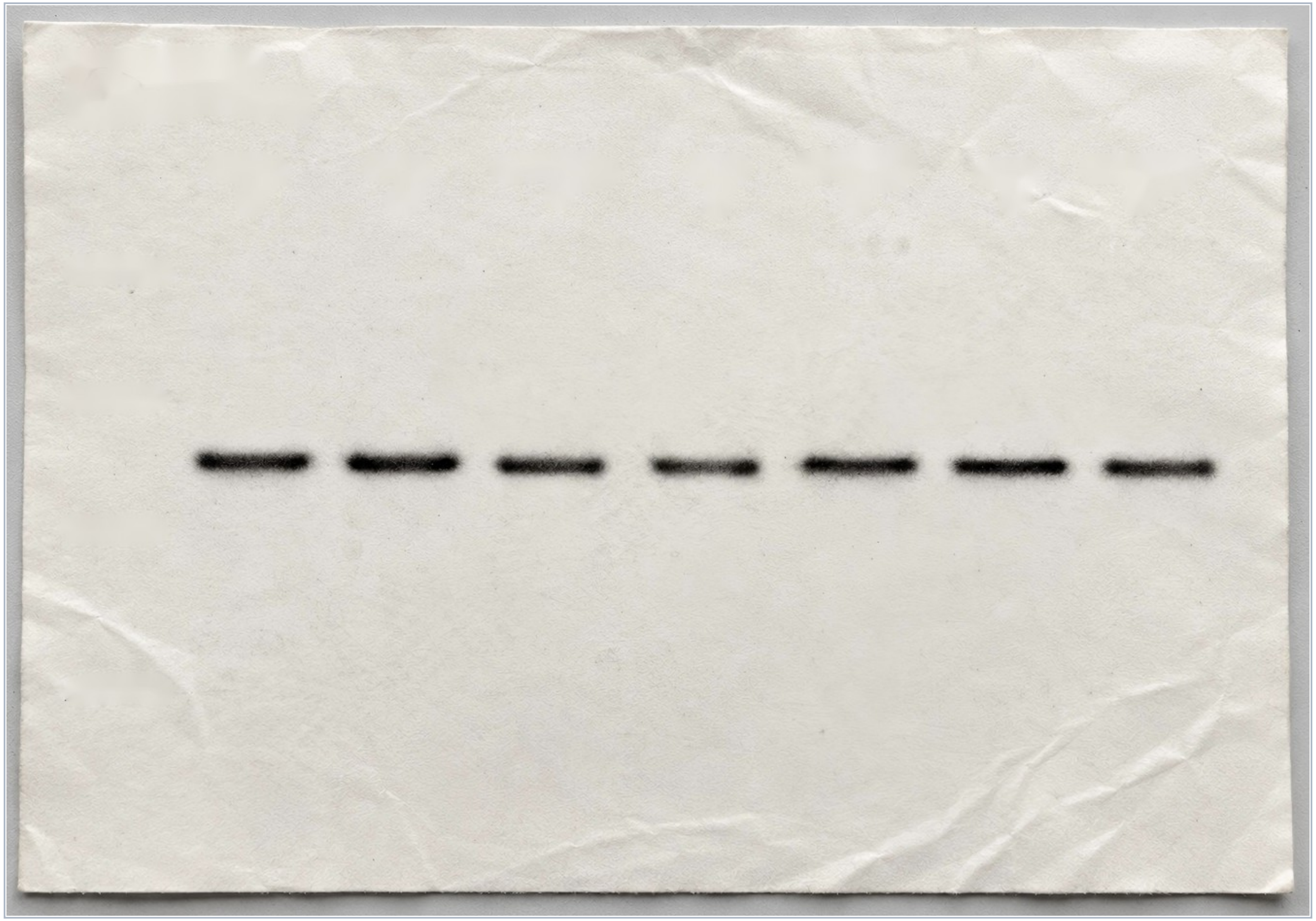

1	2	3	4	5	6	7
Control	IFN-gamma	IFN-gamma + Ruxo	IL-6	IL-6 + Stattic	OSM	OSM + Stattic

HGC-27 | Biological replicate 2 | p-STAT3 (Tyr705)

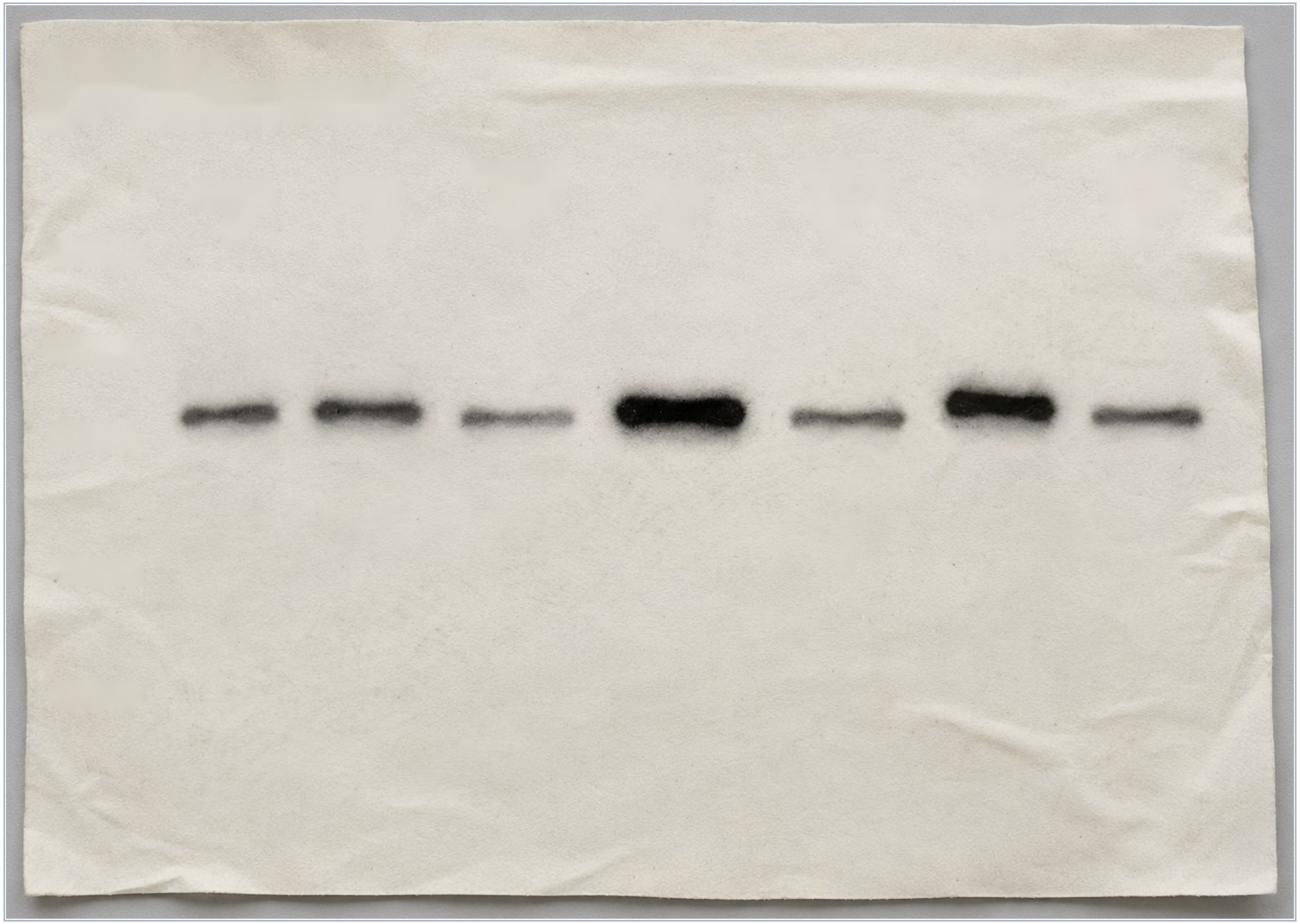

1	2	3	4	5	6	7
Control	IFN-gamma	IFN-gamma + Ruxo	IL-6	IL-6 + Stattic	OSM	OSM + Stattic

HGC-27 | Biological replicate 2 | total STAT3

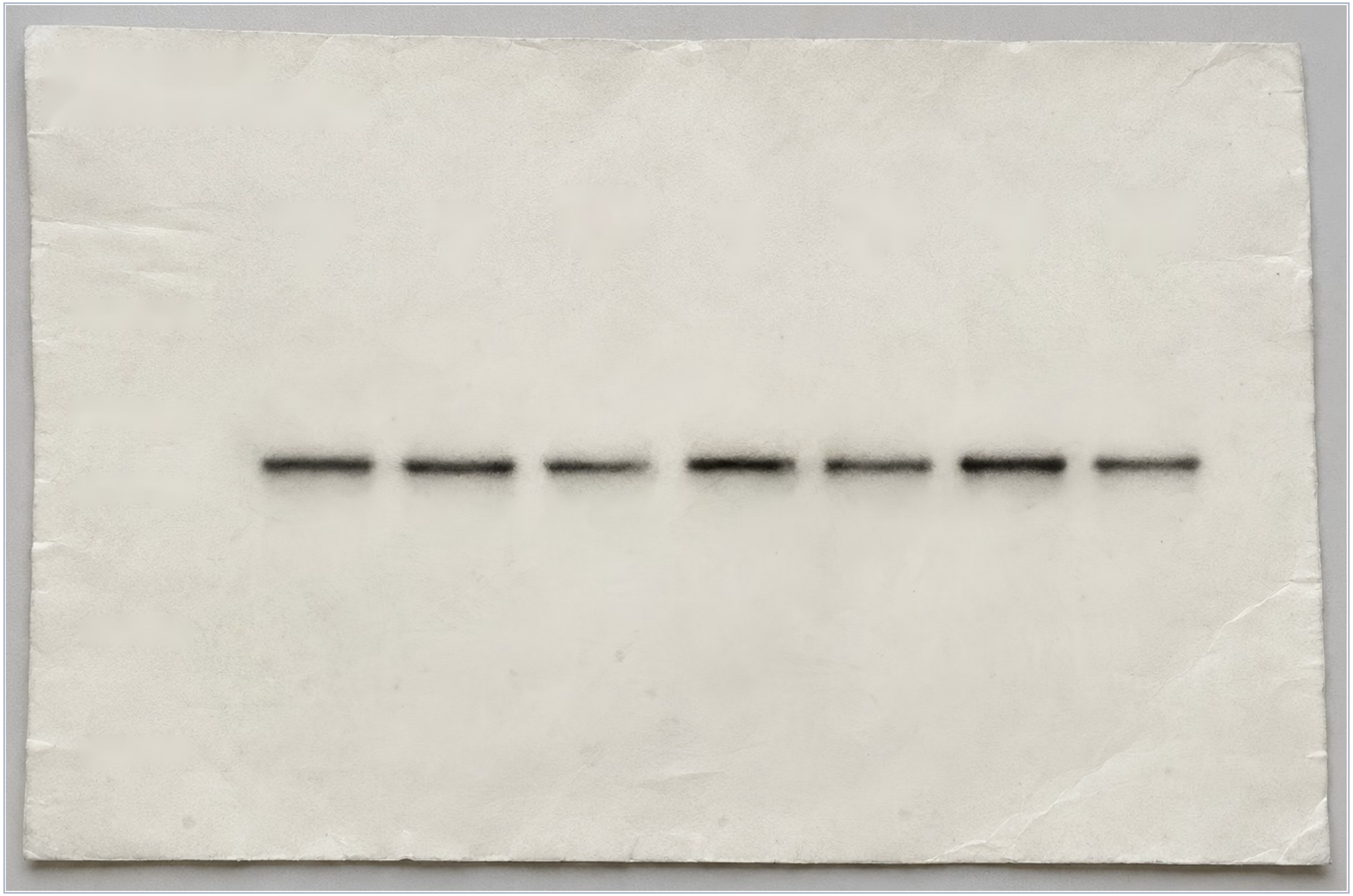

1	2	3	4	5	6	7
Control	IFN-gamma	IFN-gamma + Ruxo	IL-6	IL-6 + Stattic	OSM	OSM + Stattic

HGC-27 | Biological replicate 3 | PD-L1

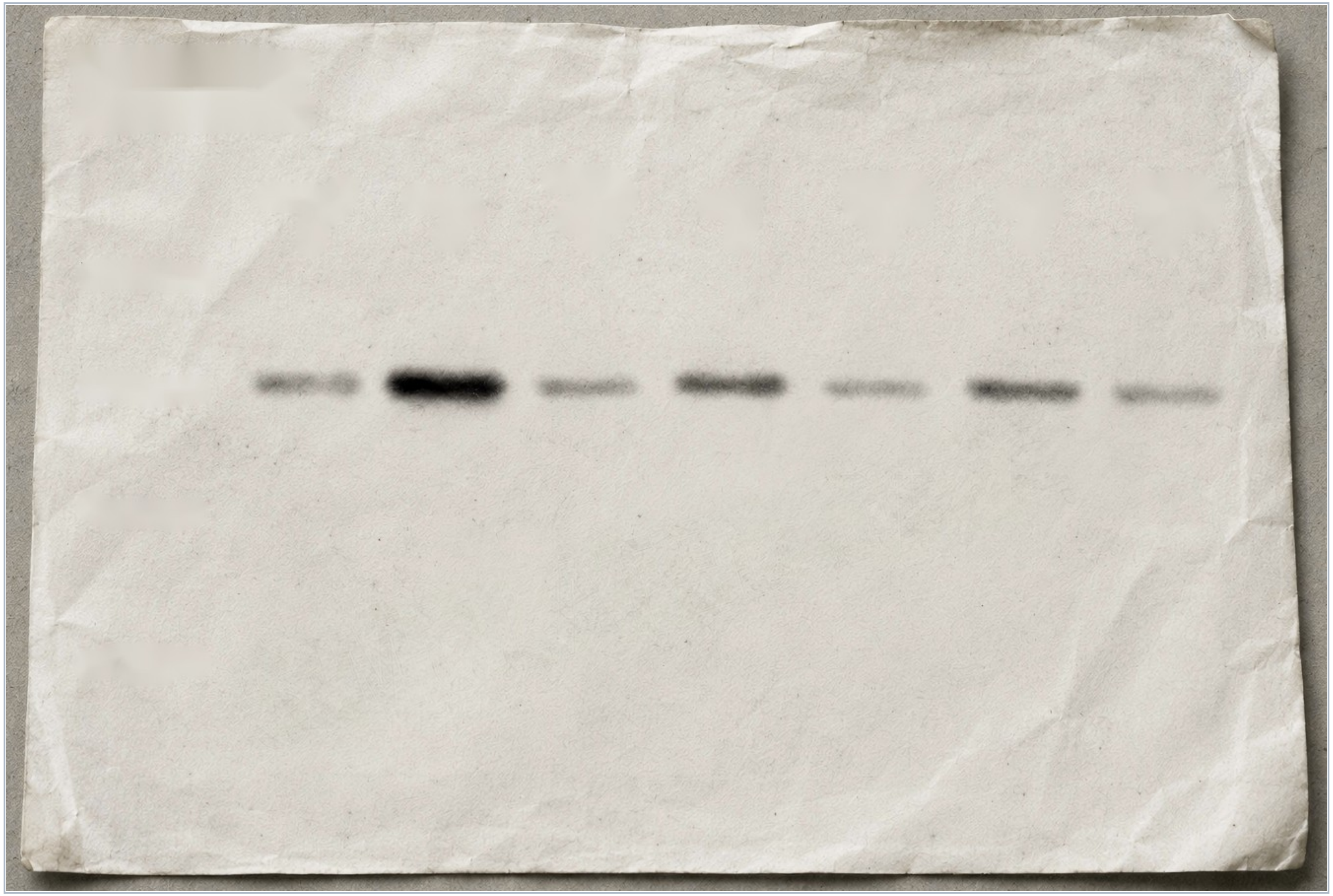

1	2	3	4	5	6	7
Control	IFN-gamma	IFN-gamma + Ruxo	IL-6	IL-6 + Stattic	OSM	OSM + Stattic

HGC-27 | Biological replicate 3 | beta-actin

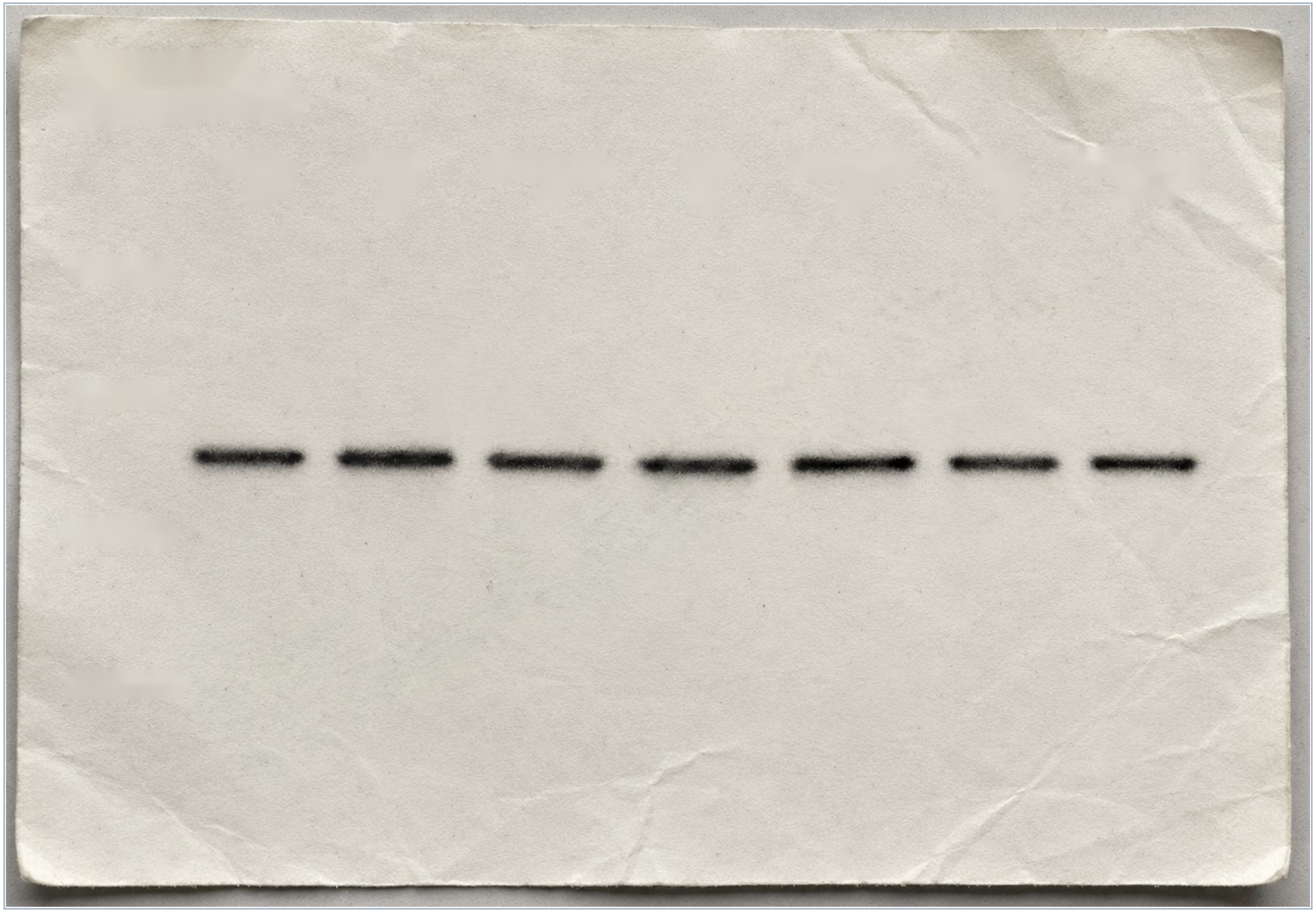

1	2	3	4	5	6	7
Control	IFN-gamma	IFN-gamma + Ruxo	IL-6	IL-6 + Stattic	OSM	OSM + Stattic

HGC-27 | Biological replicate 3 | p-STAT3 (Tyr705)

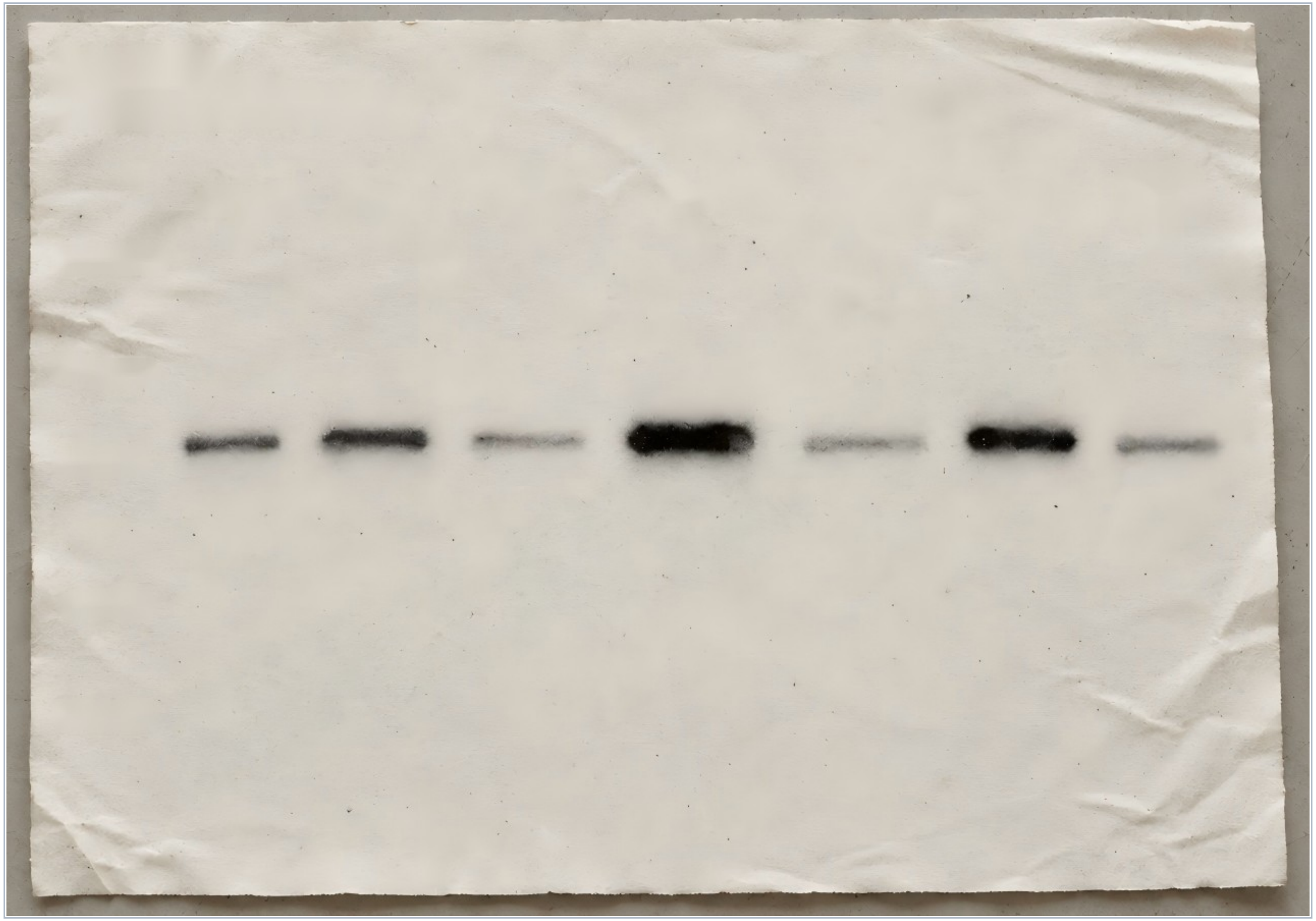

1	2	3	4	5	6	7
Control	IFN-gamma	IFN-gamma + Ruxo	IL-6	IL-6 + Stattic	OSM	OSM + Stattic

HGC-27 | Biological replicate 3 | total STAT3

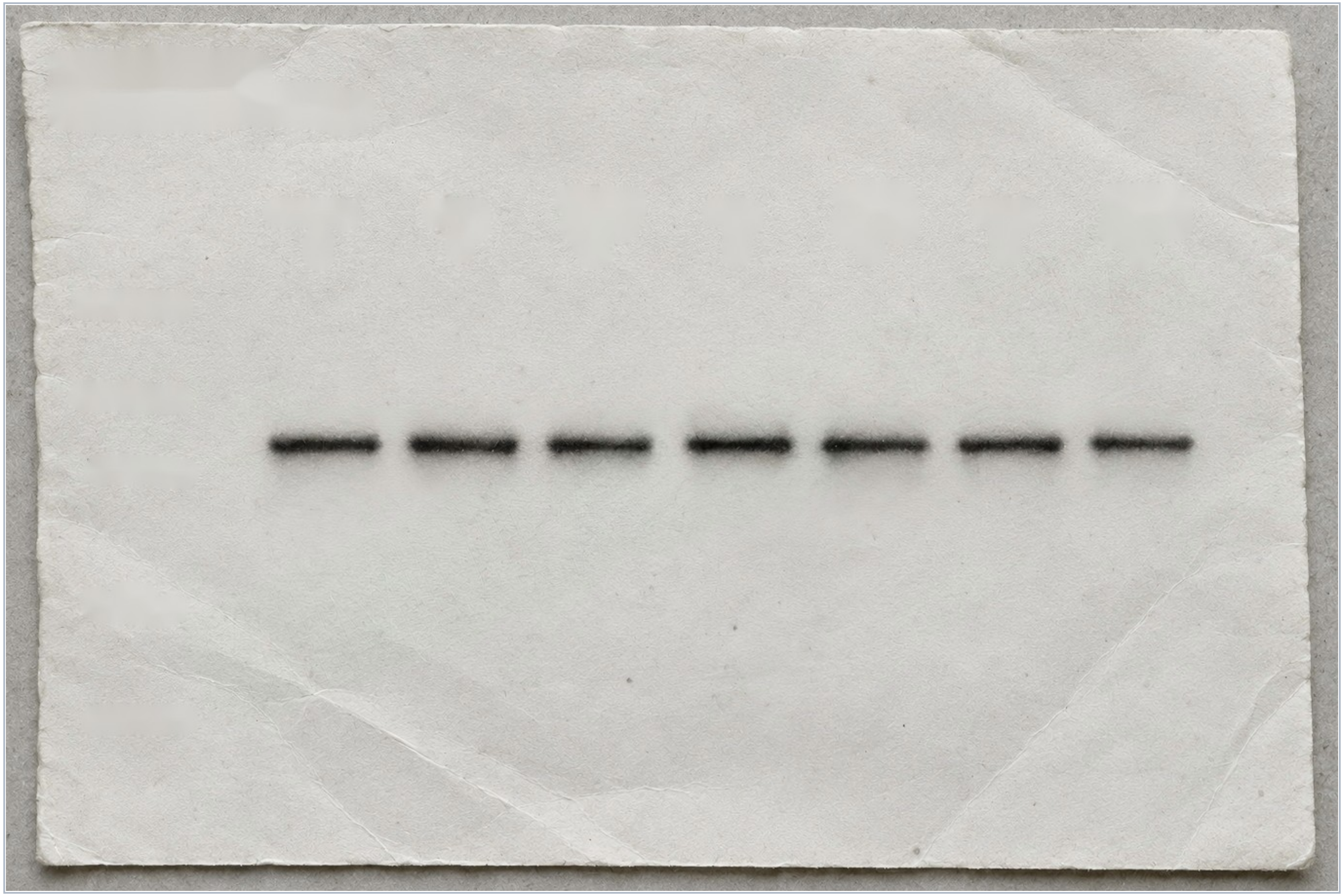

1	2	3	4	5	6	7
Control	IFN-gamma	IFN-gamma + Ruxo	IL-6	IL-6 + Stattic	OSM	OSM + Stattic
