# Supplementary material for: Single-cell and bulk transcriptome-based identification of myeloid-derived suppressor cell-associated immune evasion signatures and a prognostic model for gastric cancer
Source: BMC Cancer. 2026 Jul 29;26:1298. doi: 10.1186/s12885-026-16584-0 (PMC13700161; doi:10.1186/s12885-026-16584-0)
Supplement: Supplementary file 2 [file 12885_2026_16584_MOESM1_ESM.docx]

**Supplementary Methods**

**Supplementary Methods S1: Single-cell processing parameters**

First, quality control was performed independently for each sample based on Seurat: cells were retained if the number of detected genes per cell was 200-6000, the mitochondrial gene proportion was <20%, and the proportion of erythrocyte-related genes was <5%; scDblFinder was used to identify and remove doublets. To reduce the impact of differences in cell numbers across samples on integration, cell-number resampling balance was performed for each sample prior to merging (upper limit 1.5× the median). We then executed NormalizeData (log-normalization, scale.factor=1e4) and FindVariableFeatures (vst, nfeatures=3000) for normalization and highly variable gene selection, and integrated data objects at the patient level. Batch-effect correction used Harmony with “patient” and “sequencing batch” as batch factors (theta=2.0, max.iter.harmony=20). In the corrected low-dimensional space, we performed PCA (first 50 principal components), FindNeighbors (k=30), and Leiden clustering (resolution=0.6; FindClusters, algorithm=4), and used UMAP for visualization. After clustering, cluster-specific differentially expressed genes were identified using FindAllMarkers (Wilcoxon, min.pct=0.25, log2FC.threshold=0.25) to verify the validity of clustering and annotation; if cluster splitting due to patient heterogeneity occurred, relevant clusters were preferentially merged based on the consistency of functional module scores and core marker genes.

**Supplementary Methods S2: Cell culture conditions and cell-line information**

AGS was obtained from ATCC (CRL-1739, USA) and cultured in F-12K basal medium containing 10% fetal bovine serum and 1% penicillin–streptomycin; MKN45 was obtained from the JCRB Cell Bank (JCRB0254, Japan) and cultured in RPMI-1640 containing 10% fetal bovine serum and 1% penicillin–streptomycin; HGC-27 was obtained from CLS (Cell Lines Service GmbH, now operating under the Cytion brand; catalog number 300436, Germany) and cultured in DMEM containing 10% fetal bovine serum and 1% penicillin–streptomycin. All cells were maintained at 37°C with 5% carbon dioxide, with routine passaging at a 1:3 split ratio. All three cell lines underwent STR authentication and were confirmed mycoplasma-negative by the MycoAlert kit. Twenty-four hours before experiments, cells were seeded into 12-well plates at 1×10^5 cells per well, and cell viability had to be ≥90%.

**Supplementary Methods S3: Stimulation protocols treatment conditions and experimental procedures**

Stimulation protocols were divided into three categories: (1) Immune checkpoint induction: IFN-γ (20 ng/mL) was added for 24 h to induce CD274 expression; (2) Inflammatory–STAT3 axis induction: IL-6 (20 ng/mL), Oncostatin M (20 ng/mL), TNF-α (10 ng/mL), or IL-1β (10 ng/mL) was added for 24 h to induce IL6, CXCL8, CCL2, and PTGS2 expression; and (3) Hypoxia mimicry: CoCl₂ (150 μM) was added for 16 h to activate HIF-1α–related pathways. JAK–STAT inhibition used ruxolitinib 1 μM with 2 h pretreatment and maintained throughout stimulation; STAT3 inhibition used Stattic 5 μM with 2 h pretreatment and maintained throughout; COX-2 inhibition used celecoxib 10 μM with 2 h pretreatment and maintained throughout. The solvent control used 0.1% DMSO. Each condition had three independent biological replicates, and investigators collected data with sample labels concealed (blinded).

**Supplementary Methods S4: qRT-PCR conditions, Western blot and ELISA details**

Readouts at the gene and protein levels comprised three categories. For qRT-PCR, total RNA was extracted with TRIzol, reverse transcription was performed after gDNA-Eraser treatment, and amplification was carried out in a SYBR Green system on a LightCycler; cycling conditions were 95°C pre-denaturation for 30 s followed by 40 cycles (95°C for 5 s, 60°C for 30 s); relative expression was calculated by 2^-ΔΔCt with ACTB as the internal control. Western blot detected p-STAT3 (Tyr705), STAT3, COX-2, and PD-L1, using PVDF membrane transfer and ECL chemiluminescence; protein quantification was normalized to β-actin. ELISA quantified IL-6 and IL-8 in cell supernatants. Data from any inhibitor treatment that resulted in a reduction of cell viability >20% were excluded from analysis.

**Supplementary Figure S1. Overall study workflow from single-cell discovery to functional validation.**

**Supplementary Figure 2**. Representative Western blot analysis of PD-L1 expression and STAT3 phosphorylation in gastric cancer cell lines. AGS, MKN45, and HGC-27 cells were treated with IFN-γ, IL-6, or OSM, with or without pathway inhibitors (ruxolitinib or Stattic), as indicated. Whole-cell lysates were subjected to SDS-PAGE and immunoblotting using antibodies against PD-L1, phosphorylated STAT3 (p-STAT3, Tyr705), total STAT3, and β-actin. β-actin was used as the loading control. Representative immunoblots from three independent experiments are shown. Full-length, uncropped immunoblots are provided in the separate supplementary PDF.

**Supplementary Table S1**. Model Development and Overfitting Assessment.

| **Cohort** | **N** | **Events** | **Predictors** | **EPV** |
| --- | --- | --- | --- | --- |
| TCGA-STAD | 350 | 146 | GC-MIS, Age, Stage | 48.67 |
| GSE62254 | 300 | 127 | External validation | NA |
| GSE84437 | 433 | 191 | External validation | NA |

**Internal Validation Metrics (Training Cohort)**

| **Metric** | **TCGA-STAD** |
| --- | --- |
| Model development strategy | Prespecified |
| Variable selection method | None (a priori selected predictors) |
| Bootstrap resamples | 1,000 |
| Shrinkage factor (optimism correction) | 0.96 |
| Apparent C-index | 0.69 (0.65–0.73) |
| Optimism-corrected C-index | 0.68 (0.64–0.72) |

**Supplementary Table S2.** key clinical and platform characteristics across the three bulk cohorts.

| **Variable** | **TCGA-STAD** | **GSE62254 (ACRG)** | **GSE84437** |
| --- | --- | --- | --- |
| N | 350 | 300 | 433 |
| Age (years), mean ± SD | 64.31 ± 10.76 | 62.84 ± 11.28 | 61.69 ± 12.07 |
| Male sex, n (%) | 229 (65.43) | 196 (65.33) | 291 (67.21) |
| Stage I, n (%) | 50 (14.29) | 43 (14.33) | 46 (10.62) |
| Stage II, n (%) | 102 (29.14) | 94 (31.33) | 102 (23.56) |
| Stage III, n (%) | 160 (45.71) | 143 (47.67) | 253 (58.43) |
| Stage IV, n (%) | 38 (10.86) | 20 (6.67) | 32 (7.39) |
| Platform | RNA-seq | Affymetrix microarray | Illumina microarray |
| OS events (deaths), n (%) | 146 (41.71) | 127 (42.33) | 191 (44.11) |

**Supplementary Table S3**. Subgroup Analyses of GC-MIS and Overall Survival.

| **Subgroup** | **N** | **Events** | **HR per 1 SD increase in GC-MIS** | **95% CI** | **P value** | **Interaction P value** |
| --- | --- | --- | --- | --- | --- | --- |
| **Pathological Stage** |  |  |  |  |  |  |
| Stage I–II | 437 | 129 | 1.24 | 1.03–1.50 | 0.025 | — |
| Stage III–IV | 646 | 335 | 1.31 | 1.12–1.53 | 0.001 | 0.659 |
| **MSI Status** |  |  |  |  |  |  |
| MSI | 74 | 21 | 1.16 | 0.73–1.86 | 0.534 | — |
| MSS | 259 | 113 | 1.38 | 1.12–1.70 | 0.002 | 0.506 |
| **Lauren Classification** |  |  |  |  |  |  |
| Intestinal | 338 | 136 | 1.27 | 1.03–1.56 | 0.024 | — |
| Diffuse | 267 | 129 | 1.43 | 1.14–1.79 | 0.002 | 0.448 |
| Mixed/Other | 128 | 53 | 1.22 | 0.87–1.72 | 0.253 | 0.844 |

**Abbreviations:** GC-MIS, Gastric Cancer Myeloid Immunosuppressive Niche Score; HR, hazard ratio; CI, confidence interval; MSI, microsatellite instability; MSS, microsatellite stable.

**Note:** Hazard ratios were estimated using Cox proportional hazards regression models with GC-MIS analyzed as a continuous variable (per 1-standard-deviation increase). Interaction P values were obtained by including GC-MIS × subgroup interaction terms in Cox proportional hazards models. No statistically significant interactions were observed (all interaction P > 0.05), indicating that the prognostic effect of GC-MIS was broadly consistent across pathological stage, MSI status, and Lauren classification subgroups.

**Supplementary Table S4. Prognostic performance of GC-MIS-based models**.

**Part A. C-index (95% CI)**

| **Model** | **TCGA-STAD** | **GSE62254 (ACRG)** | **GSE84437** |
| --- | --- | --- | --- |
| Stage only | 0.64 (0.60–0.68) | 0.62 (0.58–0.66) | 0.61 (0.57–0.65) |
| Age + Stage | 0.66 (0.62–0.69) | 0.63 (0.59–0.67) | 0.62 (0.58–0.66) |
| GC-MIS only | 0.61 (0.57–0.65) | 0.59 (0.55–0.63) | 0.58 (0.54–0.62) |
| **GC-MIS + Age + Stage** | **0.69 (0.65–0.73)** | **0.66 (0.62–0.69)** | **0.64 (0.60–0.68)** |

**Part B. Time-dependent AUC (95% CI)**

| **Cohort** | **1-year AUC** | **3-year AUC** | **5-year AUC** |
| --- | --- | --- | --- |
| TCGA-STAD | 0.74 (0.68–0.79) | 0.68 (0.63–0.74) | 0.66 (0.60–0.72) |
| GSE62254 | 0.72 (0.66–0.78) | 0.67 (0.60–0.73) | 0.63 (0.57–0.69) |
| GSE84437 | 0.71 (0.66–0.77) | 0.64 (0.59–0.70) | 0.62 (0.57–0.68) |

**Supplementary Table S5**. Calibration performance of GC-MIS-based model.

| **Cohort** | **Time point** | **Calibration slope (95% CI)** | **Calibration intercept (95% CI)** | **E:O ratio** |
| --- | --- | --- | --- | --- |
| **TCGA-STAD** | 1-year | 1.02 (0.87–1.18) | 0.01 (−0.08–0.11) | 0.99 |
|  | 3-year | 0.97 (0.84–1.10) | −0.02 (−0.12–0.07) | 1.02 |
|  | 5-year | 0.93 (0.78–1.09) | −0.04 (−0.17–0.08) | 1.04 |
| **GSE62254** | 1-year | 0.96 (0.79–1.14) | 0.03 (−0.09–0.16) | 0.97 |
|  | 3-year | 0.94 (0.77–1.12) | 0.04 (−0.08–0.18) | 0.96 |
|  | 5-year | 0.89 (0.71–1.08) | 0.07 (−0.10–0.23) | 0.93 |
| **GSE84437** | 1-year | 0.93 (0.79–1.08) | −0.02 (−0.13–0.09) | 1.03 |
|  | 3-year | 0.91 (0.76–1.07) | −0.04 (−0.16–0.07) | 1.05 |
|  | 5-year | 0.87 (0.72–1.04) | −0.07 (−0.22–0.06) | 1.08 |

**Supplementary Table S6**. Reproducibility of CellChat interactions.

| **Ligand–receptor pair** | **GSE206785** | **GSE183904** | **Correlation with GC-MIS (ρ)** | **q value** | **Permutation P-value** |
| --- | --- | --- | --- | --- | --- |
| IL6–IL6R | 0.79 | 0.76 | 0.49 | 0.014 | 0.006 |
| CXCL8–CXCR1/2 | 0.87 | 0.84 | 0.52 | 0.008 | 0.004 |
| CCL2–CCR2 | 0.62 | 0.57 | 0.45 | 0.026 | 0.013 |
| VEGFA–KDR | 0.49 | 0.51 | 0.31 | 0.112 | 0.037 |
| LGALS9–HAVCR2 | 0.34 | 0.29 | 0.27 | 0.158 | 0.064 |
| CD274–PDCD1 | 0.26 | 0.23 | 0.24 | 0.217 | 0.089 |

**Supplementary Table S7**. Cohort exclusions and missing data.

| **Cohort** | **Original N** | **Excluded N** | **Final N** | **Main exclusion reason** |
| --- | --- | --- | --- | --- |
| TCGA-STAD | 375 | 25 | 350 | Missing overall survival time/status or required clinical covariates (mainly age or pathological stage) |
| GSE62254 (ACRG) | 300 | 0 | 300 | No samples excluded; all records met survival-analysis inclusion criteria |
| GSE84437 | 483 | 50 | 433 | Unavailable or unmatched overall survival follow-up/status linkage |
